# Synthesis, bonding properties and ether activation reactivity of cyclobutadienyl-ligated hybrid uranocenes†‡

**DOI:** 10.1039/d0sc05199c

**Published:** 2021-01-08

**Authors:** Nikolaos Tsoureas, Akseli Mansikkamäki, Richard A. Layfield

**Affiliations:** Department of Chemistry, School of Life Sciences, University of Sussex Brighton BN1 9QJ UK r.layfield@sussex.ac.uk; NMR Research Unit, University of Oulu P. O. Box 8000 FI-90014 Finland akseli.mansikkamaki@oulu.fi

## Abstract

A series of hybrid uranocenes consisting of uranium(iv) sandwiched between cyclobutadienyl (Cb) and cyclo-octatetraenyl (COT) ligands has been synthesized, structurally characterized and studied computationally. The dimetallic species [(η^4^-Cb′′′′)(η^8^-COT)U(μ:η^2^:η^8^-COT)U(THF)(η^4^-Cb′′′′)] (**1**) forms concomitantly with, and can be separated from, monometallic [(η^4^-Cb′′′′)U(THF)(η^8^-COT)] (**2**) (Cb′′′′ = 1,2,3,4-tetrakis(trimethylsilyl)cyclobutadienyl, COT = cyclo-octatetraenyl). In toluene solution at room temperature, **1** dissociates into **2** and the unsolvated uranocene [(η^4^-Cb′′′′)U(η^8^-COT)] (**3**). By applying a high vacuum, both **1** and **2** can be converted directly into **3**. Using bulky silyl substituents on the COT ligand allowed isolation of base-free [(η^4^-Cb′′′′)U{η^8^-1,4-(^i^Pr_3_Si)_2_C_8_H_6_}] (**4**), with compounds **3** and **4** being new members of the bis(annulene) family of actinocenes and the first to contain a cyclobutadienyl ligand. Computational studies show that the bonding in the hybrid uranocenes **3** and **4** has non-negligible covalency. New insight into actinocene bonding is provided by the complementary interactions of the different ligands with uranium, whereby the 6d orbitals interact most strongly with the cyclobutadienyl ligand and the 5f orbitals do so with the COT ligands. The redox-neutral activation of diethyl ether by [(η^4^-Cb′′′′)U(η^8^-C_8_H_8_)] is also described and represents a uranium-cyclobutadienyl cooperative process, potentially forming the basis of further small-molecule activation chemistry.

## Abstract 2

The synthesis, structure and bonding in a series of hybrid uranocenes consisting of cyclobutadienyl and cyclo-octatetraenyl ligands is described.

## Graphical abstract

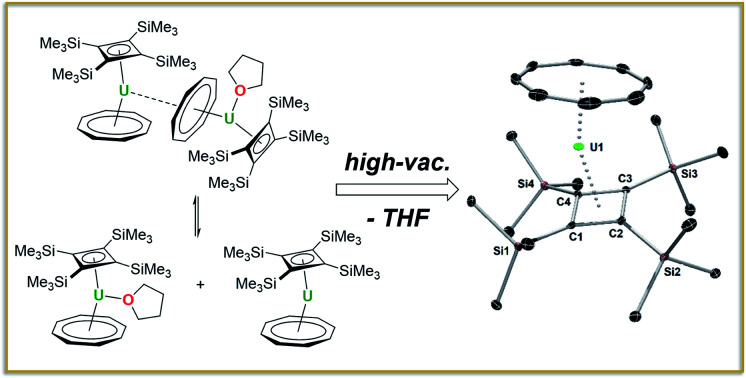

## Introduction

Cyclobutadienyl complexes of transition metals have provided a source of fascination since the early pioneering work proposing the existence of such species and, subsequently, the landmark synthesis of stable examples.^1–3^ Cyclobutadienyl complexes of the late transition metals are numerous owing to the ease with which the η^4^-bound ligands assemble through the cycloaddition of two alkynes within the coordination sphere of low-valent metals.^4–6^ Complementary routes to transition metal cyclobutadienyl complexes involving cyclization of dilithiobutadienes are also known.^7^ An understanding of the bonding properties and reactivity of transition metal-cyclobutadienyl compounds has led to their application in catalytic and stoichiometric organic synthesis.^8,9^

In contrast, cyclobutadienyl complexes of the f-elements are rare, the principal reason for which is the lack of suitable ligand sources. Relative to transition metals, the differing chemistry of lanthanides and actinides means that current methodologies are largely reliant on the use of s-block cyclobutadienyl reagents in reactions with metal halide and pseudo-halide salts.^10–15^ Since most routes to s-block cyclobutadienyl compounds involve reduction of cyclobutadienes with elemental s-block metals, the inherent instability of almost all anti-aromatic cyclobutadienes presents a challenge. However, following the seminal work of Sekiguchi *et al.*, the stable cyclobutadiene C_4_(SiMe_3_)_4_ can be conveniently synthesized and converted into the reagents [A_2_{C_4_(SiMe_3_)_4_}] (A_2_Cb′′′′, A = Li, Na, K) on a multi-gram scale.^12,16,17^ We have shown that these alkali metal reagents can transfer the bulky [{C_4_(SiMe_3_)_4_}]^2−^ dianion to lanthanides, either with the ligand remaining intact or undergoing activation processes, such as deprotonation of a silyl substituent and/or protonation of the four-membered ring, to give η^3^-allyl derivatives.^10–12^ Similar reactivity of A_2_Cb′′′′ towards uranium(iv) has also been observed, including formation of the half-sandwich complex [U(η^4^-Cb′′′′)(BH_4_)_3_]^−^ in addition to sandwich complexes containing the Cb′′′′ ligand in an activated form.^13,14^

The bonding in uranium-cyclobutadienyl complexes features non-negligible covalency, with the overlap consisting of similar contributions from the uranium 5f and 6d orbitals.^13,14,18^ This picture is reminiscent of Streitweiser's iconic cyclo-octatetraenyl (COT) sandwich compound uranocene, [U(η^8^-C_8_H_8_)_2_].^19–21^ Since uranocene and other actinocenes have played a central role in understanding covalency in actinide compounds,^22–24^ expanding the family to incorporate cyclobutadienyl ligands has the potential to provide new insight into the role of uranium valence orbitals in chemical bonding. We therefore sought to synthesize a ‘hybrid’ uranocene of the type [(η^4^-Cb)U(η^8^-COT)], with the aim of establishing whether or not the two ligand types have a preference for overlap with uranium 5f or 6d orbitals.

## Results and discussion

**Scheme 1:**
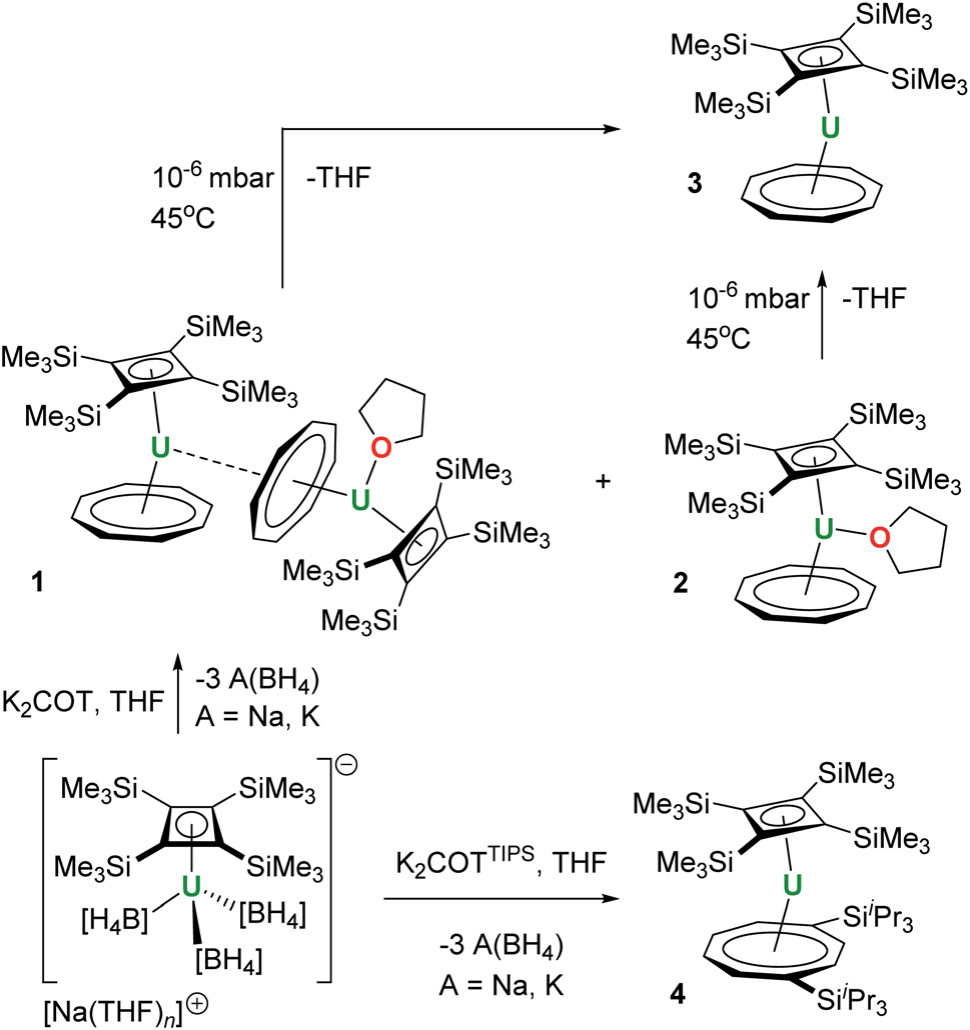
Synthesis of **1–4**.

**Fig. 1:**
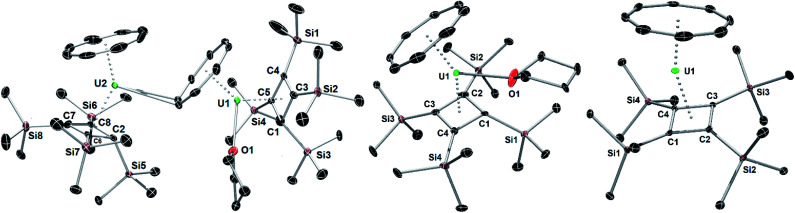
Thermal ellipsoid representations (50% probability) of the structures of **1** (left) **2** (centre) and **3** (right). For clarity, the hydrogen atoms are not shown.

We initially attempted the synthesis of [(η^4^-Cb′′′′)U(η^8^-C_8_H_8_)] by adding a slight excess (1.3 equivalents) of K_2_COT to a freshly prepared solution of Na[U(η^4^-Cb′′′′)(BH_4_)_3_]^13^ in a 2 : 1 mixture of THF-D_8_ and toluene-D_8_, which produced a brown solution. After filtration and evaporation of the solvent, the residue was extracted into *n*-heptane. Slow evaporation of the *n*-heptane under a dynamic vacuum to the point of incipient crystallization produced brown crystals, subsequently identified as the COT-bridged dimetallic compound [(η^4^-Cb′′′′)(η^8^-COT)U(μ:η^2^:η^8^-COT)U(THF)(η^4^-Cb′′′′)] (**1**, Scheme 1, Fig. 1). The nascent *n*-heptane solution obtained after crystallizing **1** was then slowly concentrated, which yielded block-like crystals of the monometallic THF-solvated species [(η^4^-Cb′′′′)U(THF)(η^8^-COT)] (**2**). Attempts at removing the THF ligand from **2**, to give the de-solvated target compound [(η^4^-Cb′′′′)U(η^8^-COT)] (**3**), by heating heptane or toluene solutions to 60 °C under a dynamic vacuum only produced intractable mixtures.^25^ However, compound **3** could be obtained from **1** or from **2** by applying a high vacuum (approximately 10^−6^–10^−7^ mbar) for five-six hours at 45 °C. We also observed that a 1 : 1 co-crystal of **2**/**3** is obtained when the initially formed crystals of **1** are washed with cold SiMe_4_ and the resulting solution is stored at −35 °C overnight.

The synthesis of the bulkier hybrid uranocene [(η^4^-Cb′′′′)U(η^8^-COT^TIPS^)] (**4**, COT^TIPS^ = 1,4-bis(triisopropylsilyl)cyclooctatetraene) proved to be more straightforward. Using the procedure described above for **1**, addition of K_2_[COT^TIPS^] to Na[U(η^4^-Cb′′′′)(BH_4_)_3_] in THF-D_8_/toluene-D_8_ produced **4**, which was isolated as crystalline material in a yield of only 12%, owing to the very high solubility of the compound even in cold SiMe_4_ (Scheme 1, Fig. 3).

The molecular structures of **1**, **2** and **3** are shown in Fig. 1 and key crystallographic parameters are listed in Table S1.‡ Both uranium(iv) centres in **1** are bound to an η^8^-COT ligand and an η^4^-Cb′′′′ ligand, with U1 also bound to a THF ligand and a μ:η^8^:η^2^-COT ligand bridging between U1 and U2. The uranium centres in **2** and **3** display similar coordination geometries to their counterparts in **1**, each with an η^8^-COT ligand and an η^4^-Cb′′′′ ligand, with **2** also featuring coordination by THF. The U–Cb_cent_ distances (‘cent’ denotes the centroid of the ring) in **1** are 2.332(4) Å (U1) and 2.340(4) Å (U2), and in **2** and **3** the distances are 2.337(2) Å and 2.323(13) Å, respectively. The U–COT_cent_ distances in **1** are 1.980(3) Å (U1) and 1.946(4) Å (U2), and 1.960(5) Å and 1.918(18) Å in **2** and **3**, respectively, hence they are markedly shorter than the corresponding distances to the Cb′′′′ ligands. The U1–COT_cent_ distance in **1** is particularly long, presumably because of the additional bridging mode adopted by the ligand. For comparative purposes, the U–COT_cent_ distance in [U(η^8^-C_8_H_8_)_2_] is 1.923(6) Å.^26^ Consistent with the uranium centres in **1** and **2** interacting with three ligands, the Cb–U–COT angles in these complexes are 139.50(14)° (U1), 139.88(16)° (U2) and 142.23(4)°, respectively, whereas the same angle in **3** is wider at 156.24(8)°, as expected based on the presence of only two ligands.

Complexes **1–3** show marked deviations of the trimethylsilyl substituents out the plane of the Cb′′′′ ring. For both uranium centres in **1**, one such substituent bends away from uranium, by 156.4(4)° (U1) and 155.8(5)° (U2), respectively, whereas the other three substituents only deviate by an average of 115.4°. In **2** and **3**, the maximum out-of-plane distortions of trimethylsilyl substituents are 142.4(4)° and 128.10(19)°, with the other three substituents also bending on average by approximately 124.6° for **2** and 117.3° for **3**. These observations reveal appreciable flexibility in the local structure of the Cb′′′′ ligand, which is presumably necessary to accommodate the structural changes that occur when additional ligands to bind to uranium, notably in **1**.

The ^1^H NMR spectrum of **1** in toluene-D_8_ at 30 °C consists of resonances at *δ* = −37.51 ppm (16 ^1^H) and −14.85 ppm (72 ^1^H), corresponding to the COT ligands and the trimethylsilyl protons of the Cb′′′′ ligands, respectively (Fig. S1 and S2‡). Resonances for the THF ligand were observed at *δ* = −30.09 and −7.26 ppm. The ^29^Si{^1^H} NMR spectrum of **1** contains a singlet at *δ* = −204.3 ppm (Fig. S3‡). In the case of THF-solvated **2**, resonances in the ^1^H NMR spectrum were observed at *δ* = −37.45 ppm and −14.01 ppm for the COT and Cb′′′′ ligands, respectively, with resonances for the THF ligand occurring at −17.26 and −4.03 ppm (Fig. S10 and S11‡). A single resonance at *δ* = −200.21 ppm was found in the ^29^Si{^1^H} NMR spectrum (Fig. S12‡). Also in toluene-D_8_ at 30 °C, the ^1^H NMR spectrum of **3** consists of resonances corresponding to the COT ligand at −37.90 ppm and the Cb′′′′ ligand at −18.45 ppm (Fig. S22 and S23‡), with the ^29^Si{^1^H} NMR spectrum featuring a single resonance at *δ* = −221.46 ppm (Fig. S24‡). The ^1^H and ^29^Si{^1^H} NMR spectra of the co-crystal consist of resonances similar to those observed for isolated **3**, however signals for the THF ligand were not observed, presumably due to rapid exchange between uranium centres (Fig. S32 and S33‡).

**Fig. 2:**
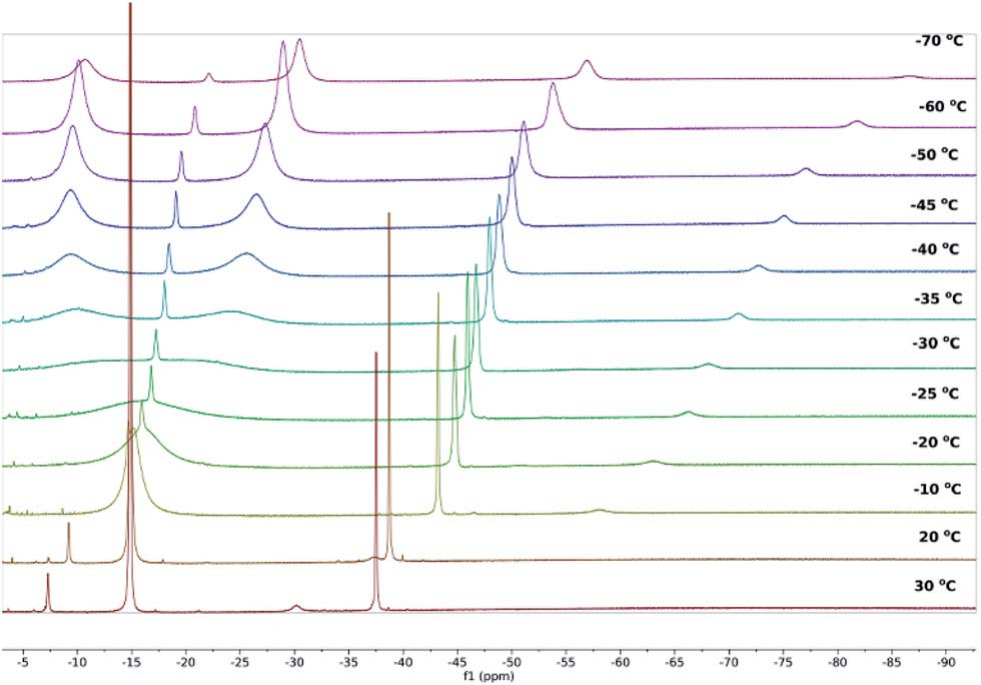
^1^H NMR spectra of **1** in toluene-D_8_ at the temperatures indicated.

Whereas the ^1^H NMR spectra of **2** and **3** in toluene at 30 °C indicate that the solid-state molecular structures of the complexes are retained in solution, the ^1^H NMR spectrum of **1** suggests the occurrence of dynamic behaviour under these conditions. A variable-temperature ^1^H NMR spectroscopic study of **1** revealed that the sharp singlet corresponding to the SiMe_3_ environment broadens on cooling and decoalesces below −30 °C, resolving into two additional singlets at *δ* = −9.65 and −27.20 ppm at −50 °C, each corresponding to 36 ^1^H (Fig. 2 and S4–S6‡). The ^29^Si{^1^H} NMR spectrum of **1** also resolves into two broad singlets at −50 °C, with *δ* = −209.94 ppm (FWHM = 709 Hz) and −286.54 ppm (FWHM = 669 Hz), respectively (Fig. S7‡). The most probable explanation for this behaviour is that **1** exists in a dynamic equilibrium with **2** and **3**, which is fast relative to the NMR timescale at 30 °C. To confirm this, we recorded the ^1^H and ^29^Si{^1^H} NMR spectra of isolated **2** and **3** at low temperatures and found very close matches in the chemical shifts compared with those of **1** at the same temperatures (Fig. S13–S15 and S25–S27‡). At −50 °C, the ^1^H and ^29^Si{^1^H} chemical shifts of the trimethylsilyl substituents in **3** occur at −9.69 ppm and −213.08 ppm, respectively, and in the case of **2** the analogous ^1^H and ^29^Si{^1^H} resonances were observed at −27.77 ppm and −285.13 ppm, respectively (Fig. S28 and S29‡).

**Fig. 3:**
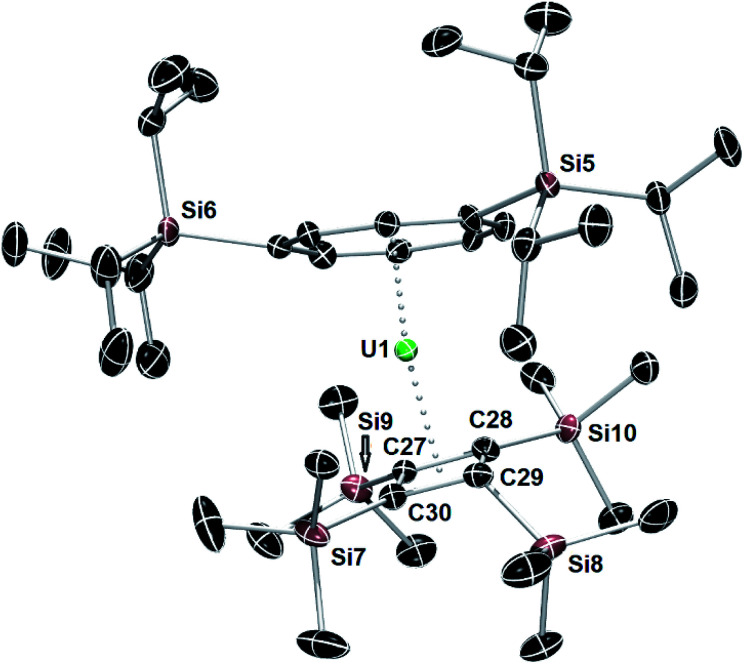
Thermal ellipsoid representations (50% probability) of the molecular structures of **4**. For clarity, the hydrogen atoms are not shown.

The structure of the hybrid uranocene **4** comprises two crystallographically unique molecules with very similar geometric parameters, only one of which is described in detail here (Fig. 3). Molecule 1 of **4** consists of an η^8^-COT^TIPS^ ligand and an η^4^-Cb′′′′ ligand, with distances to the centroid of each ligand of 1.916(2) Å and 2.334(3) Å, respectively. The associated bending angle at uranium is 152.79(12)°. The U–COT_cent_ distances in **4** are somewhat shorter than those observed in the related series of uranium(iv) sandwich complexes [(η^8^-COT)U(X)(η^5^-C_5_Me_5_)] (X = H, NH_2_, HCO_2_, H_2_NCO_2_), which lie in the range 1.93–1.97 Å.^27,28^ Exclusion of a third ligand from the uranium centre in **4** is presumably due to the bulk of the tri-isopropylsilyl substituents. Three of the trimethylsilyl substituents in **4** deviate appreciably from the plane of the Cb′′′′ ring by 127.9(3)–147.6(4)°, however the substituent containing Si7 deviates from the plane only by 105.8(3)°, which is markedly less than observed for all analogous substituents in **1–4**. This structural feature may facilitate close approach of a methyl group to the uranium centre, but this is not necessarily indicative of an agostic interaction.^29^

The ^1^H NMR spectrum of **4** in toluene-D_8_ is fully consistent with the solid-state structure, consisting of a 36-proton singlet at −9.37 ppm for the Cb′′′′ ligand, three two-proton singlets at −156.23, −99.09 and +103.80 ppm for the COT protons, and resonances at −4.24, −0.14 and 2.23 ppm for the Si^i^Pr_3_ groups (Fig. S34 and S35‡). Two resonances in the ^29^Si{^1^H} NMR spectrum were observed at *δ* = −139.63 and −33.80 ppm (Fig. S36‡).

To gain further insight into the energetics of THF coordination to the uranium(iv) centres in the hybrid uranocenes, the free energies for the formation of **2** from **3** and THF, and formation of the hypothetical complex **4**·THF from **4** and THF, were calculated at the DFT level using the pure PBE functional.^30,31^ To ensure that the computational protocol provided reasonably accurate values, the complexation energy for the formation of **2** was also calculated using the high-level domain-localized pair natural orbital coupled cluster (DLPNO-CCSD) approach (Tables S2 and S3‡).^32–36^ The calculated free energies for the formation of **2** and **4**·THF are −81 kJ mol^−1^ and −4 kJ mol^−1^, respectively. Thus, the formation of **2** is very favorable whereas the free energy gained upon complexation of **4** by THF is negligible, considering the typical accuracy of DFT energetics.

The differences in the energetics can be understood by partitioning the free energy of formation into contributions from fragment distortion (Δ*E*_dist_), orbital interaction (Δ*E*_orb_), dispersion (Δ*E*_disp_), and enthalpy and entropy contributions (Δ*H* − *T*Δ*S*). The distortion energy corresponds to the energy required to distort the geometries of the **3** and **4** and the THF ligand from relaxed structures to the geometry they possess in the adducts. The orbital interaction energy describes the energy lowering once the electron densities of the two distorted fragments mix and relax. The contributions are listed in Table 1. The distortion energy in **4**·THF is at 85 kJ mol^−1^, much larger than in **3**, most likely due to the additional steric bulk associated with the Si^i^Pr_3_ substituents. In addition, the Δ*H* − *T*Δ*S* contribution is unfavorable for the complexation of **4** by THF to give **4**·THF, which is mostly due to a largely unfavorable entropy contribution, unlike with the formation of **2** from **3**.

**Table 1:** Energy contributions and free energies (in kJ mol^−1^) for the formation of **2** and **4**·THF from **3** + THF and **4** + THF, respectively

	**2**	**4**·THF
Δ*E*_dist_	29	85
Δ*E*_orb_	−56	−64
Δ*E*_disp_	−41	−54
Δ*H* − *T*Δ*S*	−12	30
Δ*G*	−81	−4

The nature of the orbital interactions between the uranium(iv) ion and the Cb′′′′ and COT ligands in **3** and **4** was further studied by decomposing the DFT orbitals, calculated using the hybrid PBE0 functional,^30,31,37,38^ into relative contributions from the uranium and ligand fragment orbitals. The bonding in **3** and **4** is qualitatively similar, with the Si^i^Pr_3_ substituents having only a minor quantitative effect overall, hence only **3** is discussed in detail. Quantitative values of the orbital composition are given in Tables S4 and S5.‡ The metal–ligand interaction is dominated by electron donation from the two highest occupied and nearly degenerate orbitals of both the Cb′′′′ and COT ligands. Both are degenerate under ideal symmetry and we refer to the set of two near-degenerate orbitals as the HOMO. The electron donation from the ligands takes place both to the partially occupied 5f shell and the empty 6d shell. These interactions account for over 86% of the orbital compositions in all valence molecular orbitals discussed here.

**Fig. 4:**
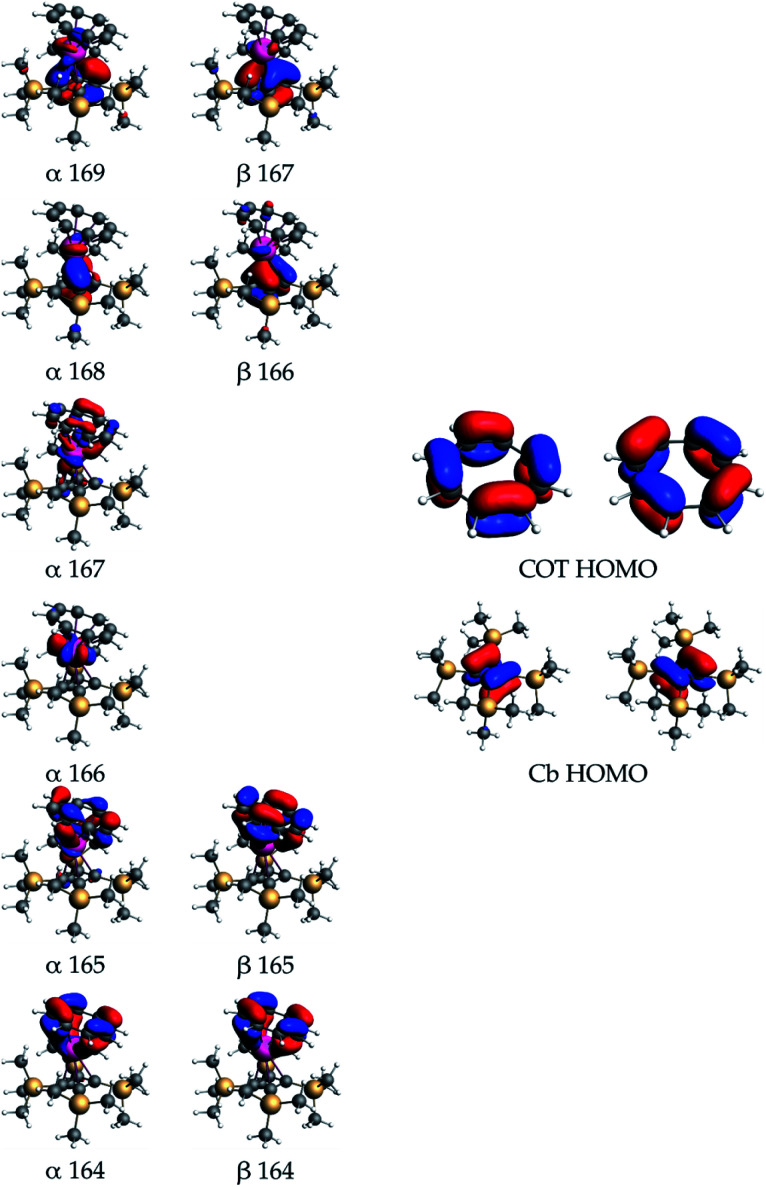
Important valence orbitals of **3**.

The quantitative contributions are listed in Table S4‡ and the respective orbitals are shown in Fig. 4.

The mixing of the ligand and uranium 5f orbitals in **3** is more significant than in the previously reported uranium(iv) cyclobutadienyl complexes, such as [(Cb′′′′)U(BH_4_)_3_]^−^.^13^ Qualitative examination of the orbital contributions shows that the mixing between the Cb′′′′ ligand orbitals and the 6d orbitals is more significant than that between the ligand and 5f orbitals, whereas the COT-5f interactions are more significant than the COT-6d interactions. The α orbitals 168 and 169, and the β orbitals 166 and 167 primarily describe the U–Cb′′′′ covalency. The contributions from the Cb′′′′ HOMOs to the orbital composition vary between 54% and 67%, the 5f contributions between 6% and 21%, and the 6d contributions between 6% and 18%. The α and β orbitals 164 and 165 primarily describe the U–COT covalency, with the contributions from the COT HOMOs varying between 44% and 73%, the 5f contributions between 2% and 32% and the 6d contributions between 8% and 13%. Of the two unpaired 5f electrons only one occupies an orbital with strong atomic 5f-like character, *i.e.* α 166 with 90% 5f character. The only other orbital with significant 5f character is the α orbital 167, which is strongly mixed with the COT HOMOs and has 47% 5f character.

The synthesis and isolation of [(η^4^-Cb′′′′)U(η^8^-COT)] (**3**) and [(η^4^-Cb′′′′)U(η^8^-COT^TIPS^)] (**4**) furnishes new members of the f-element organometallic sandwich family, which includes Streitweiser's first-generation uranocene [U(η^8^-COT)_2_]^20^ in addition to the divalent uranocene [U(η^5^-C_5_^i^Pr_5_)_2_]^39,40^ and the cycloheptarienyl complex [U(η^7^-C_7_H_7_)_2_]^−^,^41^ amongst others.^42^ The hybrid uranocenes **3** and **4** are the first bis(annulene) actinocenes containing a cyclobutadienyl ligand. The tendency for the uranium(iv) centers in **1–3** to acquire a third ligand, such as THF or μ-COT, contrasts to the behavior of uranocenes of the type [U(η^8^-COT^R^)_2_] (R denotes various alkyl, aryl and silyl substituents), in which uranium generally resists additional complexation.^43–46^ This property of **1–3** has also been observed in the oligomeric thorium(iv) paddle–wheel complex [Th(η^4^-Cb′′′′)(μ:η^8^-COT)(μ:η^2^-COT)(K)_2_(toluene)_5_]_2_,^47^ which contains thorium in a geometry similar to that observed for one of the uranium centers in **1**. The observations on **1–4** suggest that our hybrid uranocenes show different reactivity than first-generation uranocenes towards Lewis bases, which is explored further below.

Analysis of the metal–ligand orbital interactions in **3** and **4** revealed significant covalency. The significant interaction of the Cb′′′′ ligand with the uranium 6d orbitals relative to its interaction with the 5f orbitals is complementary to the interaction of uranium with COT ligands, where the 5f orbital overlap is stronger than with the 6d orbitals. The overall bonding scenario is therefore somewhat different to that calculated for the actinocenes [An(COT)_2_]^*n*^, in which An is the trivalent (*n* = −1) and tetravalent (*n* = 0) cations of uranium, neptunium and plutonium, where the covalency involves overlap of COT orbitals with metal 5f and 6d orbitals, but with greater contributions thought to originate from the latter.^23,24,48–52^ Based on our calculations, we predict that the metal–ligand bonding in homoleptic cyclobutadienyl sandwich complexes with the general formula [An(η^4^-C_4_R_4_)_2_]^*n*^ (An = U, Np, Pu; *n* = 0, −1) should tip the balance of 5f and 6d orbital involvement further towards the latter. If correct, this would presumably result in the 5f orbitals in these molecules adopting stronger atomic 5f character, which may lead to interesting single-molecule magnet behavior for 5f^3^ species such as [U(η^4^-C_4_R_4_)_2_]^−^, assuming they can be synthesized.^53^

**Scheme 2:**
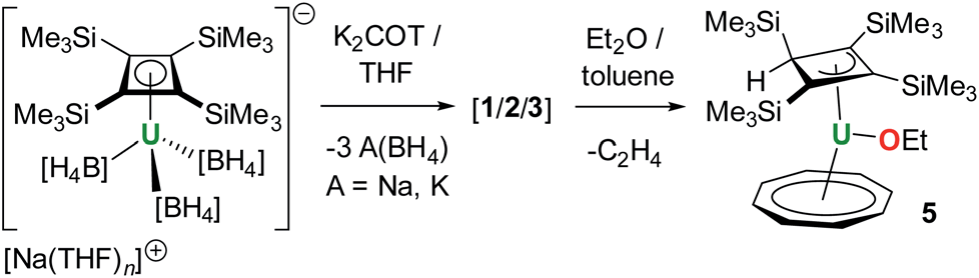
Synthesis of **5**.

During our efforts to optimize the synthesis of **1–3**, we observed that, after extracting the reaction mixture obtained from mixing K_2_COT and Na[U(η^4^-Cb′′′′)(BH_4_)_3_] into ether/toluene, a gradual colour change from brown to dark red occurred within a few hours. Analysis of the mixture by ^1^H and ^29^Si{^1^H} NMR spectroscopy revealed the formation of a new product in >90% yield (Fig. S39–S41‡). The ^1^H NMR spectrum in benzene-D_6_ of the crystalline material subsequently isolated from the reaction (Fig. S42–S44‡) features three peaks for the SiMe_3_ substituents at −21.37, −3.26 and +16.87 ppm integrating in the ratio 18 : 9 : 9, respectively, while the COT protons resonate at −32.54 ppm. In addition, resonances were observed at +25.97 ppm, +45.52 ppm and +94.77 ppm, integrating in the ratio 3 : 1 : 2. The ^29^Si{^1^H} NMR spectrum displays three peaks at −255.64, −64.11 and +37.87 ppm (Fig. S45‡). These data indicate that the local four-fold symmetry of the Cb′′′′ ligand has been lowered to *C*_s_ symmetry in solution. On this basis, we hypothesized that the new product contains the protonated, η^3^-allylic version of Cb′′′′, which forms concurrently with activation of the ether solvent to give an ethoxide ligand (Scheme 2). Further support for this proposals was obtained from an EI-MS analysis of the isolated material, which shows a peak at 729 Da, consistent with the formulation [{(Cb′′′′)U(COT)} + EtO + H], which also displays the correct isotopic envelope (Fig. S47‡).

**Fig. 5:**
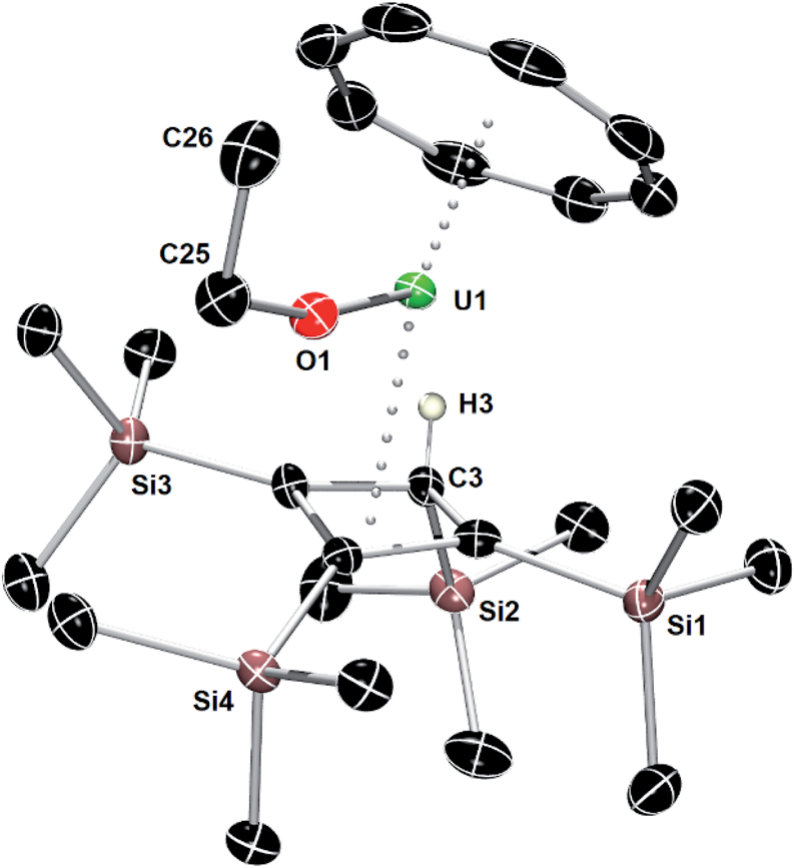
Thermal ellipsoid representation (50% probability) of the molecular structure of **5**. For clarity, only the allylic hydrogen atom is shown.

An X-ray diffraction study of single crystals obtained from an ether solution of the new compound confirmed the proposed molecular structure as [(η^3^-Cb′′′′H)U(η^8^-COT)(OEt)] (**5**) (Fig. 5, Table S1‡). The η^3^-bonding mode of the monoanionic Cb′′′′H ligand displays U–C distances of 2.617(5), 2.741(4) and 2.647(4) Å, with the distance to the centroid of the ligand being 2.490(3) Å. The deviation of SiMe_3_ substituent on the saturated carbon atom (C3) from the Cb′′′′H ring is 169.9(2)°, with the loss of ring planarity also reflected in the C1–C2–C3–C4 torsion angle of 9.7(3)°. The U–O distance of 2.063(3) Å is typical of a uranium(iv) alkoxide. The U–COT_cent_ distance of 1.987(7) Å is similar to that found for the U1 center of **1**.

Although the two-electron reductive cleavage of Et_2_O has been reported to occur during the reactions of UI_3_ with KCp^R^ (Cp^R^ = C_5_Me_5_, C_5_H_4_SiMe_3_, C_5_Me_4_H),^54^ to the best of our knowledge this is the first example of ether cleavage by uranium(iv) with no concurrent change in the metal oxidation state. Further insight into this process was obtained by reacting **1** and **2**/**3** with approximately 54 molar equivalents of Et_2_O in toluene-D_8_. The ^1^H NMR spectra of the two reactions (Fig. S48–S50, S52 and S53‡) showed the formation of **5** essentially in quantitative (>99%) yield. Significantly, the formation of ethene was confirmed by a singlet at 5.25 ppm (Fig. S51 and S53‡), and the ^29^Si{^1^H} NMR spectra are the same as that of isolated **5** (Fig. S51 and S54‡). Conducting the reaction of **2**/**3** with Et_2_O-D_10_ in benzene-D_6_ led to the formation of C_2_D_4_ (*δ* = 5.22 ppm), the deuterated ethoxide ligand and the allylic η^3^-Cb′′′′D ligand, shown by the similarity of the chemical shifts in the ^2^H NMR spectrum (Fig. S55 and S56‡) compared to those in the ^1^H NMR spectrum of the non-deuterated reaction (Fig. S52 and S53‡).

Furthermore, the lack of reaction between ether and the bulkier complex **4** suggests that coordination of the substrate to uranium is required in order for the cyclobutadienyl ligand to abstract a proton, pointing towards uranium–ligand cooperativity.

## Conclusions

In conclusion, the synthesis of the uranium(iv) sandwich complexes **1–4** containing cyclobutadienyl and cyclo-octatetraenyl ligands was accomplished, with hybrid uranocenes **3** and **4** being new members of the bis(annulene) family of actinide complexes. The tendency of the uranium centers in **1–3** to add a third ligand contrasts to the behavior of bis(cyclo-octatetraenyl) uranium compounds. The need for bulky substituents to prevent coordination of additional ligands was demonstrated with the isolation of **4**. A DFT study confirmed that formation of the THF adduct **2** is energetically favourable whereas formation of hypothetical **4**·THF has a negligible driving force. Analysis of the bonding in **3** and **4** revealed the presence of appreciable covalency, with an intriguing preference of the Cb′′′′ HOMOs to display more significant interactions with the uranium 6d orbitals than with the 5f orbitals, whereas the opposite is true for the uranium–COT interactions. The reaction of **3** with ether to give the allyl-ligated uranium(iv) ethoxide **5** implies that the Lewis acidity of uranium combined with the basicity of the cyclobutadienyl ligand may be of use in small-molecule activation, a concept which is under development in our laboratory.

## Conflicts of interest

There are no conflicts to declare.

## Supplementary Material

SC-012-D0SC05199C-s001

SC-012-D0SC05199C-s002
