# Supplementary material for: Synthesis, bonding properties and ether activation reactivity of cyclobutadienyl-ligated hybrid uranocenes
Source: Chem Sci. 2021 Jan 8;12(8):2948–54. doi: 10.1039/d0sc05199c (PMC8179396; doi:10.1039/d0sc05199c)
Supplement: SC-012-D0SC05199C-s001 [file SC-012-D0SC05199C-s001.pdf]

Electronic Supplementary Material (ESI) for Chemical Science.
This journal is © The Royal Society of Chemistry 2021

Synthesis, Bonding Properties and Ether Activation Reactivity of Cyclobutadienyl-Ligated Hybrid Uranocenes

Nikolaos Tsoureas,[a] Akseli Mansikkamäki,[b*] Richard A. Layfield[a*]

[a] Department of Chemistry, School of Life Sciences, University of Sussex, Brighton, BN1 9QJ, U.K.
[b] NMR Research Unit, University of Oulu, P. O. Box 8000, FI-90014, Finland

General Considerations: All manipulations were carried out using glovebox or standard Schlenk techniques. All glassware and cannulas were dried at 160°C overnight prior to use. Toluene and high-boiling hydrocarbons were dried over molten potassium, distilled under a nitrogen atmosphere and stored in ampoules over a potassium mirror. Low-boiling hydrocarbons were dried over sodium-potassium alloy, distilled under nitrogen and stored in ampoules over a potassium mirror. Et_2O was dried over sodium-potassium alloy, distilled under nitrogen and stored in ampoules over activated 4 Å molecular sieves. Deuterated toluene, THF and benzene were degassed by three freeze-thaw cycles, dried by refluxing over molten potassium for three days, vacuum distilled and stored in ampoules over 4 Å molecular sieves. Published procedures were used to synthesize $[U(BH_4)_4]$,[1] $[Na_2(0.75{\cdot}THF)Cb'''']$,[2] $Na[U(Cb'''')(BH_4)_3]$,[3] $K_2[COT]$[4] and $K_2[COT^{TIPS}]$[5] were prepared according to published procedures. ^1H, ^2H and $^{29}Si\{^1H\}$ NMR spectra were recorded on a Varian VNMR S400 spectrometer operating at 400 MHz (^1H) at 30°C unless otherwise stated. The ^1H spectra were referenced internally to the residual protic solvent. $^{29}Si\{^1H\}$ NMR spectra were referenced externally relative to $SiMe_4$. EI-MS data were recorded on a VG instrument at the University of Sussex. Elemental analyses were performed by Microanalytisches Labor Pascher, Germany.

Synthesis of 1, 2 and 3

A solution of $Na[U(Cb'''')(BH_4)_3]$ was freshly prepared in a Young's NMR tube using $U(BH_4)_4$ (72 mg, 0.24 mmol) and $[Na_2(0.75{\cdot}THF)Cb'''']$ (106.3 mg, 0.24 mmol) in a 3:1 mixture of THF-D_8 and toluene-D_8 (1 ml). The resulting brown-red solution of $Na[U(Cb'''')(BH_4)_3]$ was then placed in a vial with a stirrer bar and cooled at –35°C for 15 minutes in the glove box. Small portions of K_2COT (55.8 mg, 0.31 mmol) were added in small portions to the cold solution, with stirring. An immediate colour change to dark brown occurred. The reaction mixture was warmed to room temperature over *ca* 10 minutes and then filtered through a glass micro-fibre filter-pipette. The filter was washed with small amounts of dry THF. $^{29}Si\{^1H\}$ NMR spectroscopy showed complete consumption of $Na[U(Cb'''')(BH_4)_3]$. The brown solution was filtered through a filter-pipette to remove a grey solid and the volatiles were removed under vacuum. The dark brown-black residue was dried, extracted with hot *n*-heptane (2 × 10 mL, 1 × 5 ml) and filtered while hot. Slow and careful removal of *n*-heptane under vacuum led to the formation of crystals at the solvent interface, and removal of solvent continued until the remaining volume was about 1-2 mL.

After decanting the nascent heptane, washing the crystals with cold (–35°C) $SiMe_4$ (*ca* 1-2 mL) and drying, the material subsequently identified as $[(\eta^4\text{-}Cb'''')(\eta^8\text{-}COT)U(\mu{:}\eta^2{:}\eta^8\text{-}COT)U(THF)(\eta^4\text{-}Cb'''')]$ (**1**) was isolated (32 mg, 18% based on uranium).

The nascent heptane solution obtained from the recrystallization of **1** was slowly evaporated to a volume of 0.5-1.0 mL and stored at –35°C overnight, which resulted in the formation of block-like crystals of $[(\eta^4\text{-}Cb'''')U(THF)(\eta^8\text{-}COT)]$ (**2**). Removal of the *n*-heptane and drying in vacuum yielded **2** (20 mg, 11% with respect to uranium).

The $SiMe_4$ solution obtained from washing the crystals of **1** was allowed to evaporate to *ca* 0.1 mL. Storing the solution at –35°C overnight produced a co-crystal of **2**/**3** (11 mg, 8% based on uranium).

Isolation of 3

The THF-free compound $[(\eta^4\text{-}Cb'''')(\eta^8\text{-}COT)U]$ (**3**) was obtained from **1** and from **2** by preparing a solution of each compound in toluene-D_8 (10 mg in 0.4-0.5 ml) in a Young's NMR tube. Applying a vacuum of

approximately 10^{-3} mbar produced a dark brown film in each case. The NMR tubes were then connected to a turbo-vacuum pump and evacuated for six hours at 45°C at pressures ranging from 10^{-6} mbar to 9×10^{-7} mbar (final vacuum). The ^{1}H and ^{29}Si{^{1}H} NMR spectra of the resulting material in toluene-D_8 (Figures S18-S21) showed near-complete conversion to **3** in both cases. The two solutions were combined and, after removal of the solvent and thorough drying, the solid material was dissolved in the minimum volume of *n*-heptane. After filtration and slow evaporation to a volume of approximately 0.1 mL, crystals began to form. After storing the solution overnight at –35°C, the crystals were separated from the solvent and dried in vacuum, yielding **3** (15 mg, 82%).

Analytical data for [(η^4-Cb'''')(η^8-COT)U(μ:η^2:η^8-COT)U(THF)(η^4-Cb'''')] (1)
^{1}H NMR (δ/ppm, C_7D_8, 30°C): –37.51 (s, 16H, COT), –30.09 (broad s., FWHM = 313.54 Hz, 4H, THF), –14.85 (s, 72H, Si*Me₃*), –7.31 (broad s., FWHM = 67.30 Hz). ^{29}Si{^{1}H} NMR (δ/ppm, C_7D_8, 30°C): –204.28 (FWHM = 34.87 Hz).

^{1}H NMR (δ/ppm, C_7D_8, –50°C): –76.99 (broad s., FWHM = 425.15 Hz, 4H, THF), –50.96 (broad s., FWHM = 325.43 Hz, 16H, COT), –27.70 (broad s., FWHM = 623.35 Hz, 36H, SiMe_3), –19.54 (s., FWHM = 169.54 Hz, 4H, THF), –9.65 (broad s., FWHM = 630.53 Hz, 36H, SiMe_3). ^{29}Si{^{1}H} NMR (δ/ppm, C_7D_8, –50°C): –286.54 (FWHM = 615.58 Hz), –209.94 (FWHM = 566.16 Hz).

EI-MS: 683 = [U(COT)(Cb'''')], 512 = [U(COT)(Cb'''')+U(COT)]$^{2+}$, 439 = [U(COT)(Cb'''')+U(COT)-SiMe₃]$^{2+}$, 361 = [U(COT)+F], 342 = [U(COT)], 340 = (Cb'''') 73 = ($SiMe_3$) (the F comes from the $N(CF_2CF_3)_3$ calibrant of the instrument). Elemental analysis calculated for $C_{52}H_{96}OSi_8U_2$: C 43.43, H 6.73; Found: C 43.25, H 6.46.

Analytical data for [(η^4-Cb'''')U(THF)(η^8-COT)] (2)
^{1}H NMR (δ/ppm, C_7D_8, 30°C): –37.45 (s, 8H, COT), –17.14 (broad s., FWHM = 476.55 Hz, 4H, THF), –14.01 (s, 36H, SiMe_3), –4.04 (s, 4H, THF). ^{29}Si{^{1}H} NMR (δ/ppm, C_7D_8, 30°C): –200.22 (FWHM = 26.42 Hz).

^{1}H NMR (δ/ppm, C_7D_8, –50°C): –77.74 (broad s., FWHM = 591.79 Hz, 4H, THF), –50.65 (s, 8H, COT), –19.68 (broad s. FWHM = 367.29 Hz, 4H, THF), –9.69 (s. FWHM = 566.01 Hz, 36H, SiMe_3). ^{29}Si{^{1}H} NMR (δ/ppm, C_7D_8, –50°C): –213.08 (FWHM = 307.42 Hz).

EI-MS: 683 [U(COT)(Cb’’’’)], 512 [U(COT)(Cb’’’’)+U(COT)]$^{2+}$, 439 = [U(COT)(Cb’’’’)+U(COT)-SiMe₃]$^{2+}$, 361 = [U(COT)+F], 342 = [U(COT)], 340 = (Cb’’’’), 73 = ($SiMe_3$) (the F comes from the $N(CF_2CF_3)_3$ calibrant of the instrument). Elemental analysis calculated for $C_{28}H_{52}OSi_4U$: C 44.54, H 6.94; Found: C 44.53, H 6.63.

Analytical data for [(η^4-Cb'''')(η^8-COT)U] (3)
^{1}H NMR (δ/ppm, C_7D_8, 30°C): –37.90 (s, 8H, COT), –18.45 (s, 36H, SiMe_3). ^{29}Si{^{1}H} NMR (δ/ppm, C_7D_8, 30°C): –221.46 (FWHM = 22.29 Hz).

^{1}H NMR (δ/ppm, C_7D_8, –50°C): –52.22 (s., FWHM = 221.91 Hz, 8H, COT), –27.77 (s., FWHM = 109.85 Hz), 36H, SiMe_3). ^{29}Si{^{1}H} NMR (δ/ppm, C_7D_8, –50°C): –285.13 (FWHM = 615.58 Hz).

EI-MS: 683 = [U(COT)(Cb'''')], 512 = [U(COT)(Cb''''’)+U(COT)]$^{2+}$, 439 = [U(COT)(Cb'''')+U(COT)-SiMe₃]$^{2+}$, 361 = [U(COT)+F], 342 = [U(COT)], 340 = (Cb''''), 73 = ($SiMe_3$) (the F comes from the $N(CF_2CF_3)_3$ calibrant of the instrument). Elemental analysis calculated for $C_{24}H_{44}Si_4U$: C 42.21, H 6.49; Found: C 42.51, H 6.51.

Analytical data for the co-crystal of [(η^4-Cb'''')U(η^8-COT)]/[(η^4-Cb'''')U(THF)(η^8-COT)] (2/3). ^{1}H NMR (δ/ppm, C_7D_8): –37.76 (s, 8H, COT), –17.67 (s, 36H, SiMe_3). Signals for THF were not located. ^{29}Si{^{1}H} NMR (δ/ppm, C_7D_8): –217.70.

EI-MS: 683 = [(Cb'''')(COT)U], 446 = [U(COT)$_2$], 342 = [U(COT)]. Elemental analysis calculated for $C_{52}H_{96}OSi_8U_2 \cdot SiMe_4$, C 44.07, H 7.13; found, C 43.89, H 6.94.

Synthesis of [(η^4-Cb'''')U(η^8-COT^{TIPS})] (4)
This was made in a similar manner to that described for 1-3, using U(BH$_4$)$_4$ (30 mg, 0.1 mmol), [Na$_2$(0.75·THF)Cb''''] (44.3 mg, 0.1 mmol and K$_2$COT^{TIPS} (63 mg, 0.13 mmol). After filtration of the brown reaction mixture and removal of the volatiles, the brown residue was taken up in SiMe$_4$ (3 mL), filtered through a pipette containing Celite, and the solution was allowed to slowly evaporate in a glove box to a volume of about 1 m. Storing the solution at –35°C) produced brown crystals of 4 (12 mg, 12%, the low isolated yield being due to the high solubility of the compound).

Analytical data for 4
^{1}H NMR (δ/ppm, C$_7$D$_8$): –156.23 (br. s, 2H, C$_8$*H*$_6$(TIPS)$_2$), –99.09 (br. s, 2H, C$_8$*H*$_6$(TIPS)$_2$), –9.37 (s, 36H, Si*Me*$_3$), –4.24 (d, $^3J_{HH}$ = 4.53 Hz, 18H, Si(CH(C*H*$_3$)$_2$), –0.135 (d, $^3J_{HH}$ = 4.36 Hz, 18H, Si(CH(C*H*$_3$)$_2$), 2.23 (br. s, 6H, Si(C*H*(CH$_3$)$_2$, partially overlapped with residual protio-toluene (CH$_3$)), 103.8 (br. s, 2H, C$_8$*H*$_6$(TIPS)$_2$). ^{29}Si{^{1}H} NMR (δ/ppm, C$_7$D$_8$): –139.63, –33.80.

EI-MS: 1071 = [U(COT^{TIPS})$_2$], 995 = [(Cb'''')(COT^{TIPS})U] = M, 921 = M – SiMe$_3$), 654 = M – Cb'''', 416 = (COT^{TIPS}), 373 = COT^{TIPS} – ^{i}Pr, 157 = Si^{i}Pr$_3$, 115 = HSi^{i}Pr$_2$, 73 SiMe$_3$. Elemental analysis calculated for $C_{42}H_{84}Si_6U$, C 50.67, H 8.50; found, C 49.97, H 7.92.

Synthesis of [(η^3-Cb''''H)U(η^8-COT)(OEt)] (5)
In a typical synthesis, and mixture of **1**, **2** and **3** was formed in situ from a reaction involving U(BH$_4$)$_4$ (30 mg, 0.1 mmol), [Na$_2$(0.75·THF)Cb''''] (44.3 mg, 0.1 mmol) and K$_2$COT (23 mg, 0.13 mmol) in THF/toluene, as described above. After removal of the solvent, the residue was dissolved in a 1:2 mixture of toluene and Et$_2$O (4 ml, n-hexane/Et$_2$O can also be used) and the brown solution was filtered through a micro-glass fibre filter pipette. The brown solution was stirred overnight upon which time the colour changed to dark red. This red solution was filtered again, the solvent was removed and the residue was dried, extracted into benzene C$_6$H$_6$ and filtered as before. Compound **5** (44 mg, 60%) was isolated as a red microcrystalline solid after lyophilisation of the solution. Crystals suitable for single-crystal X-ray diffraction were deposited during slow evaporation of an ether solution of **5** in a vial at room temperature. Over periods of 7-10 days, solid samples of compound **5** change colour from red to grey even if stored in a glove box freezer at –35°C.

Alternatively, compound **5** can be synthesized by adding Et$_2$O (5 µl, 0.19 mmol) to a solution of **2**/**3** (5 mg) in toluene-D8 (0.5 ml) in a Young's NMR tube. The sample was immediately sealed. Upon stirring overnight at room temperature, the brown solution turned red and the formation of **5** in almost quantitative spectroscopic yield was observed with concurrent formation of C_2H_4.

Analytical data for 5
^{1}H NMR (δ/ppm, C$_6$D$_6$): –32.54 (s, 8H, COT), –21.37 (s, 18H, Si*Me*$_3$), –3.26 (s, 9H, Si*Me*$_3$), 16.87 (s, 9H, Si*Me*$_3$), 25.97 (s, 3H, OCH$_2$C*H*$_3$), 45.52 (s, 1H, CBD'), 94.77 (s, 2H, OC*H*$_2$CH$_3$). ^{29}Si{^{1}H} NMR (δ/ppm, C$_6$D$_6$): –255.64, –64.11, 37.87. EI-MS: 729 = M, 387 = (M – Cb''''), 342 = U(COT), 73 = SiMe$_3$. Elemental analysis calculated for $C_{26}H_{50}OSi_4U$, C 42.83, H 6.91; found, C 42.38, H 6.58.

Synthesis of 5 using Et$_2$O-D$_{10}$. In a similar manner to the non-deuterated reaction, a sample of **2**/**3** containing 10% of **1** (10 mg) was placed in a Young's NMR tube and dissolved in C$_6$D$_6$ (0.5 ml), followed by the addition of Et$_2$O-D$_{10}$ (30 ml). After a few minutes a colour change to dark red was observed and the NMR spectra recorded. Full consumption of **2**/**3** was observed after *ca* 10 hrs at room temperature.

X-ray Crystallography

All data collections were carried out on a Rigaku Gemini Ultra diffractometer equipped with an EOS-CCD detector and a four-circle kappa goniometer performing ω scans to fill the Ewald sphere at 100 K, using Cu/Kα radiation. Crystals were mounted on MiTigen loops from pump oil kept over molecular sieves in a glove box. Data for **1** were collected to 0.81 Å resolution, for **2**, **3**, **2/3** (co-crystal) and **4** to 0.82 Å resolution, and for **5** to 0.83 Å resolution.

In the case of **2/3**, the structure was refined as an enantiomeric twin (65:35, BASF = 0.3488). There is significant disorder in the structure: specifically, some of the $SiMe_3$ carbons are split over two positions and, although the disorder can be modelled, the refinement is not stable unless an EADP constraint is used in some cases. Furthermore, there are two highly disordered solvent molecules of crystallisation ($SiMe_4$) located in special positions, which that proved difficult to model and achieve a stable and converging refinement. As a result, the Si–C bonds were constrained using the DFIX command and the same was decided for some of the distances between carbon atoms using the DANG command. Finally, the $SiMe_4$ solvent crystallisation molecules were treated as rigid bodies using the RIGU command.

In the case of **3**, the $SiMe_3$ groups are disordered over two positions. Some of the methyl carbon atoms had to be refined isotropically using the ISOR command. Similarly, the coordinated THF was modelled as disordered between two positions and SADI restraints were used to achieve this. Some atoms on the disordered THF were refined isotropically using the ISOR command. Finally, an analytical correction based on crystal faces was applied to **3** and **2**.[6]

In the case of **4**, the collection was performed by placing the detector at a 50 mm distance from the crystal. Two molecules were found in the asymmetric unit and one of them showed significant disorder both on the $Si^{i}Pr_3$ groups and the Cb'''' ligand, with the latter being disordered over two positions with some of the $SiMe_3$ groups of these two parts sharing CH_3 substituents (use of EXYZ and EADP to model). The modelling of the disorder was also achieved by using SADI and RIGU restraints. Furthermore, the model was refined as a merohedral twin (twin law: -0.004 0 1.004/ 0 -1 0/ 0.996 0 0.004 as determined with PLATON TWINROTMAT) with a BASF of *ca* 0.07 which significantly reduced the *R*1 and *wR*2.

Table S1. Crystal data and structure refinement for **1-5**.

	**1**	**2**	**3**	**2/3**	**4**	**5**
CCDC code	2026517	2049727	2049726	2026518	2026519	2026520
Colour, habit	brown, plate	intense brown, block	dark brown, plate	brown, block	dark brown, plate	light red, plate
Size/mm	0.02 × 0.08 × 0.2	0.180 × 0.095 × 0.082	0.282 × 0.177 × 0.023	0.1 × 0.2 × 0.4	0.08 × 0.1 × 0.2	0.01 × 0.1 × 0.2
Empirical formula	$C_{52}H_{96}OSi_8U_2$	$C_{58}H_{52}OSi_4U$	$C_{24}H_{44}Si_4U$	$C_{58.3}H_{113.5}OSi_9U_2$	$C_{84}H_{168}Si_{12}U_2$	$C_{26}H_{50}Si_4OU$
FW	1438.06	755.08	682.98	1559.93	1991.31	729.05
Crystal system	orthorhombic	orthorhombic	tetragonal	tetragonal	monoclinic	monoclinic
Space group	$P2_12_12_1$	$P2_12_12_1$	$I4_1/a$	P–4	$P2_1/c$	$P2_1/n$
a/Å	10.5444(3)	11.4039(3)	31.8409(2)	20.3708(2)	26.7057(3)	17.5935(3)
b/Å	18.4631(4)	16.9770(4)	31.8409(2)	20.3708(2)	15.99077(15)	9.9770(2)
c/Å	31.5083(7)	17.0354(4)	11.02340(10)	16.6734(3)	26.5612(4)	18.6747(4)
α/°	90	90	90	90	90	90
β/°	90	90	90	90	117.3495(17)	110.001(2)
γ/°	90	90	90	90	90	90
V/Å^3	6134.1(3)	3298.14(14)	11176.00(17)	6919.0(2)	10074.9(2)	3080.27(11)
Z	4	4	16	4	4	4
μ/mm^{-1}	16.485	15.371	18.048	14.820	10.613	16.434
T/K	100	100	100	100	100	100
θ min/max	3.688/71.580	3.675/70.749	3.927/71.327	3.425/70.964	3.378/70.246	2.980/ 67.759
Completeness/%	99.87	99.6 to 70.749	99.5 to 71.327	99.9	99.9	98.6
Reflections Total/Independent	11753/11145	6302/6162	5417/4857	13128/12661	19365/17014	5495/5317
R_{int}	0.0515	0.0963	0.0725	0.0510	0.0648	0.0406
Final $R1$, $wR2$	0.0287, 0.0637	0.0273/0.0647	0.0266/0.0617	0.0658, 0.1616	0.0500, 0.1306	0.0358, 0.0967
GOOF	1.029	1.023	1.026	1.076	1.061	1.076
Flack	0.039(4)	0.030(5)	-	0.000(4)	-	-
Largest peak, hole / e.Å^{-3}	1.2, –1.1	0.9 and –1.2	1.3 and –1.3	5.6, –1.8	5.2, –4.1	3.4, –2.0
ρ_{calc}/g cm^{-3}	1.557	1.521	1.624	1.498	1.313	1.572

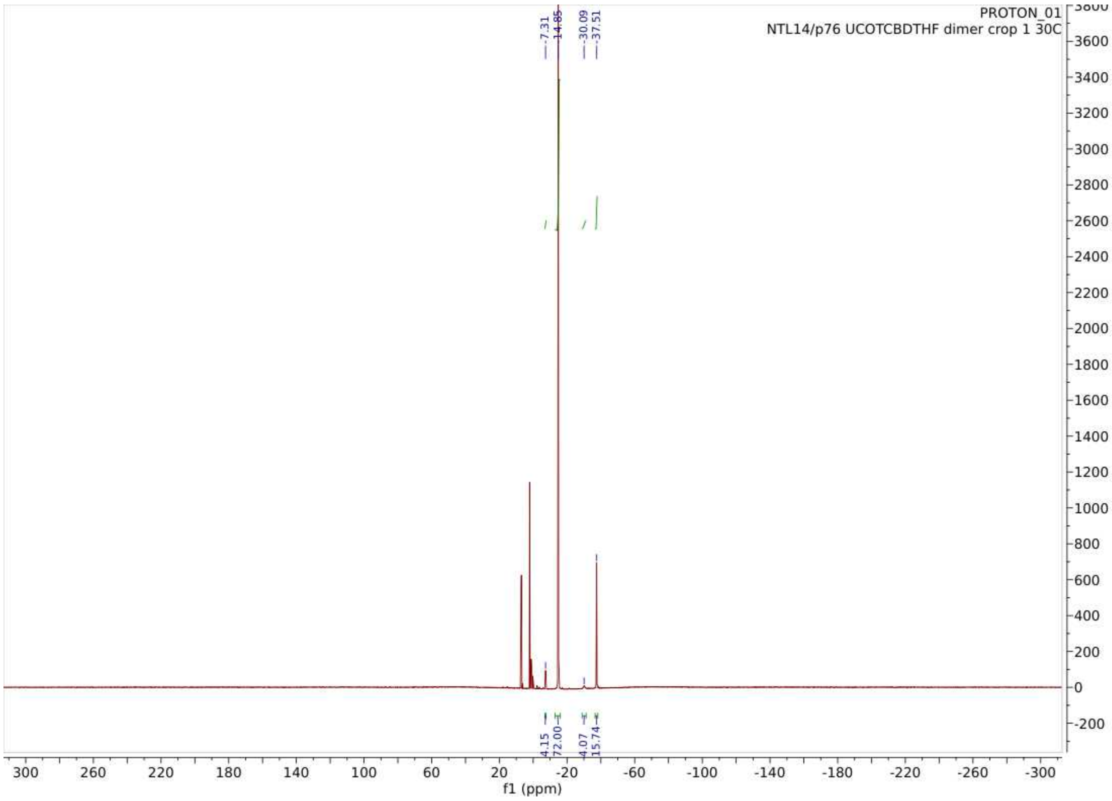

Figure S1. ^{1}H NMR spectrum of **1** in toluene-D$_8$ at 30°C.

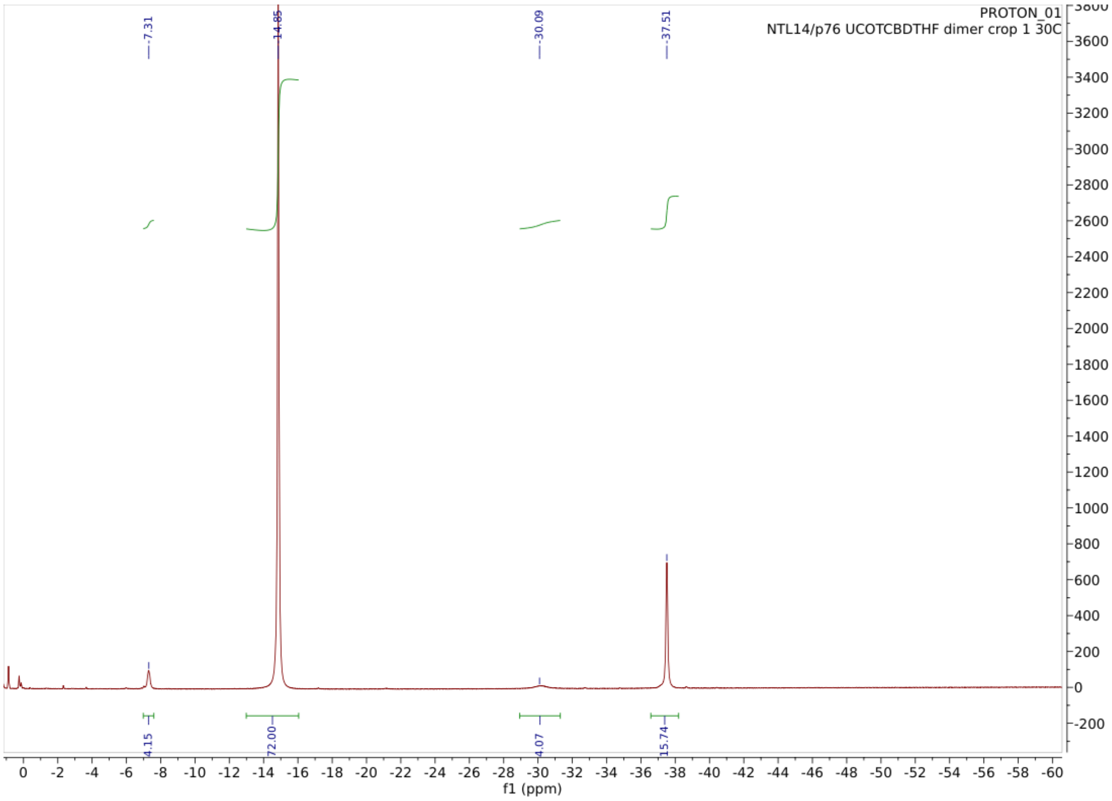

Figure S2. Expanded ^{1}H NMR spectrum of **1** in toluene-D_8 at 30°C.

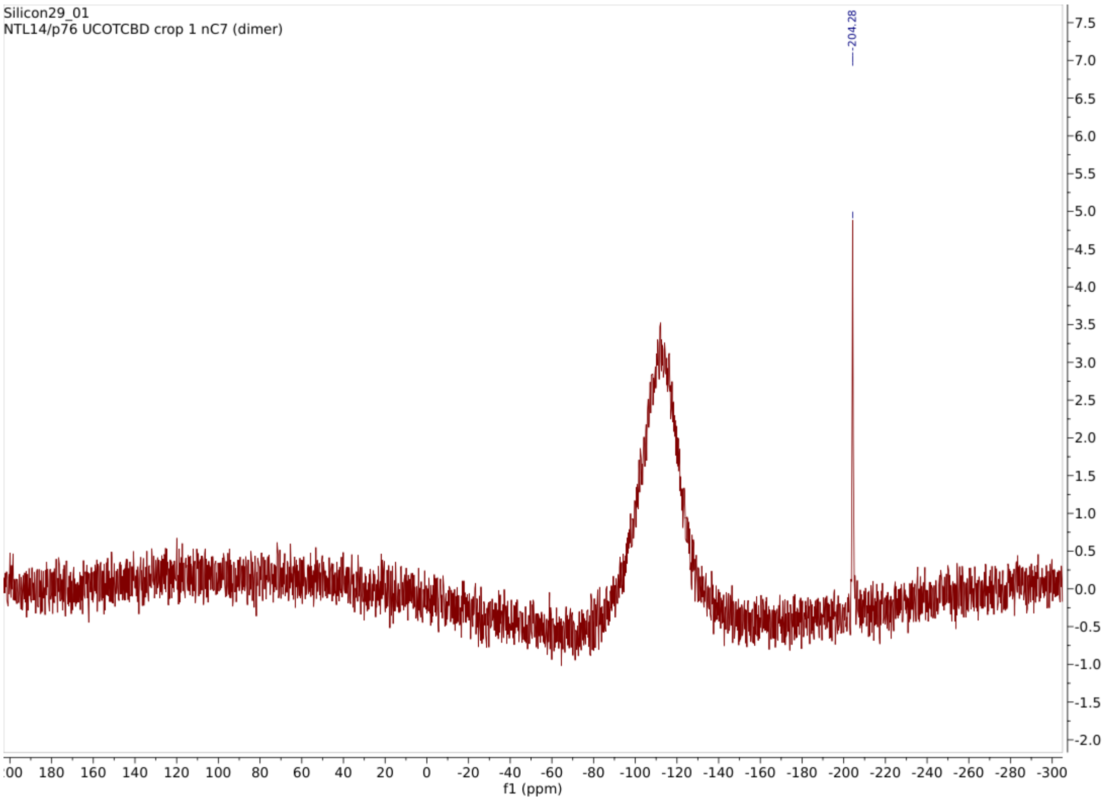

Figure S3. $^{29}Si\{^{1}H\}$ NMR spectrum of **1** in toluene-D_8 at 30°C.

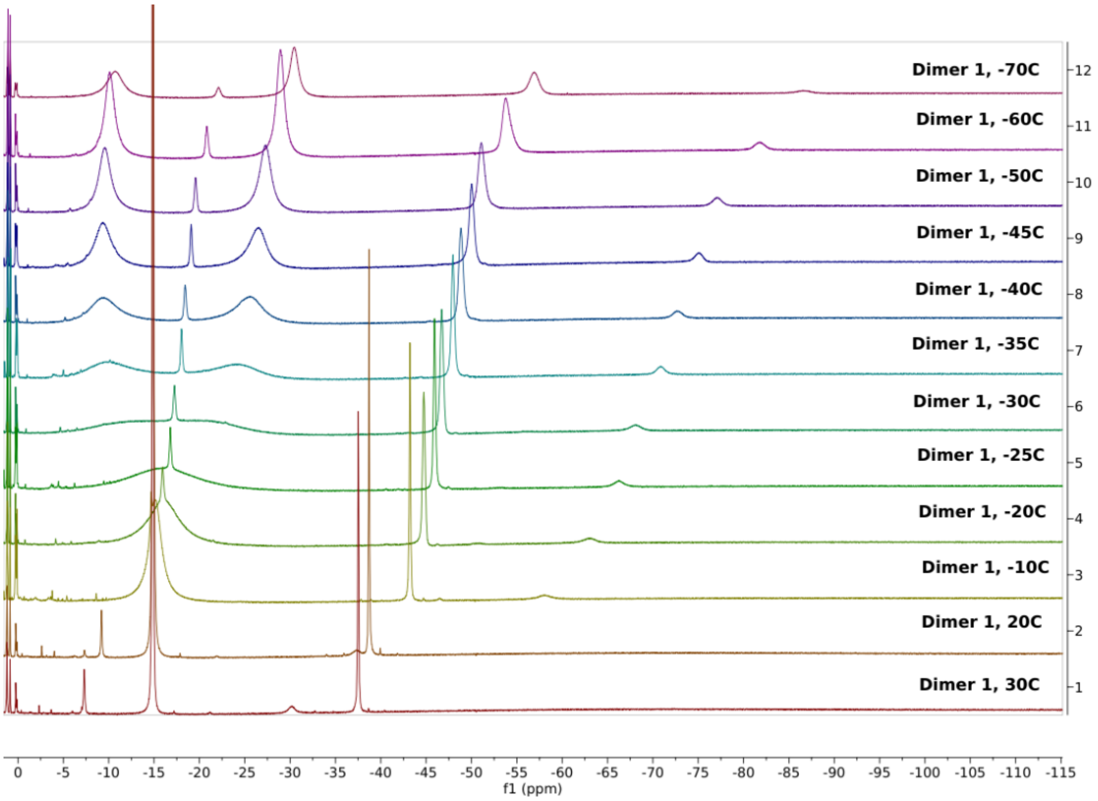

Figure S4. Variable-temperature ^{1}H NMR spectra of **1** in toluene-D_8.

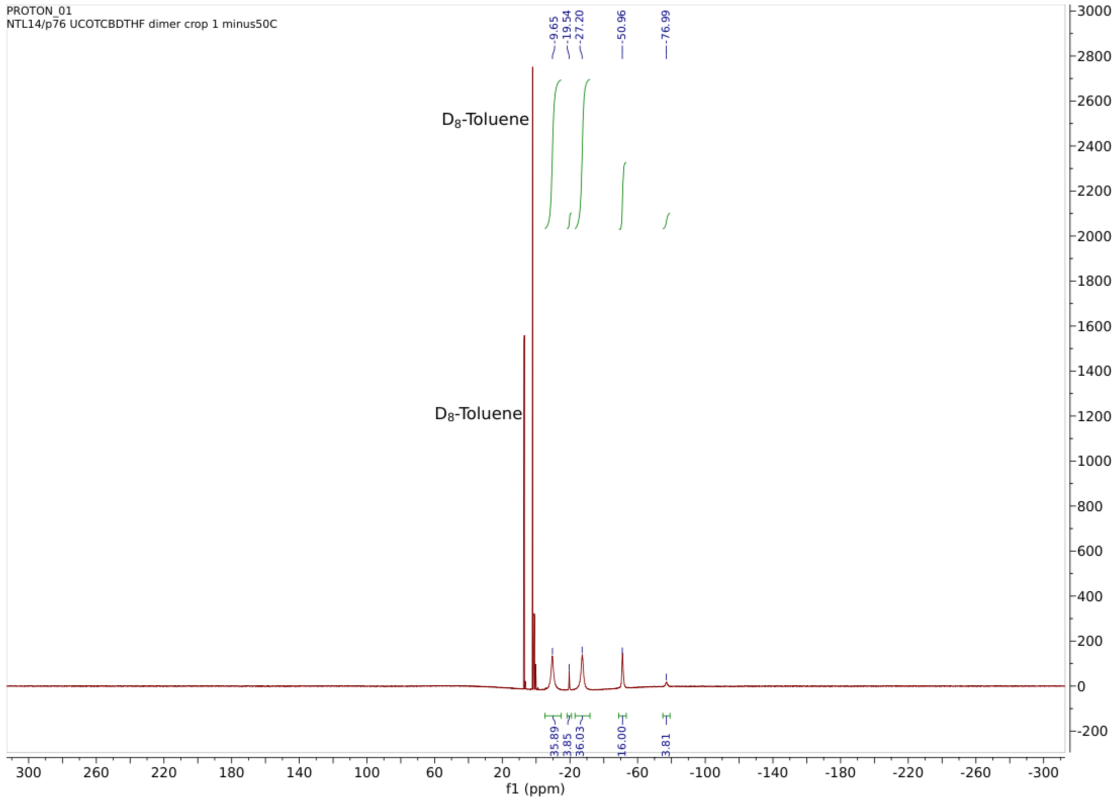

Figure S5. ^{1}H NMR spectrum of **1** in toluene-D_8 at –50 °C.

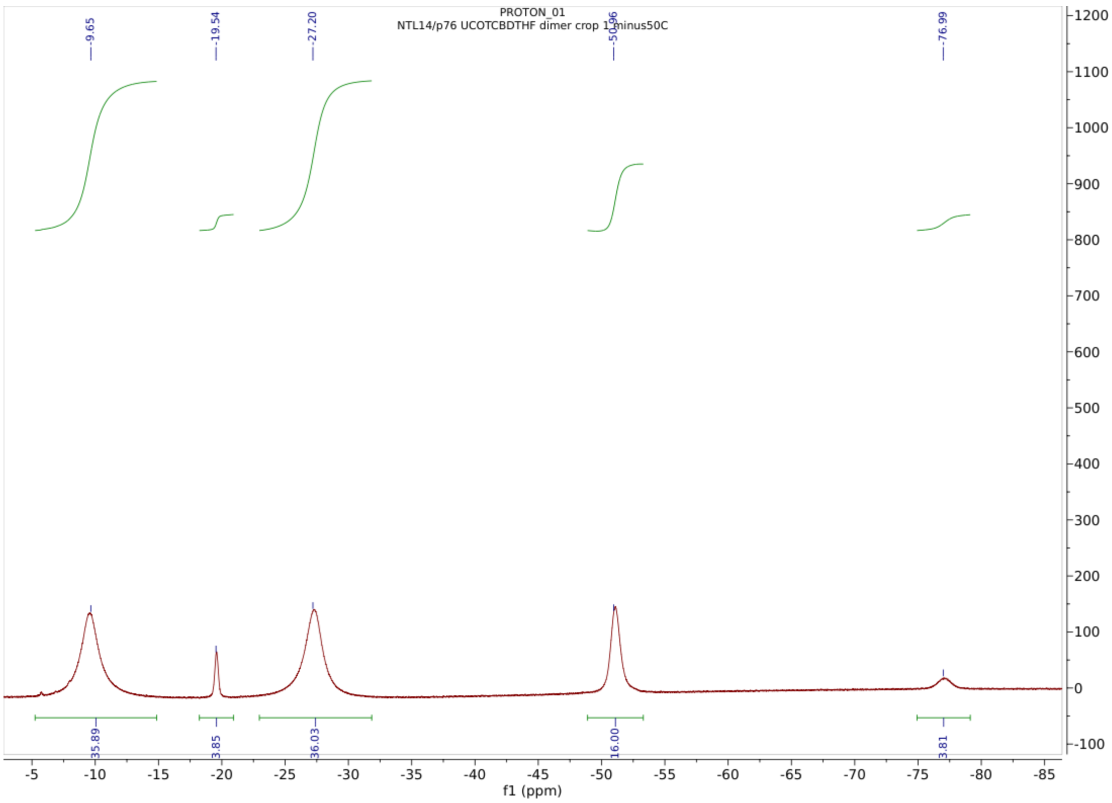

Figure S6. Expanded ^{1}H NMR spectrum of **1** in toluene-D$_8$ at –50 °C.

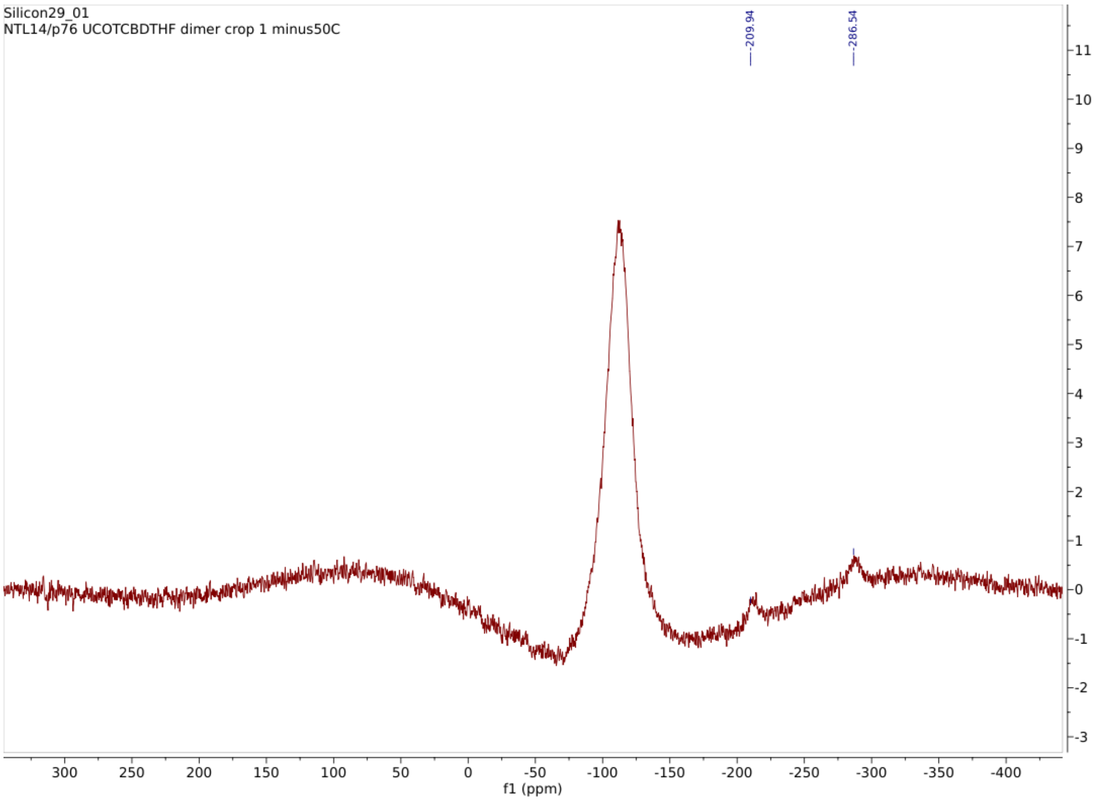

Figure S7. $^{29}Si\{^{1}H\}$ NMR spectrum of **1** in toluene-D_8 at –50°C.

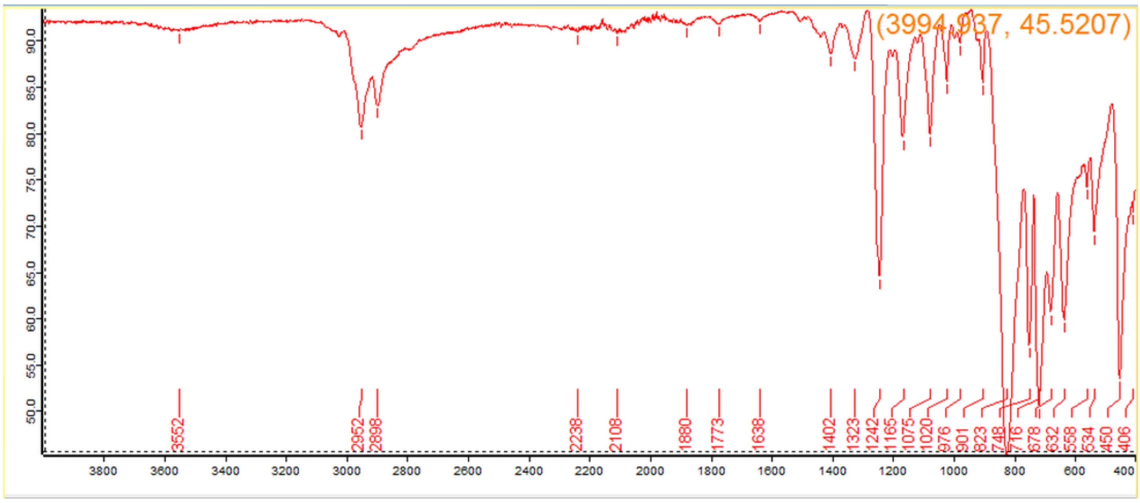

Figure S8. IR spectrum of **1**.

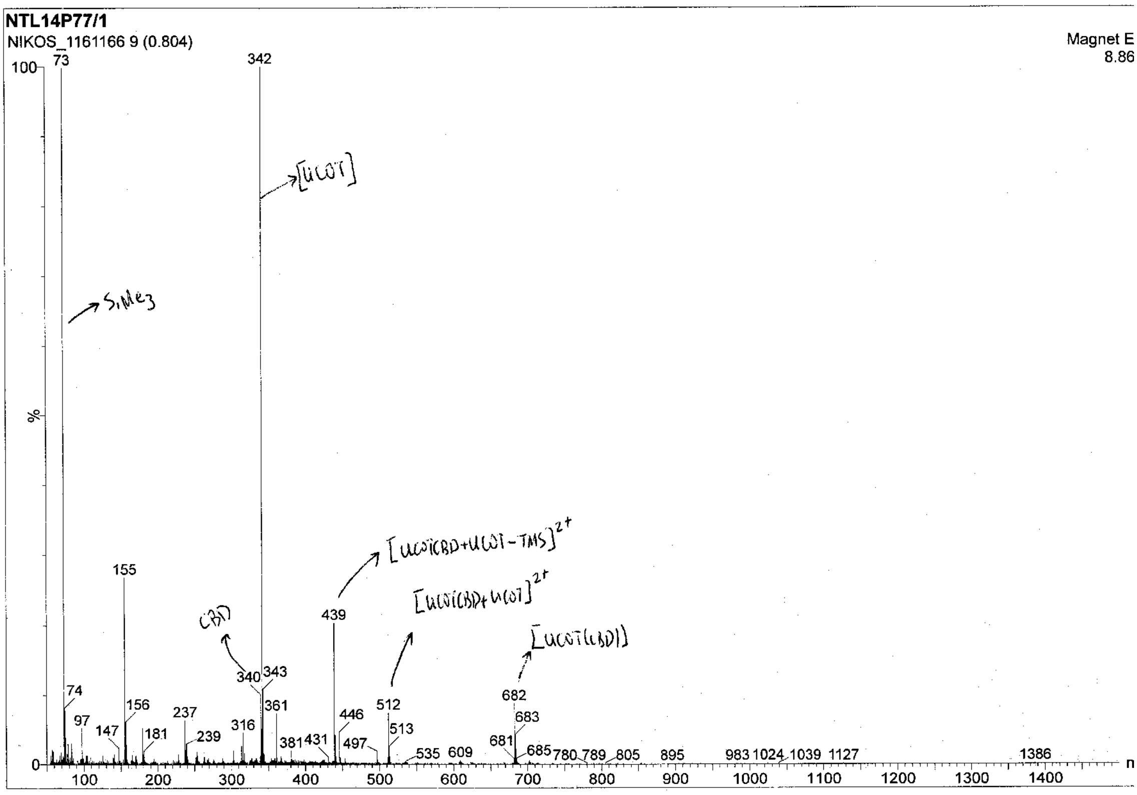

Figure S9. EI-MS spectrum of **1**.

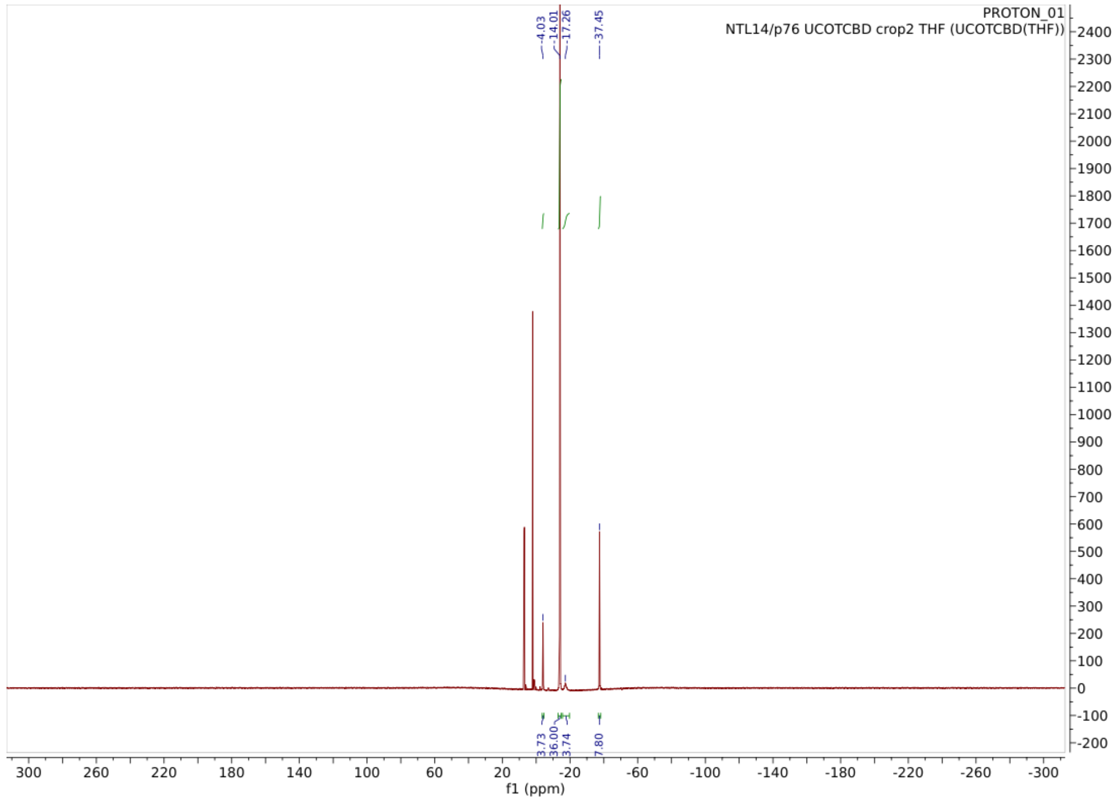

Figure S10. ^{1}H NMR spectrum of **2** in toluene-D_8 at 30°C.

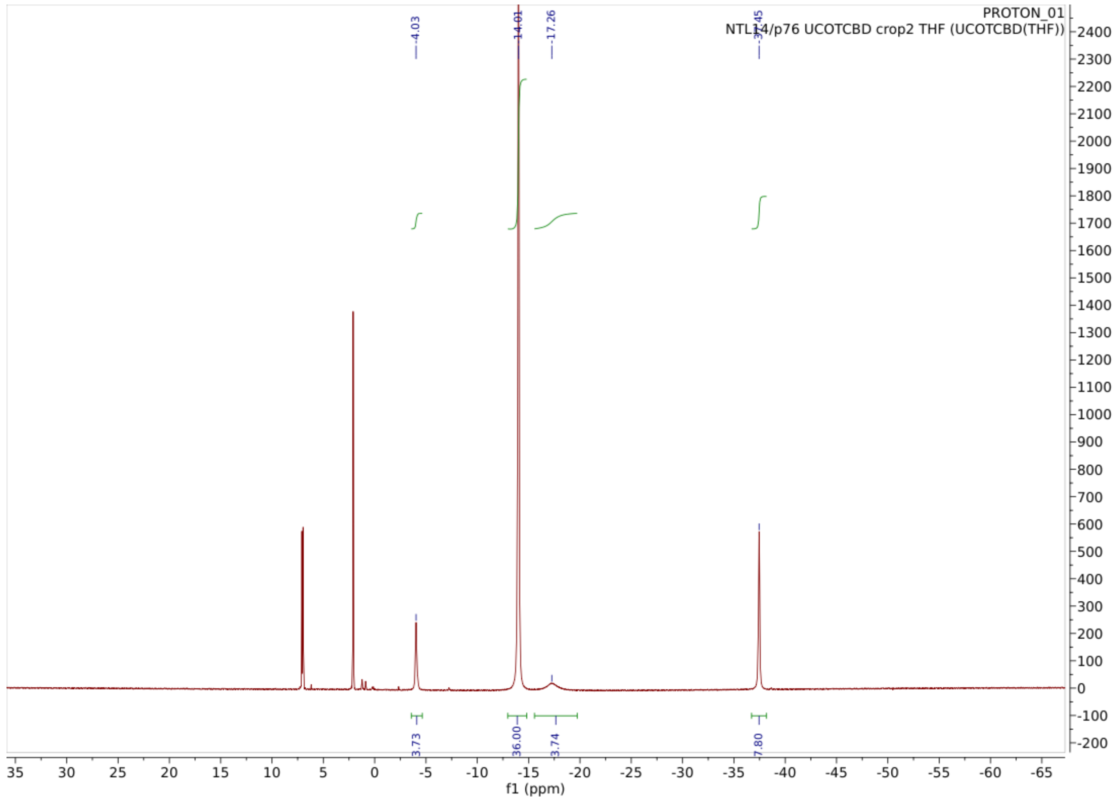

Figure S11. Expanded ^{1}H NMR spectrum of **2** in toluene-D_8 at 30°C.

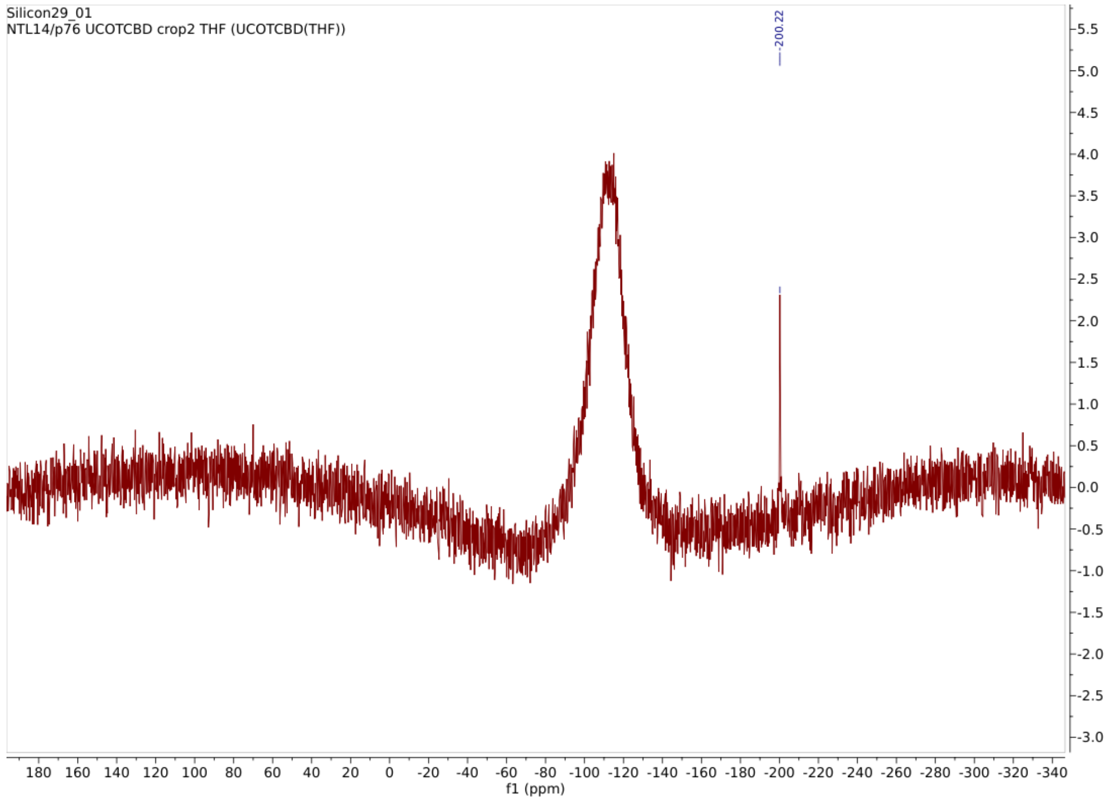

Figure S12. $^{29}Si\{^{1}H\}$ NMR spectrum of **2** in toluene-D_8 at 30°C.

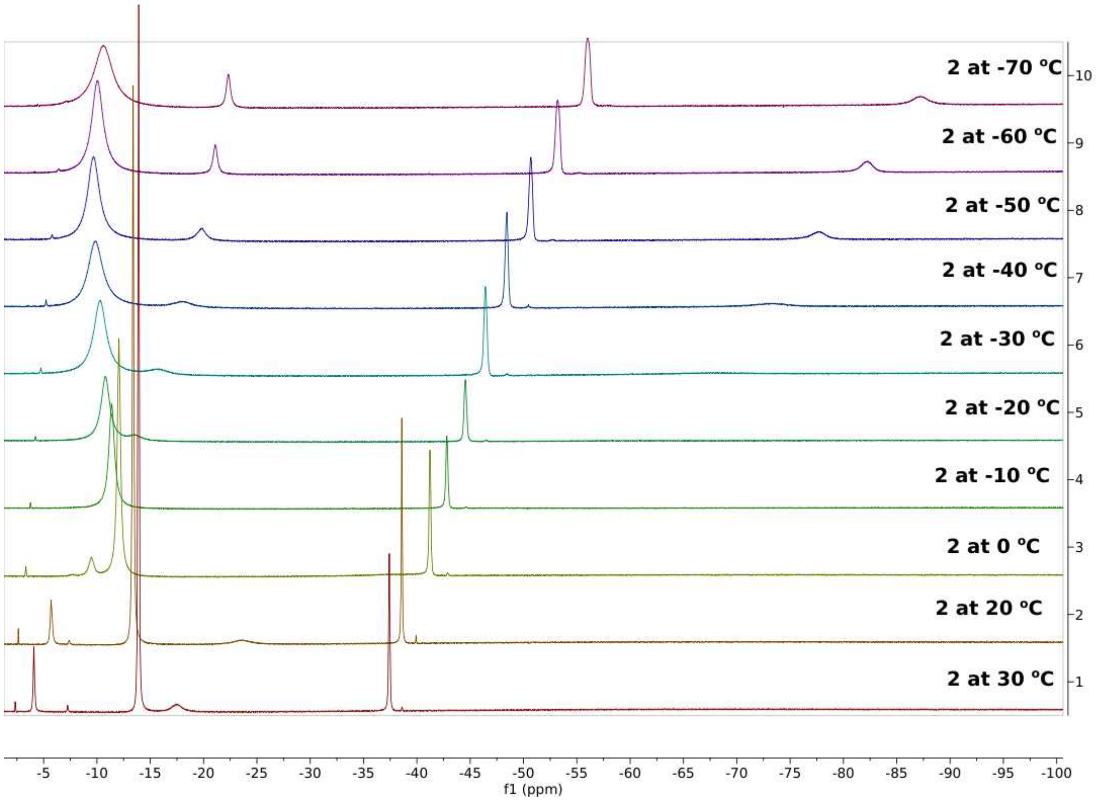

Figure S13. Variable-temperature ^{1}H NMR spectra of **2** in toluene-D_8.

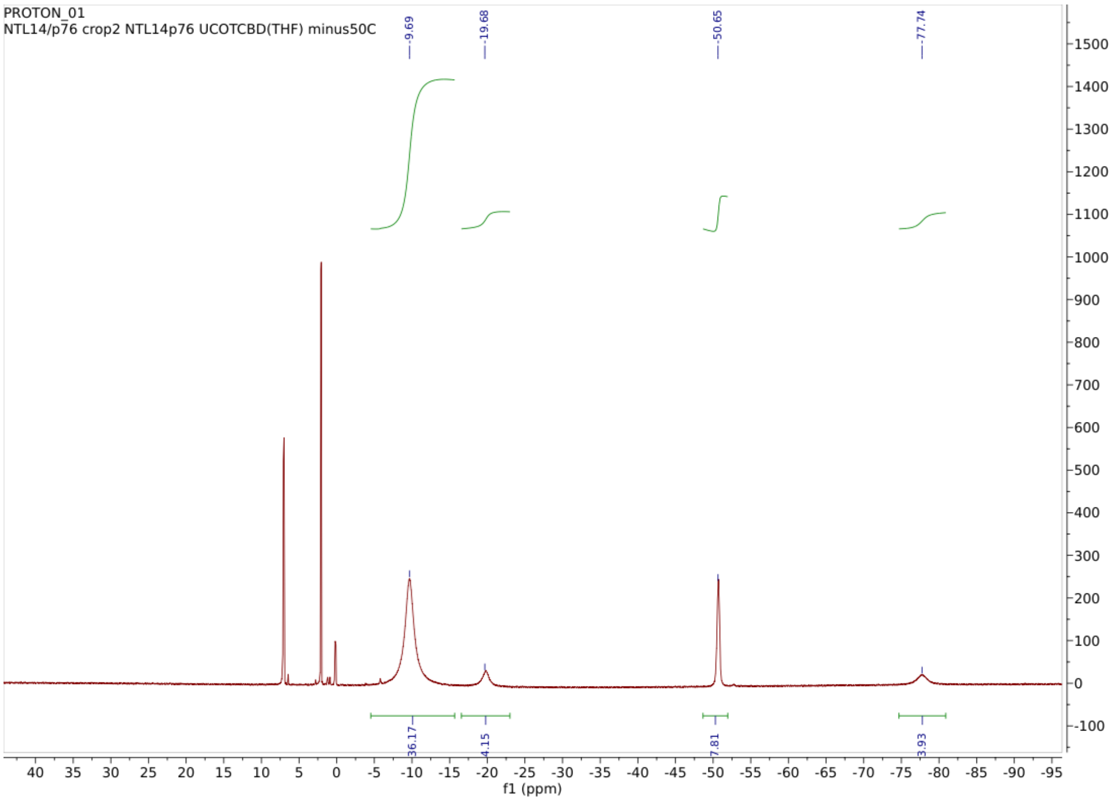

Figure S14. ^{1}H NMR spectrum of **2** in toluene-D_8 at –50 °C.

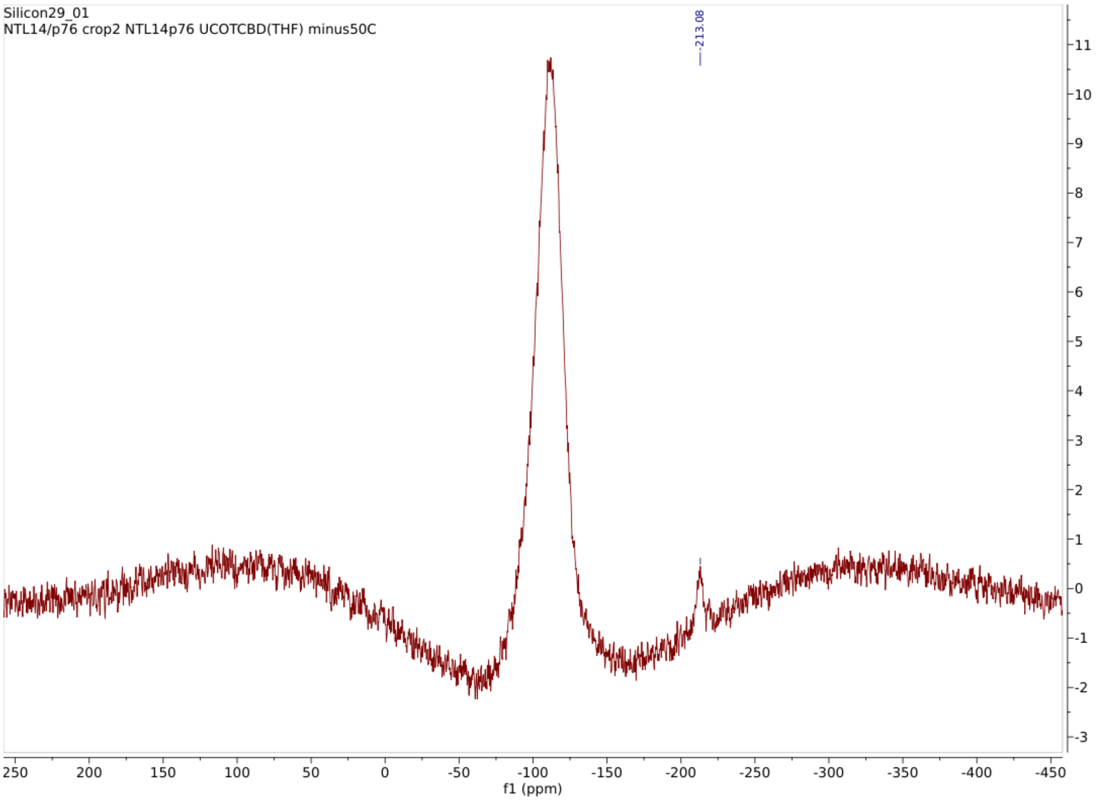

Figure S15. $^{29}Si\{^{1}H\}$ NMR spectrum of **2** in toluene-D_8 at –50 °C.

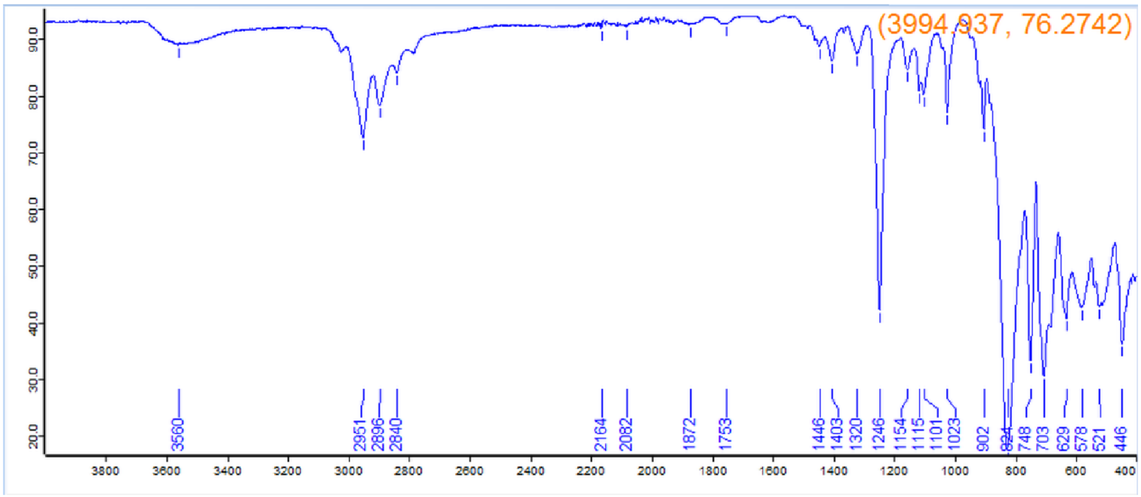

Figure S16. IR spectrum of **2**.

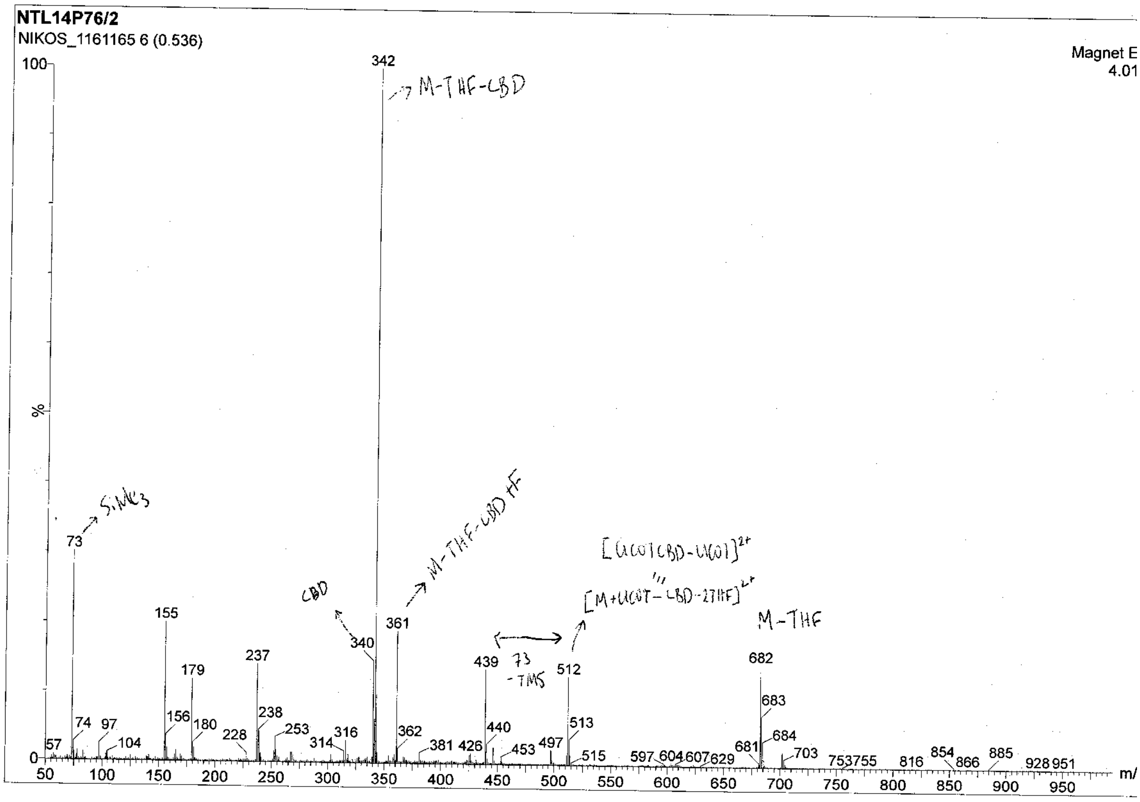

Figure S17. EI-MS spectrum of **2**.

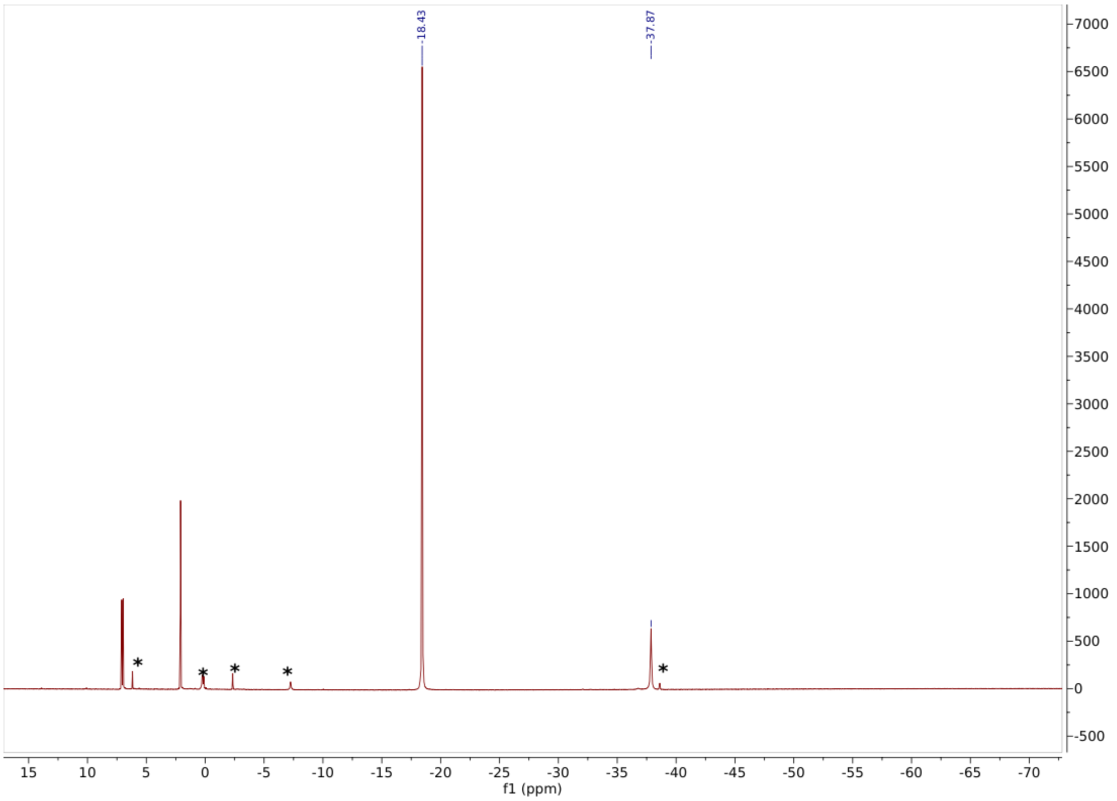

Figure S18. ^{1}H NMR spectrum in toluene-D_8 of crude **3** obtained after de-solvation of **1** (* = minor impurities).

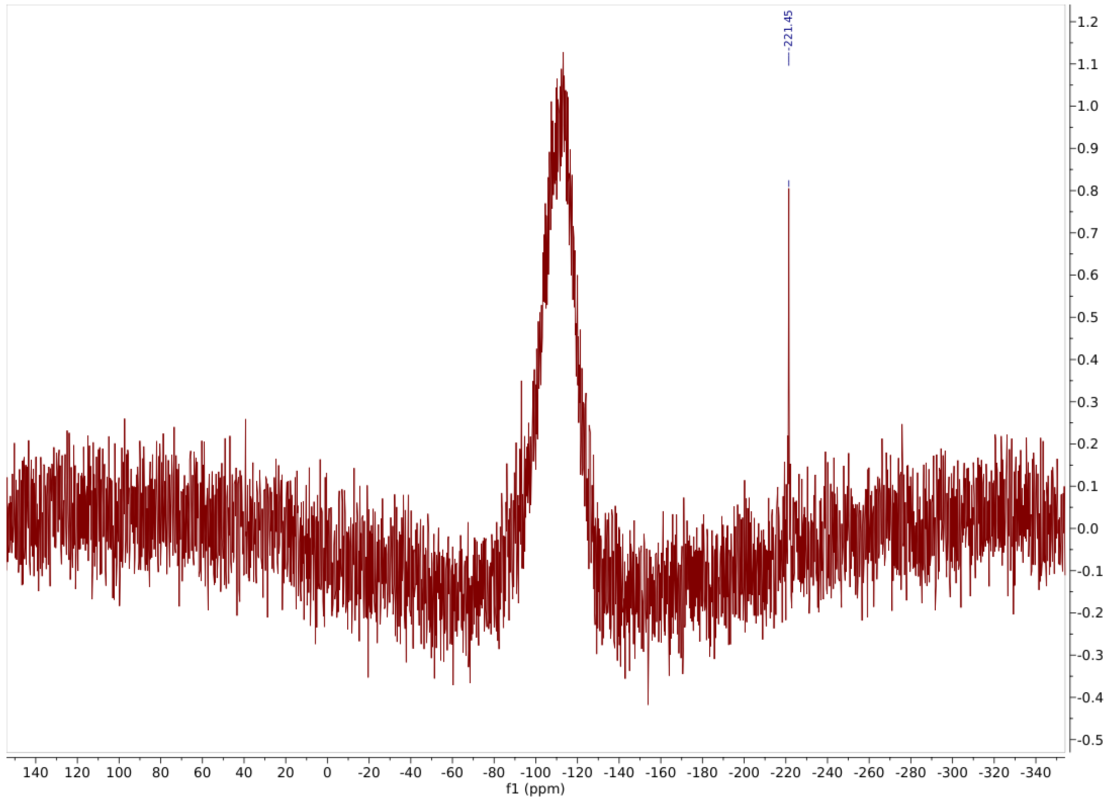

Figure S19. $^{29}Si\{^1H\}$ NMR spectrum in toluene-D$_8$ of crude **3** obtained after de-solvation of **1** (* = minor impurities).

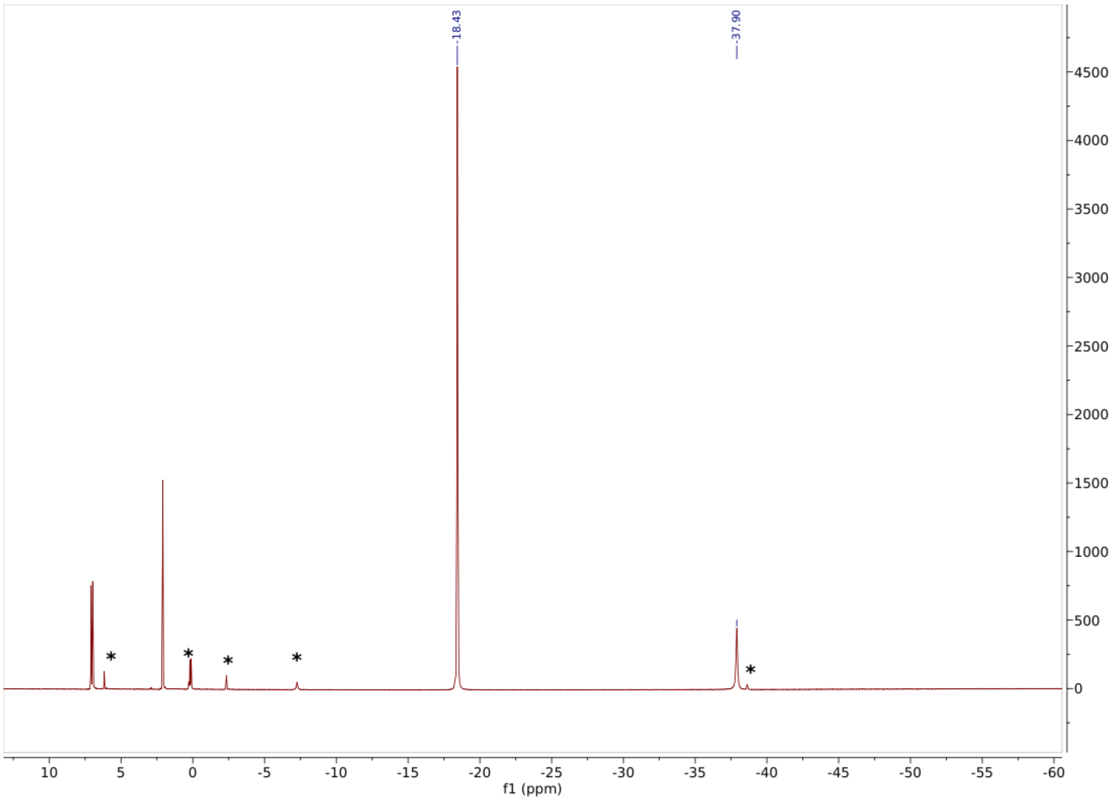

Figure S20. ^{1}H NMR spectrum in toluene-D_8 of crude **3** obtained after de-solvation of **2** (* = minor impurities).

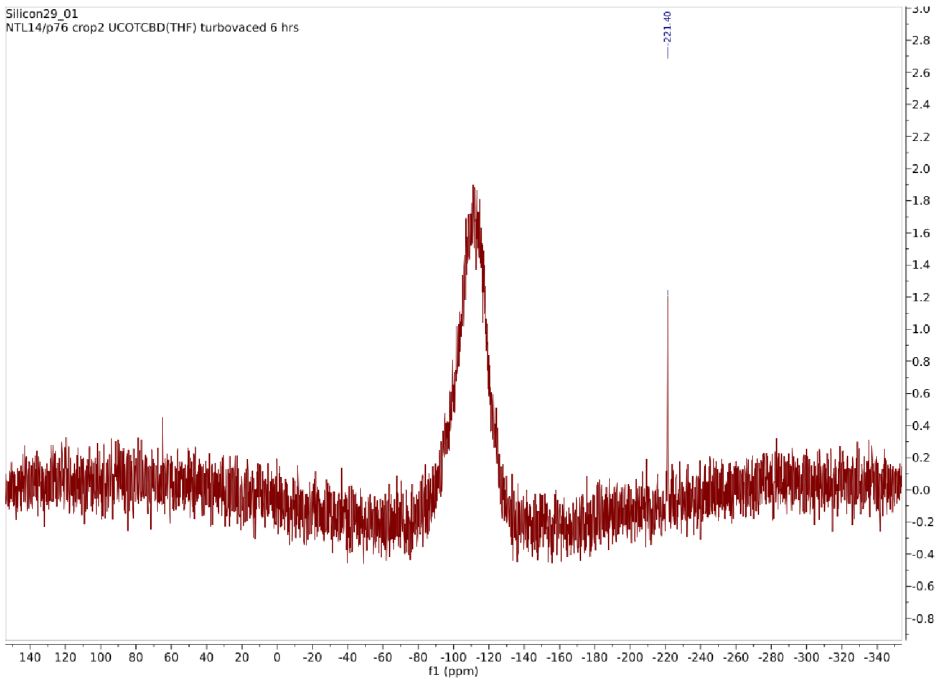

Figure S21. $^{29}Si\{^1H\}$ NMR spectrum in toluene-D_8 of crude **3** obtained after de-solvation of **2** (* = minor impurities).

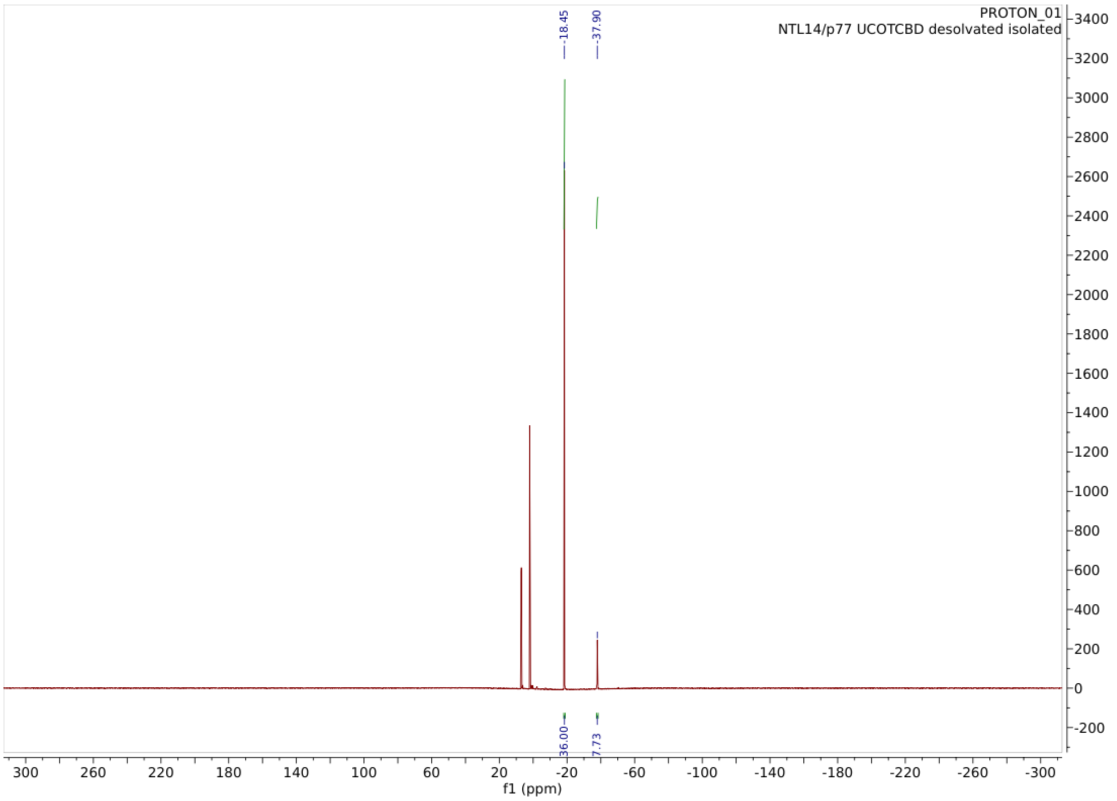

Figure S22. ^{1}H NMR spectrum of **3** in toluene-D_8 at 30°C.

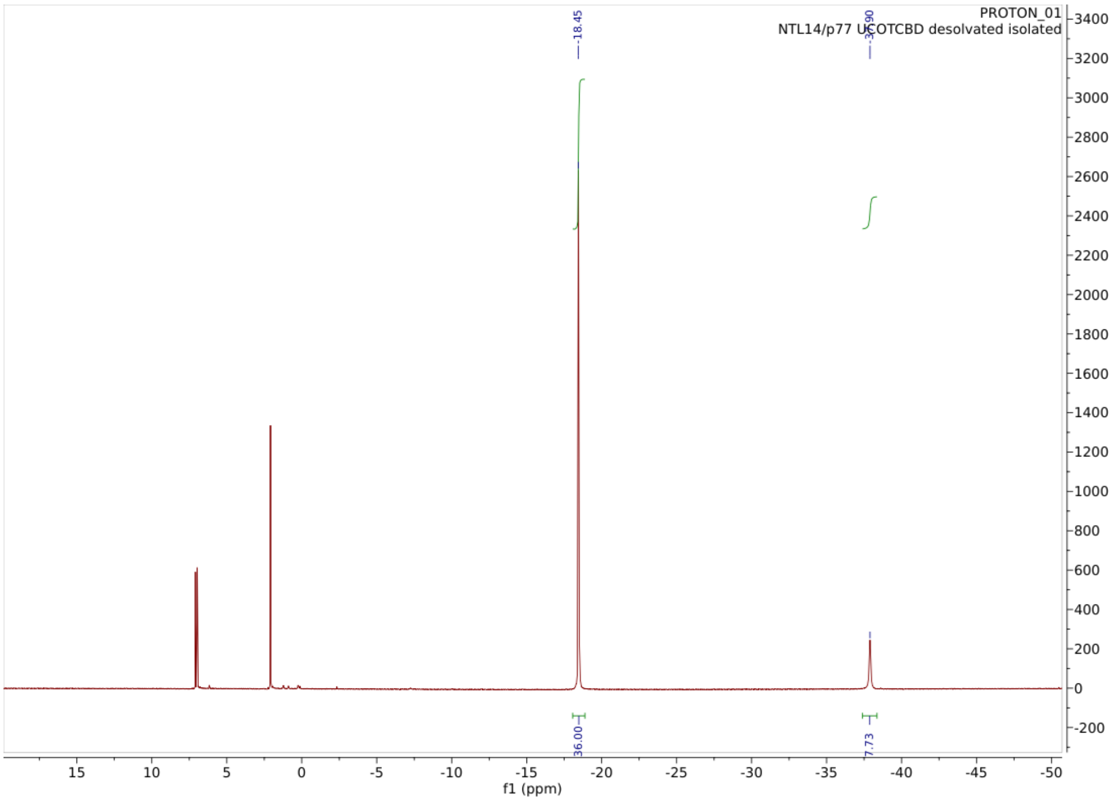

Figure S23. Expanded ^{1}H NMR spectrum of **3** in toluene-D_8 at 30°C.

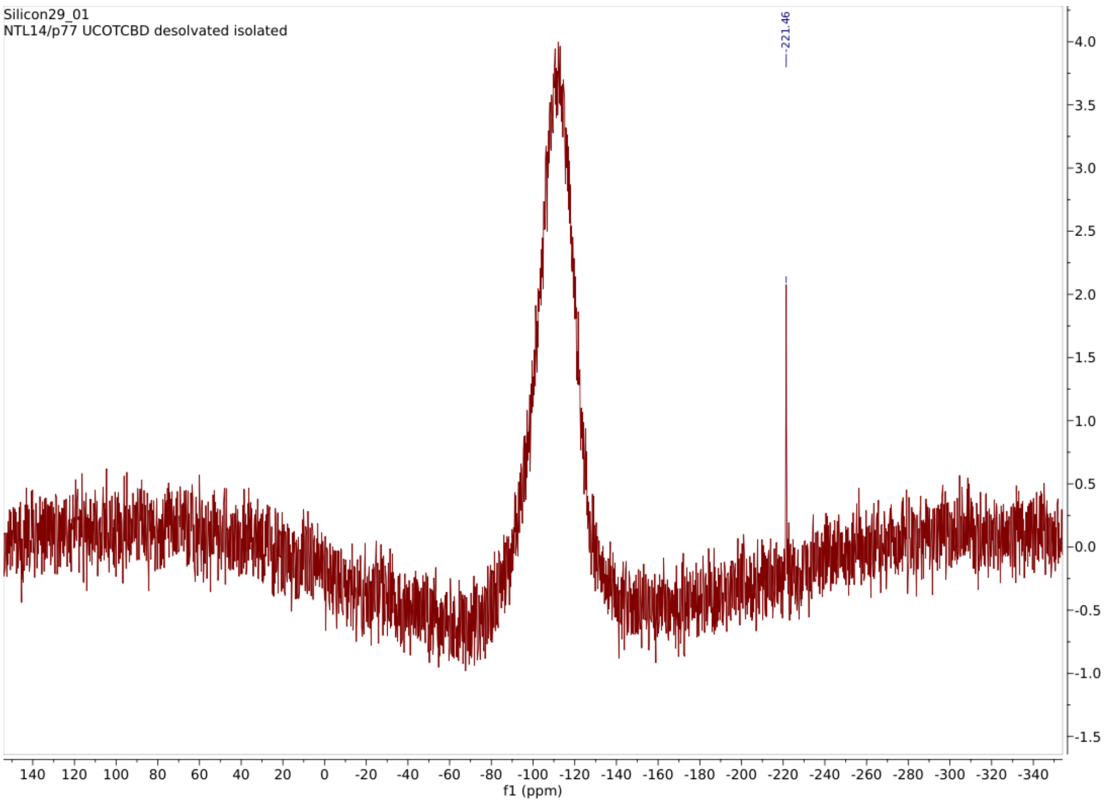

Figure S24. $^{29}Si\{^1H\}$ NMR spectrum of **3** in toluene-D_8 at 30°C.

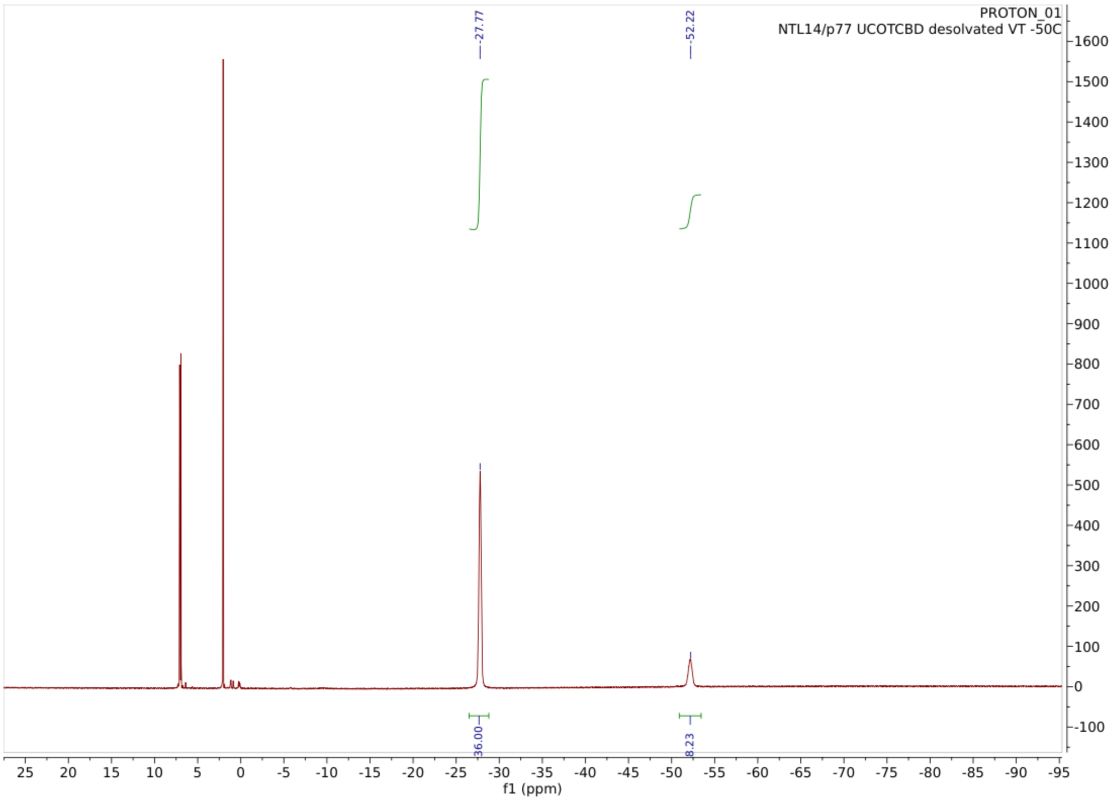

Figure S25. ^{1}H NMR spectrum of **3** in toluene-D_8 at –50°C.

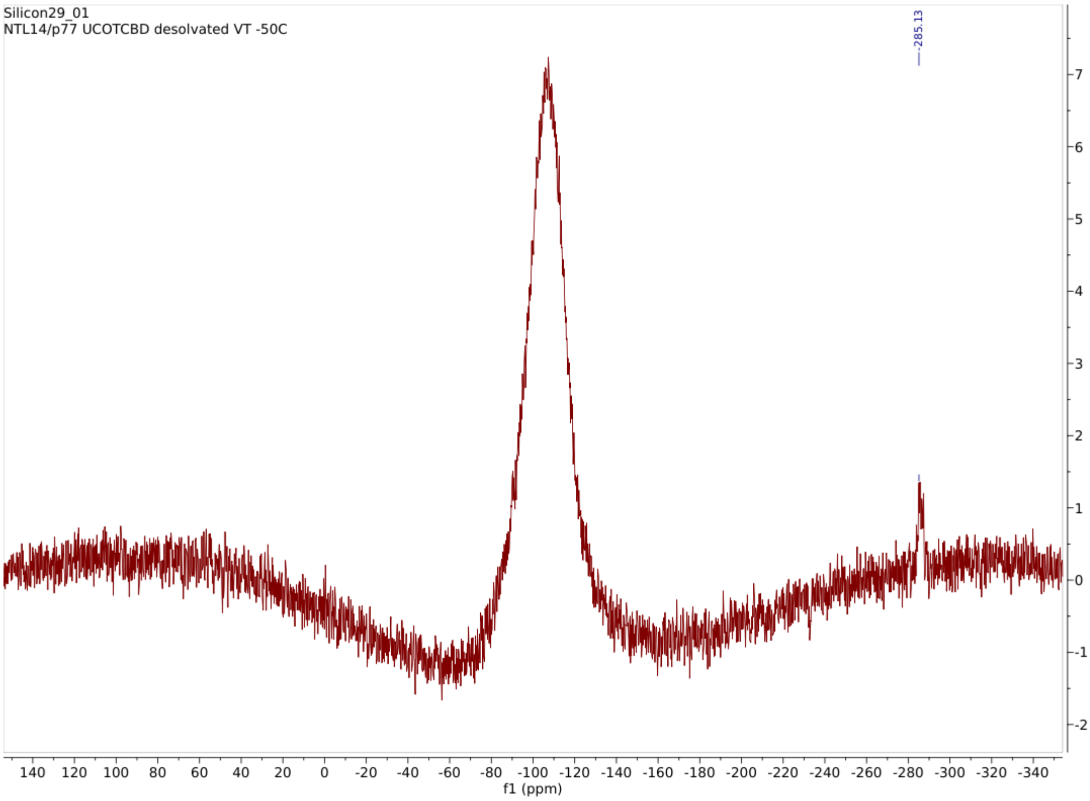

Figure S26. $^{29}Si\{^{1}H\}$ NMR spectrum of **3** in toluene-D_8 at –50 °C.

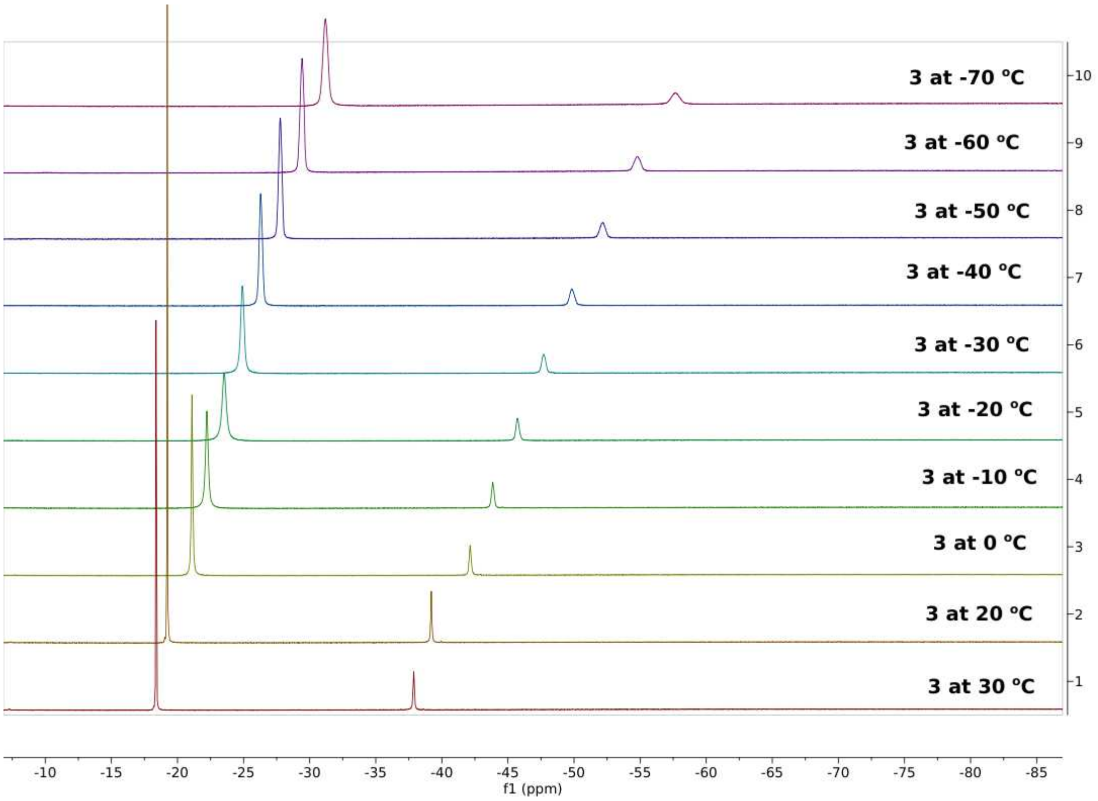

Figure S27. Variable-temperature ^{1}H NMR spectra of **3** in toluene-D_8.

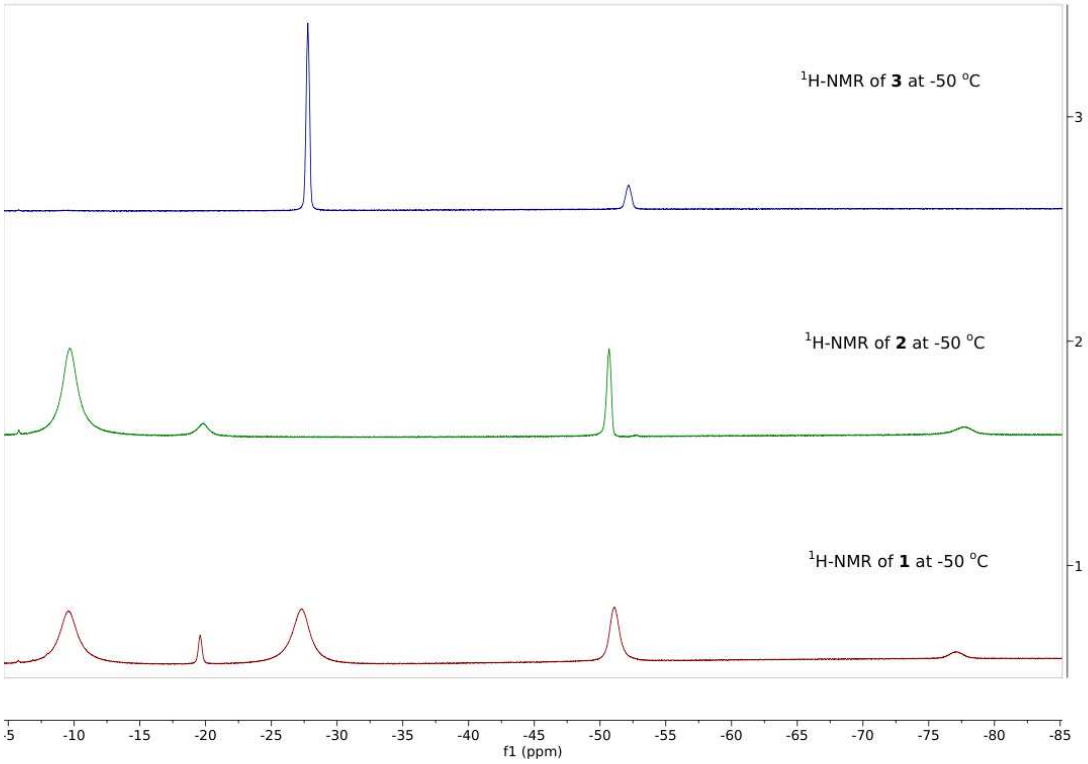

Figure S28. ^{1}H NMR spectra of [(Cb'''')(COT)U(COT)U(THF)(Cb'''')] **1** (bottom), [(Cb'''')U(THF)(COT)] **2** (middle), [(Cb'''')U(THF)(COT)] **3** (top) in toluene-D_8 at –50°C.

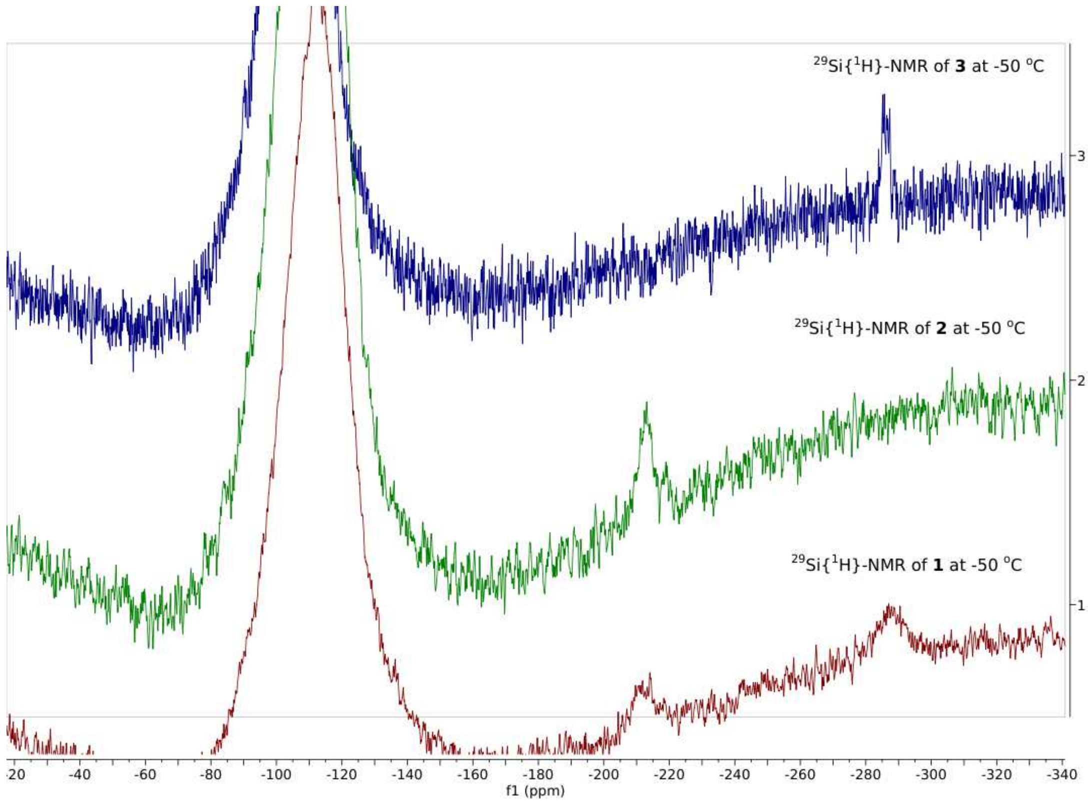

Figure S29. ^{29}Si{^{1}H} NMR spectra of [(Cb'''')(COT)U(COT)U(THF)(Cb'''')] **1** (bottom), [(Cb'''')U(THF)(COT)] **2** (middle), [(Cb''')U(THF)(COT)] **3** (top) in toluene-D$_8$ at –50°C.

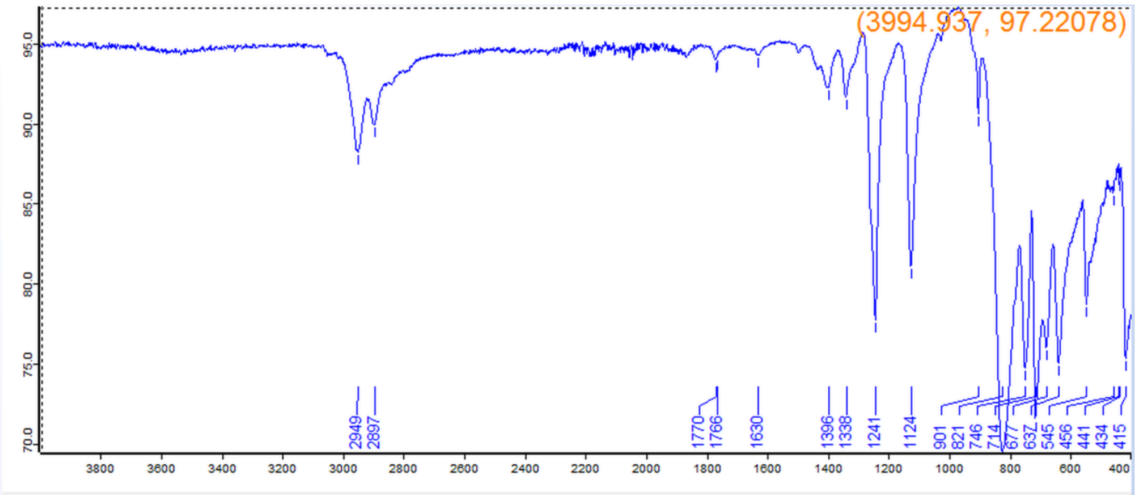

Figure S30. IR spectrum of **3**.

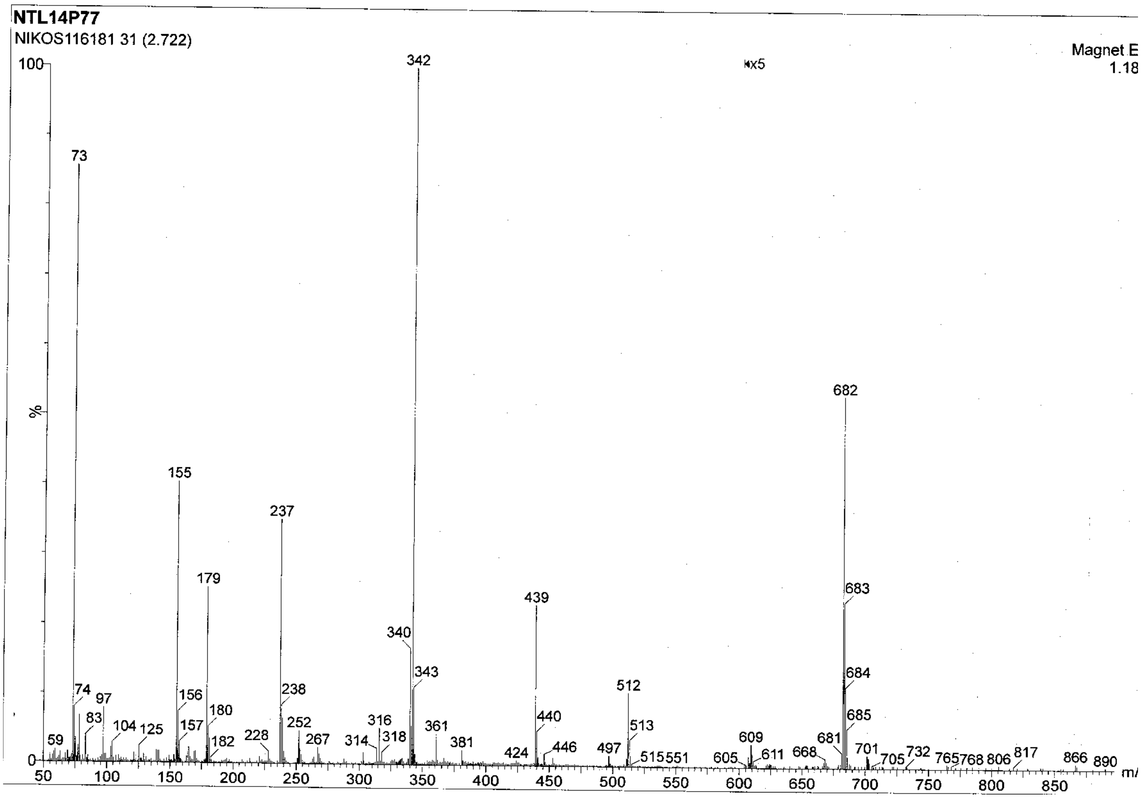

Figure S31. EI-MS spectrum of **3**.

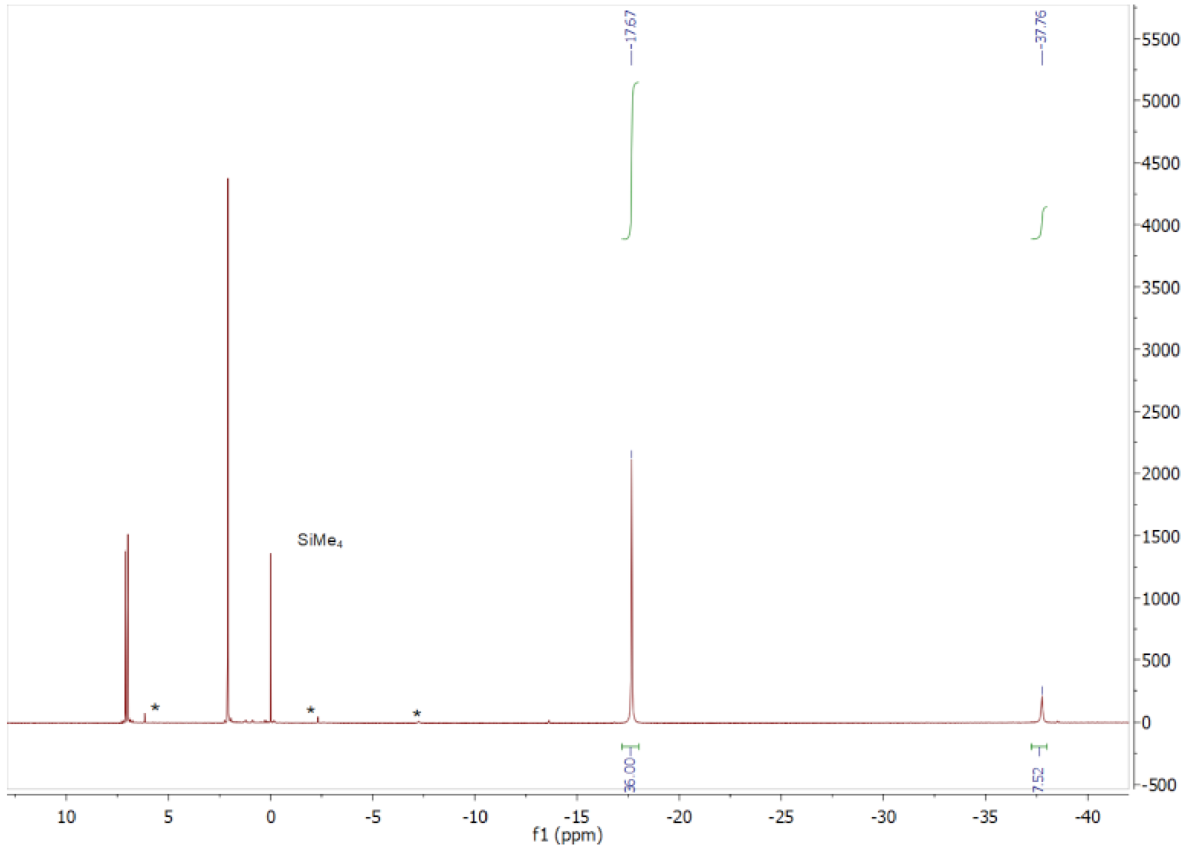

Figure S32. ^{1}H NMR spectrum of the co-crystal of **2/3** in toluene-D8. Peaks denoted with an asterisk correspond to a minor, unknown impurity, accounting for approximately 0.7% of the spectrum.

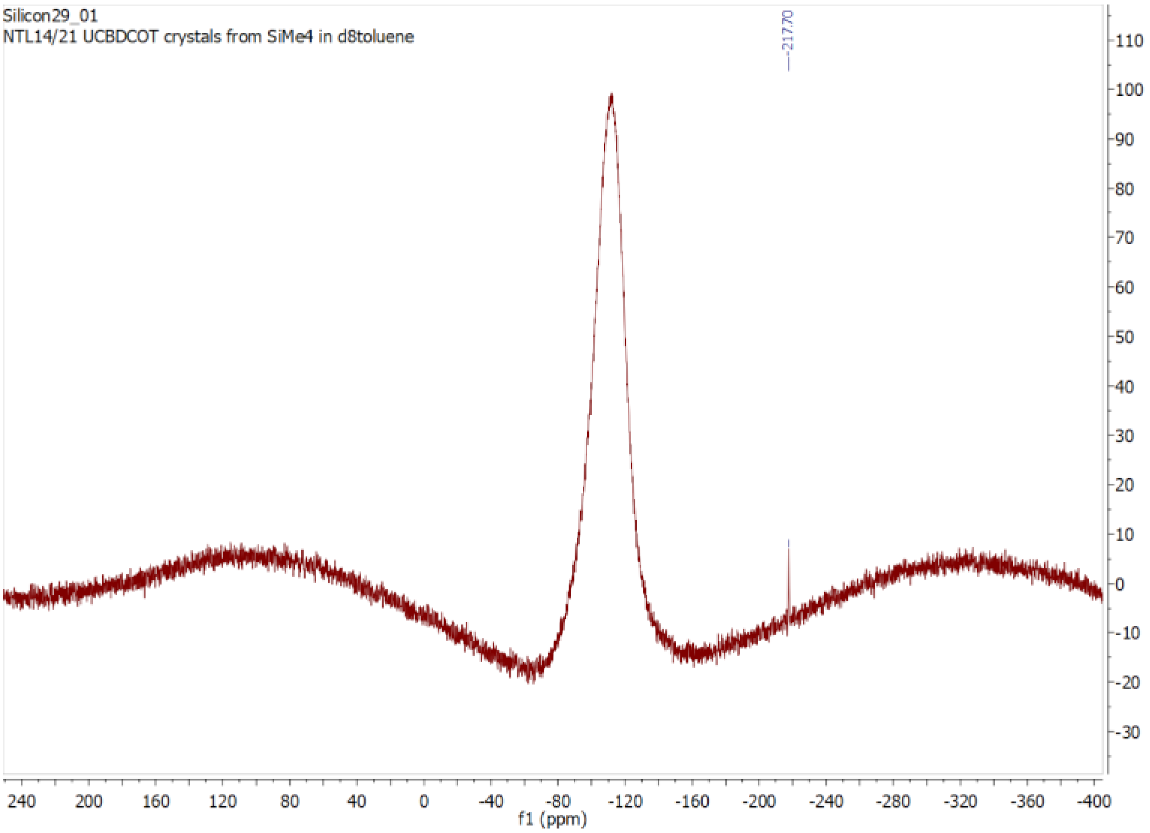

Figure S33. $^{29}Si\{^{1}H\}$ NMR spectrum in toluene-D_8 of the co-crystal of **2**/**3**.

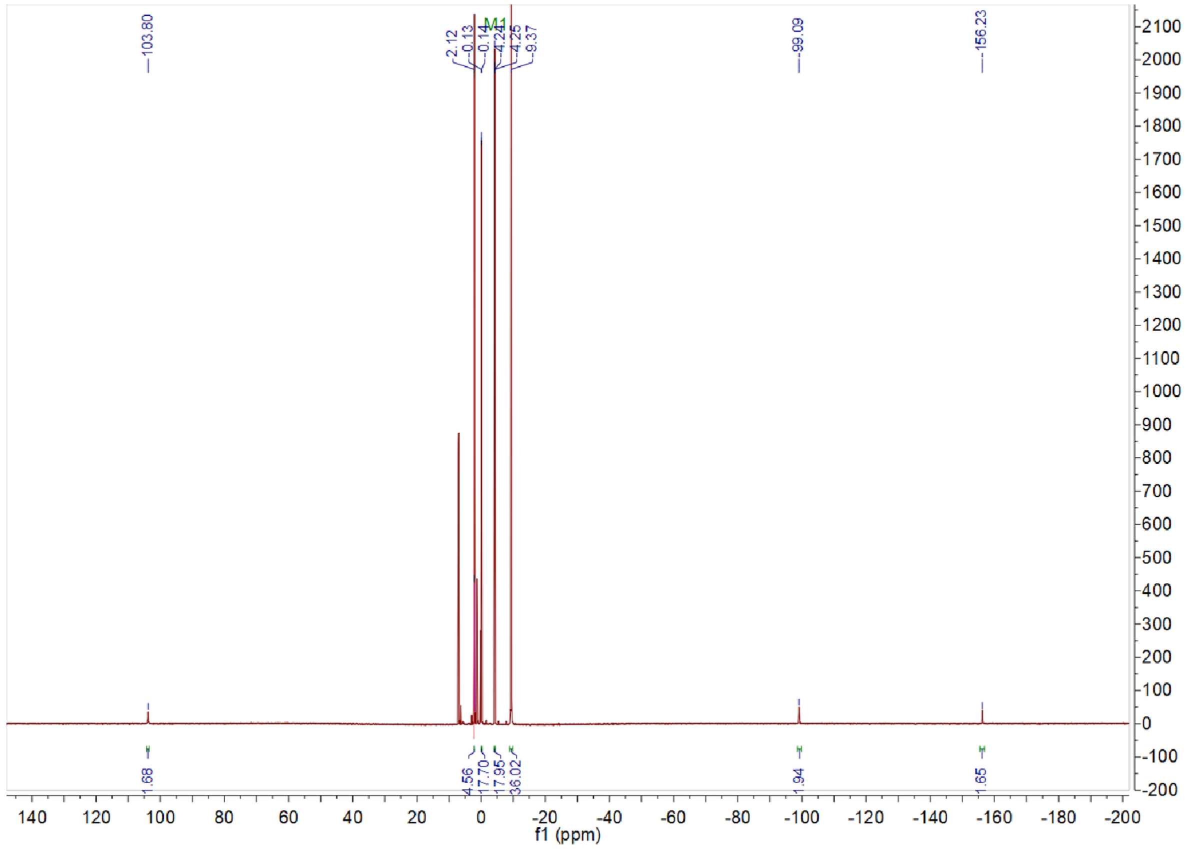

Figure S34. ^{1}H NMR spectrum of **4** in toluene-D_8.

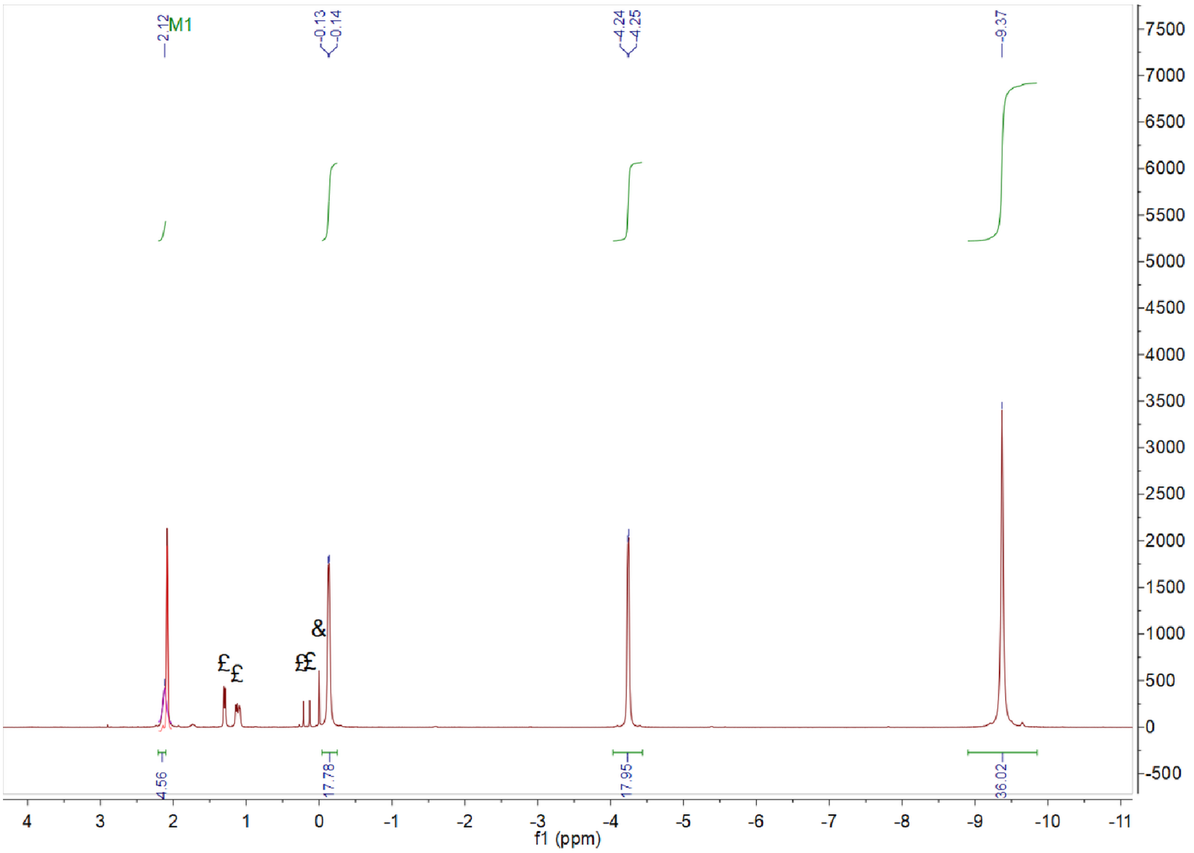

Figure S35. Expanded ^{1}H NMR spectrum of **4** in toluene-D$_8$ (& = SiMe$_4$, £ = unidentified minor impurities).

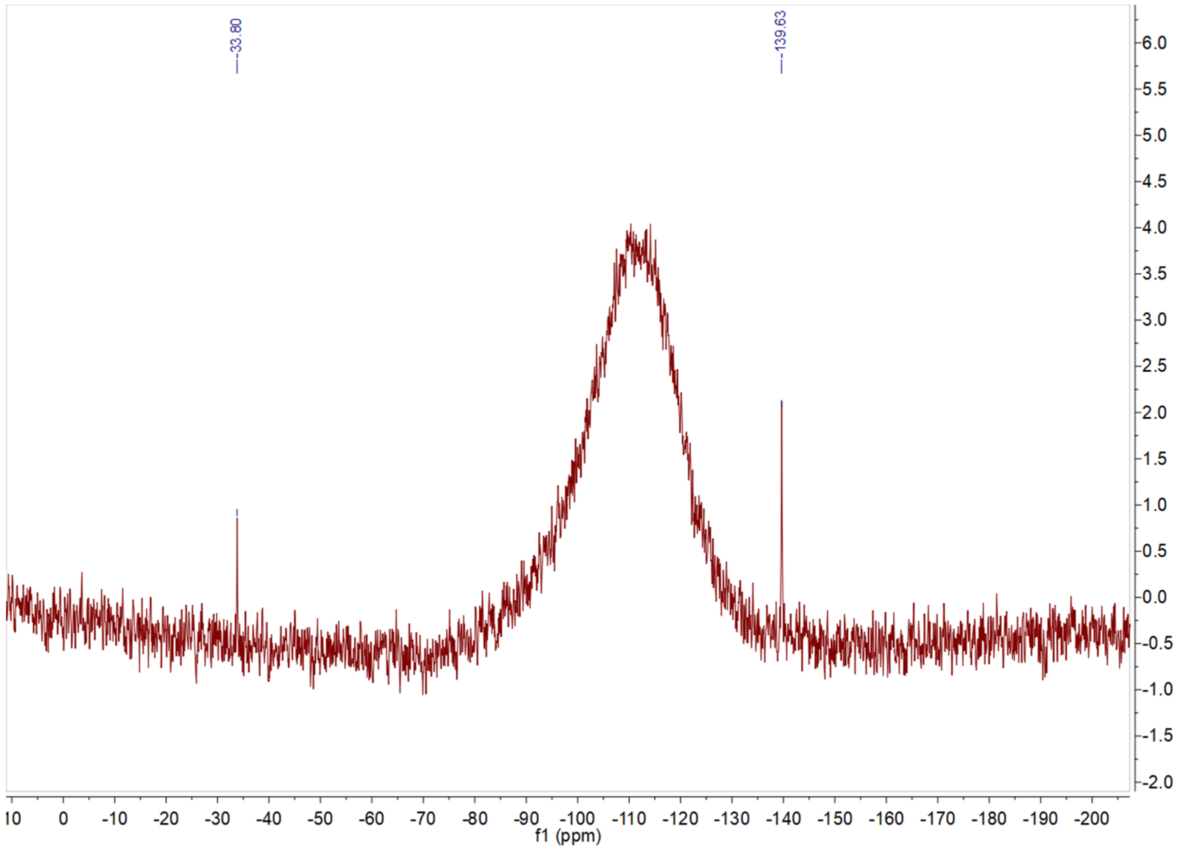

Figure S36. $^{29}Si\{^{1}H\}$ NMR spectrum of **4** in toluene-D_8.

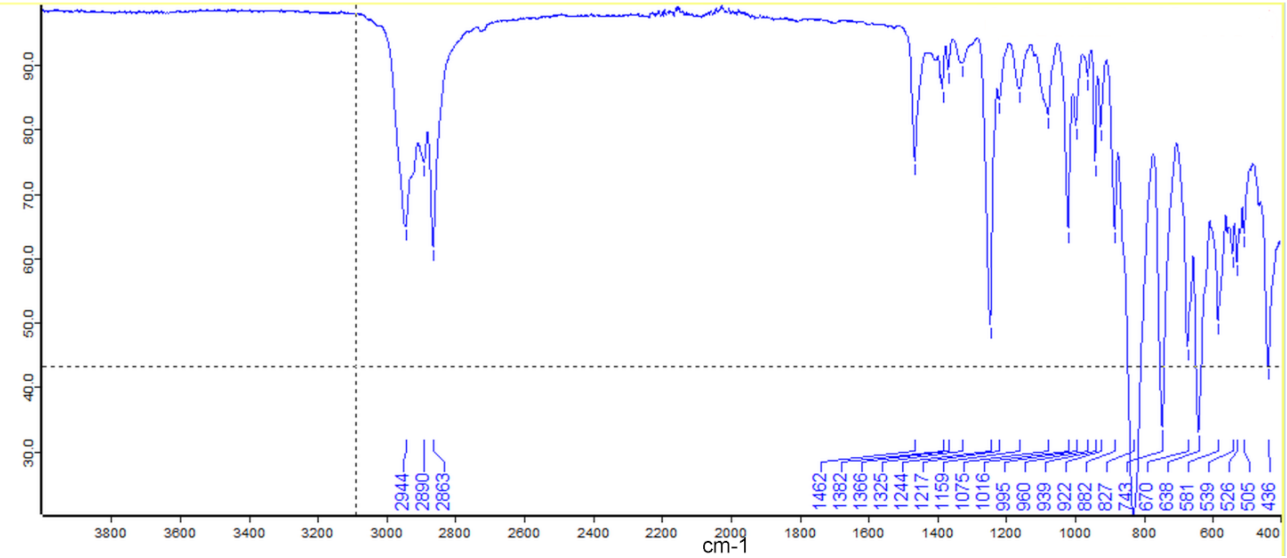

Figure S37. IR spectrum of **4**.

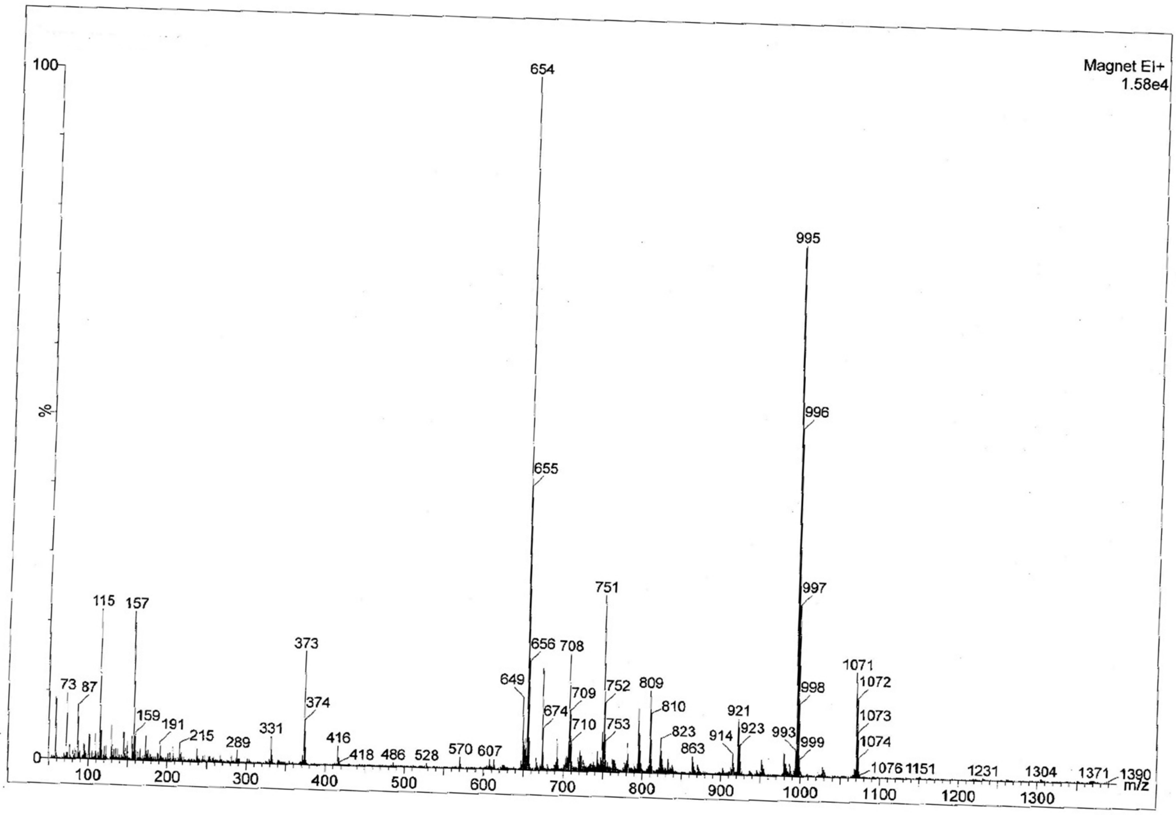

Figure S38. EI-MS spectrum of **4**.

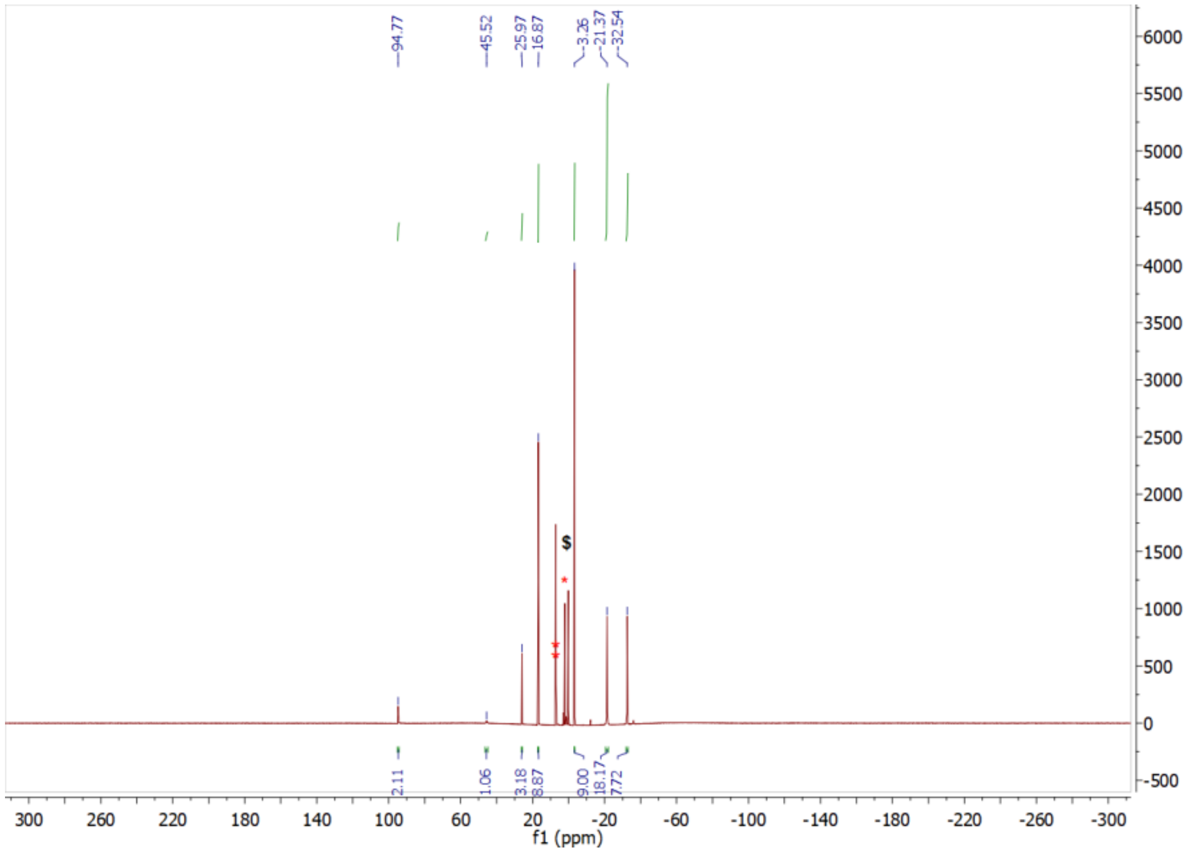

Figure S39. ^{1}H NMR spectrum benzene-D$_6$ after extraction of the reaction mixture of K_2COT and Na[U(η^4-Cb'''')(BH_4)$_3$] in Et_2O/toluene, standing overnight at room temperature and removal of volatiles (* = toluene, $ = unknown impurity).

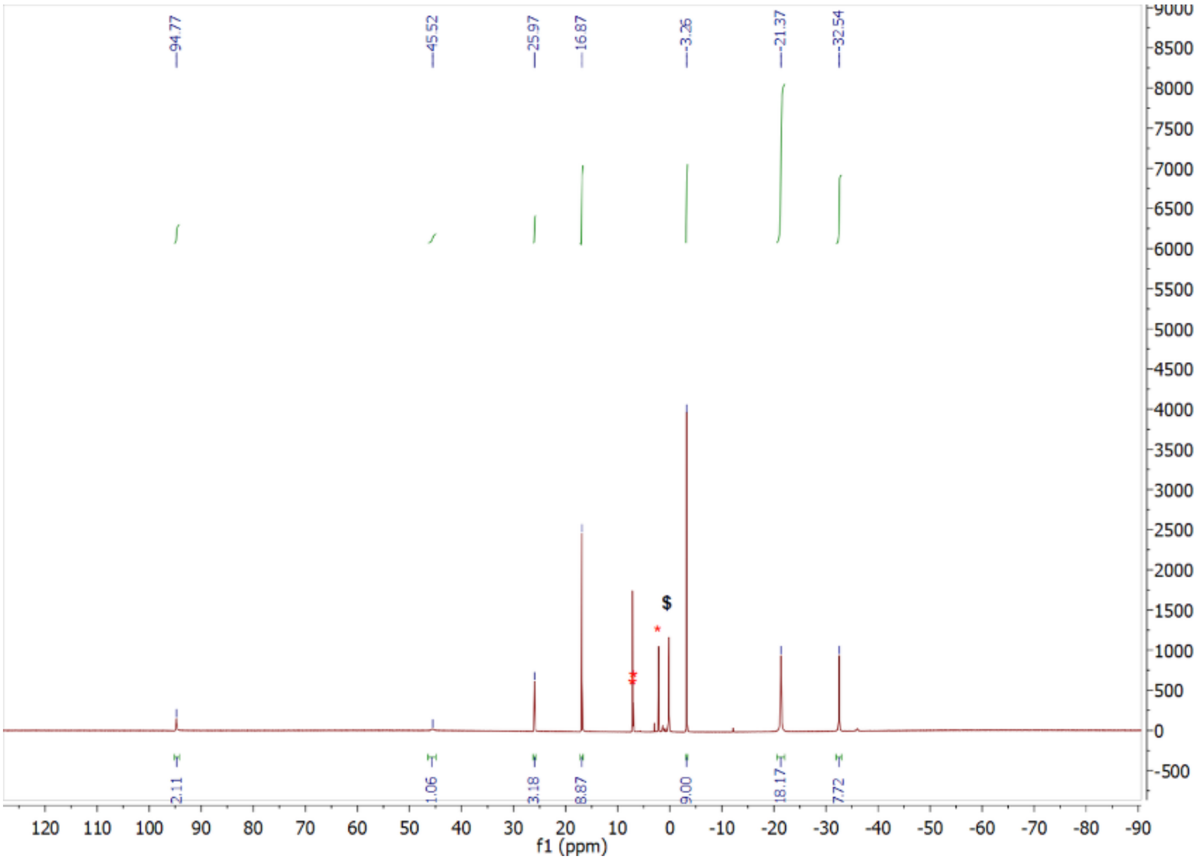

Figure S40. Expanded ^{1}H NMR spectrum in benzene-D$_6$ of the reaction mixture of K_2COT and Na[U(η^4-Cb'''')(BH_4)$_3$] after extraction of the reaction mixture of K_2COT and Na[U(η^4-Cb'''')(BH_4)$_3$] in Et_2O/toluene, standing overnight at room temperature and removal of volatiles (* = toluene, $ = unknown impurity).

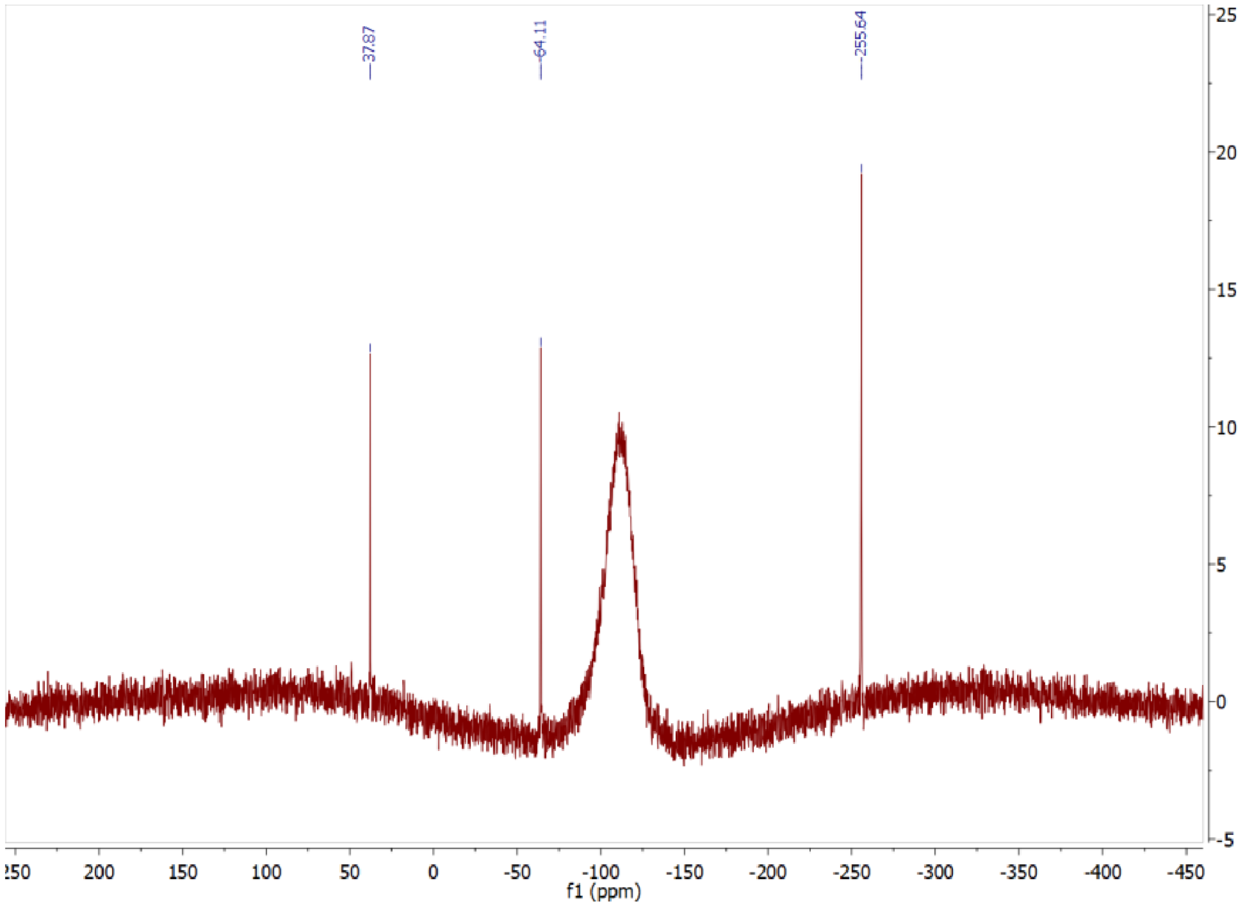

Figure S41. $^{29}Si\{^1H\}$ NMR spectrum in benzene-D_6 of the reaction mixture of K_2COT and Na[U(η^4-Cb'''')(BH_4)$_3$] after extraction of the reaction mixture of K_2COT and Na[U(η^4-Cb'''')(BH_4)$_3$] in Et_2O/toluene, standing overnight at room temperature and removal of volatiles.

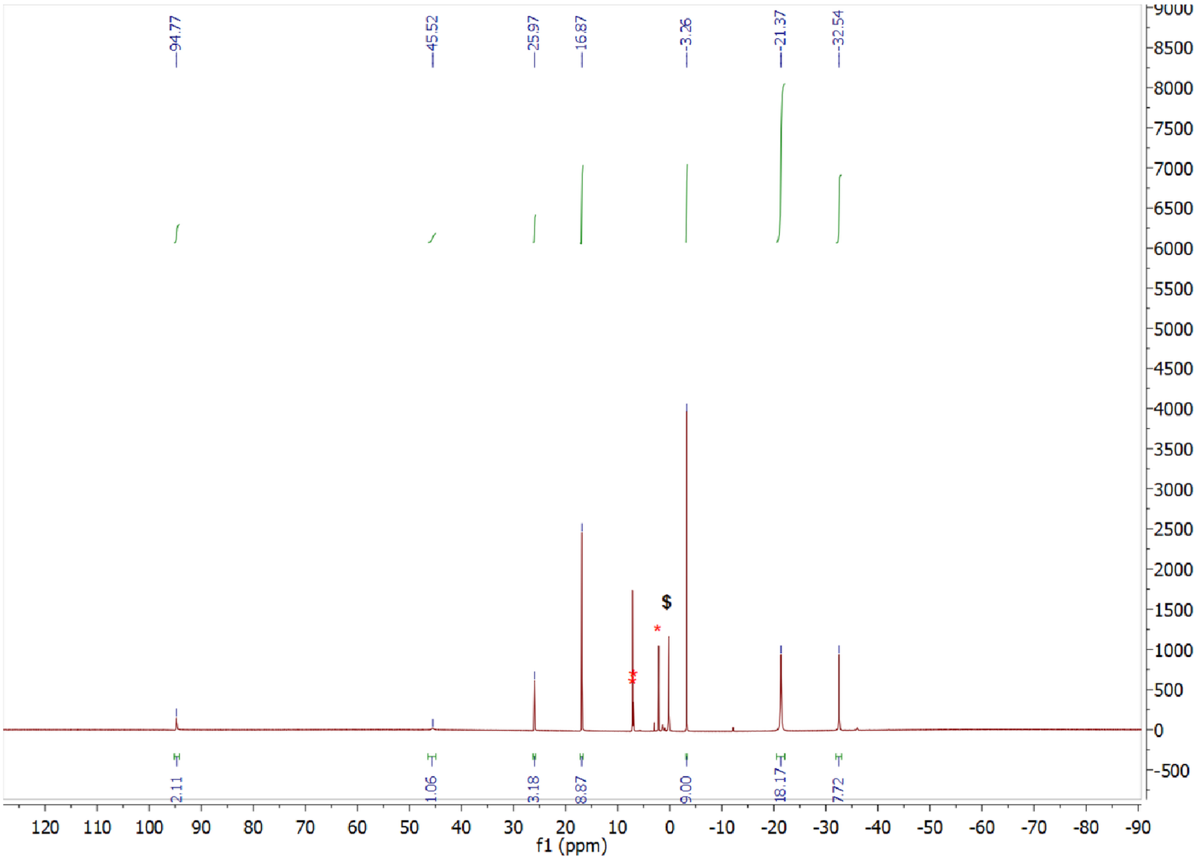

Figure S42. ^{1}H NMR spectrum in benzene-D_6 of isolated **5** (* = toluene, $ = unknown impurity).

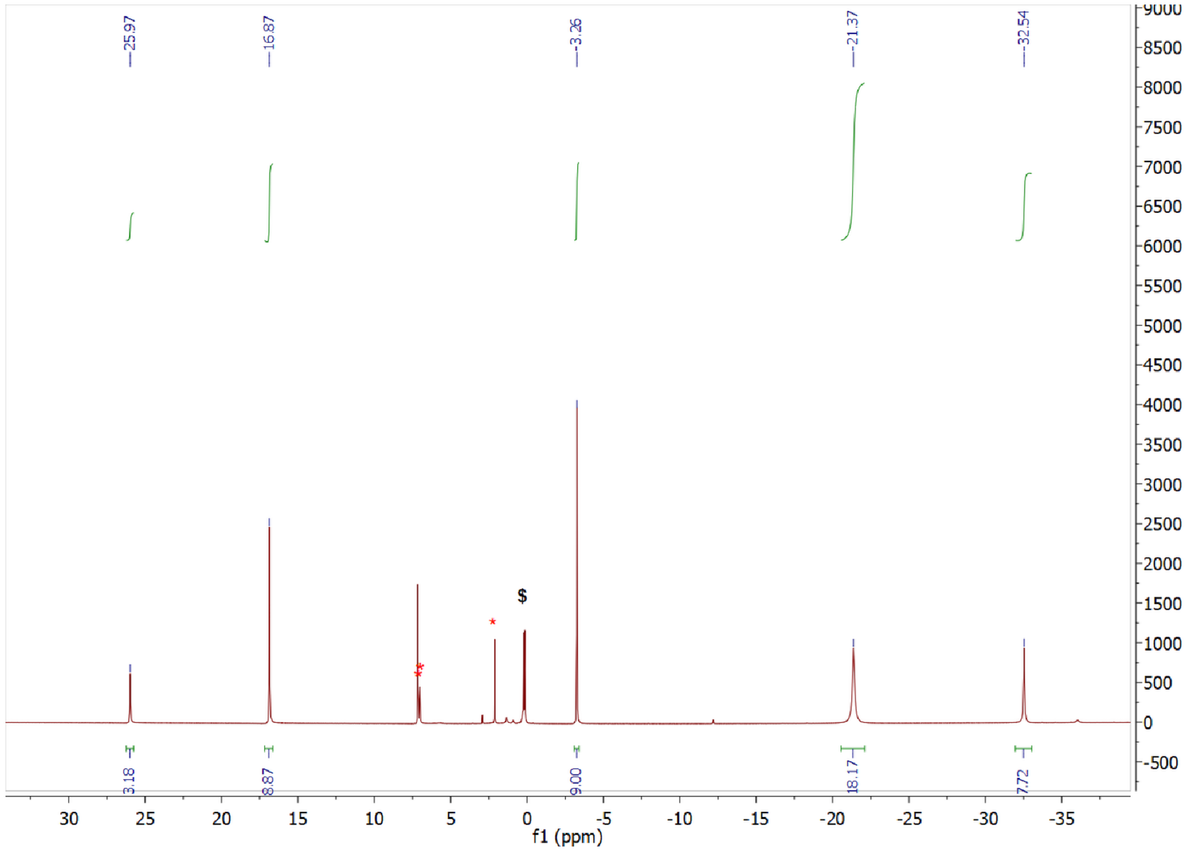

Figure S43. Expanded ^{1}H NMR spectrum of isolated **5** in benzene-D_6 (* = toluene, $ = unknown impurity).

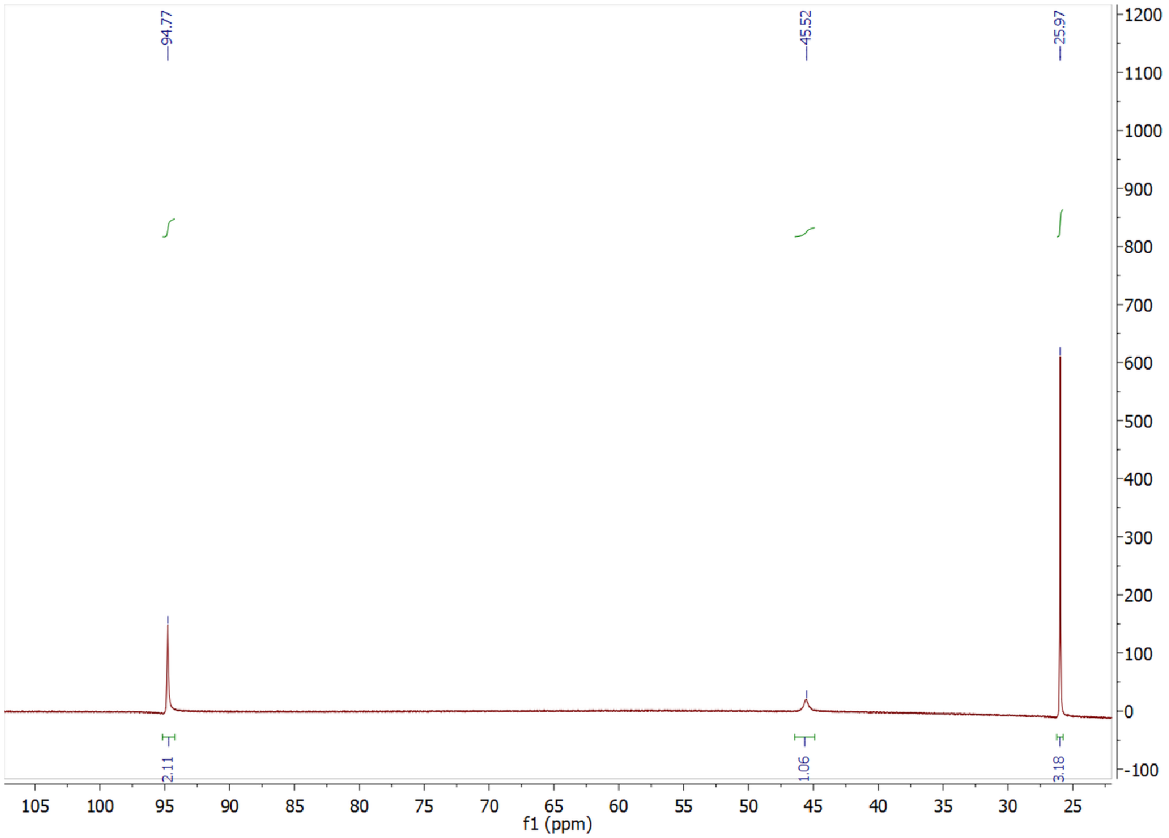

Figure S44. Expanded ^1H NMR spectrum of isolated **5** in benzene-D_6.

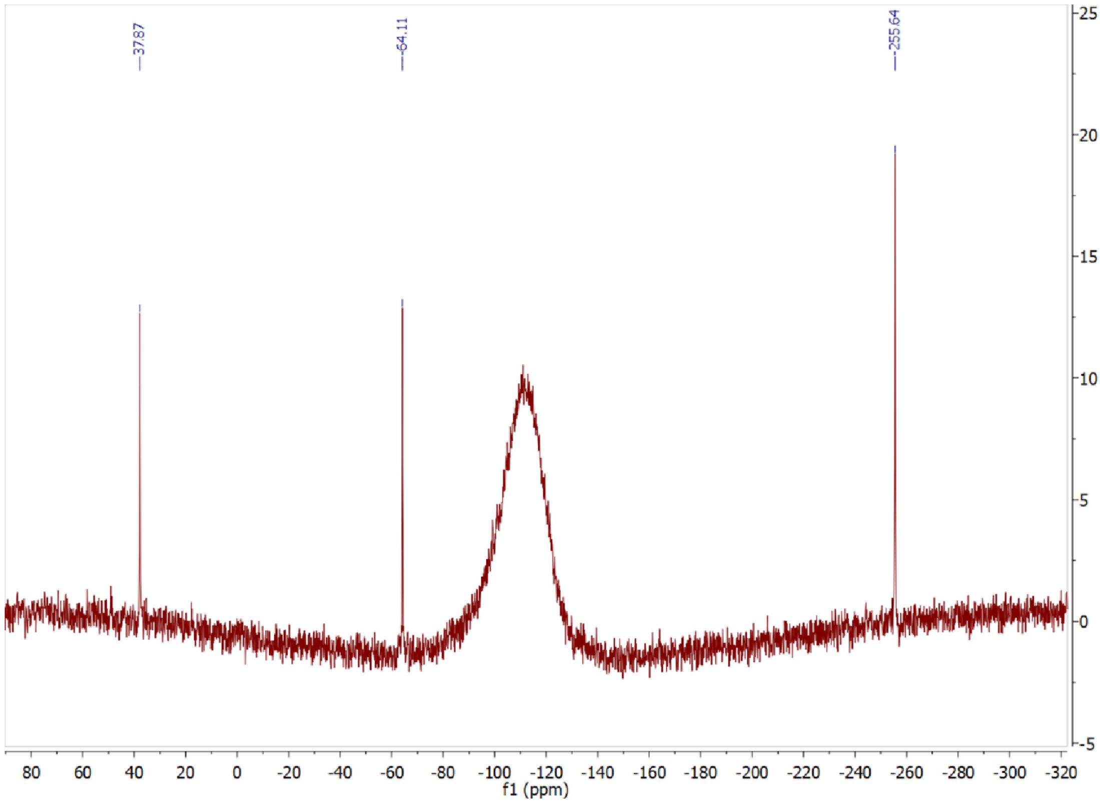

Figure S45. $^{29}Si\{^{1}H\}$ NMR spectrum of isolated **5** in benzene-D$_6$.

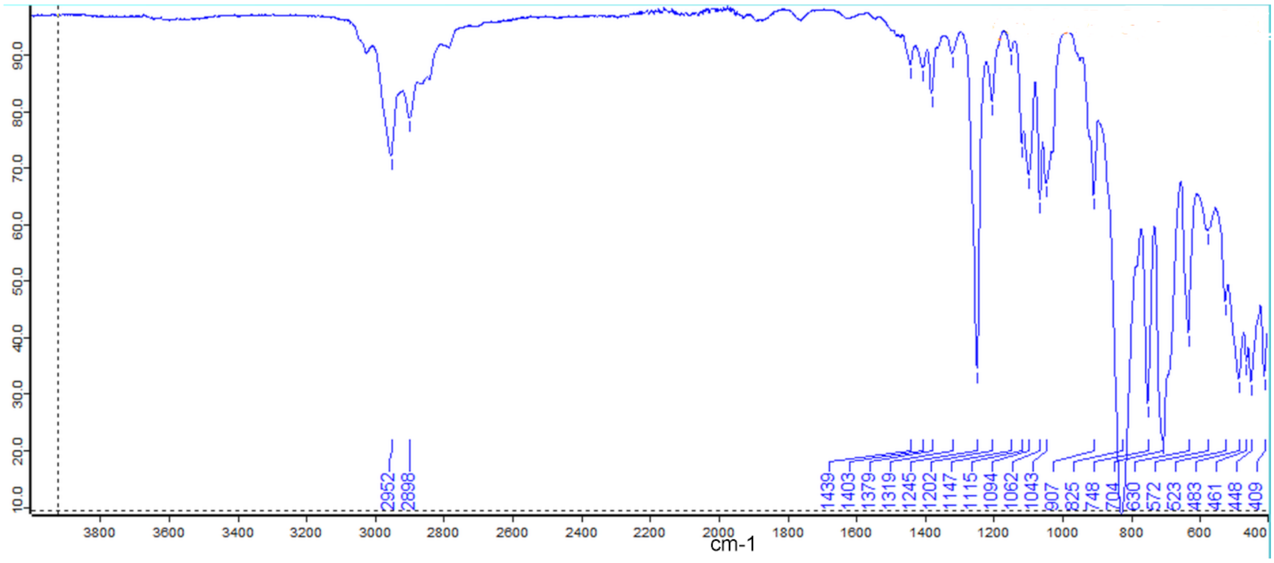

Figure S46. IR spectrum of **5**.

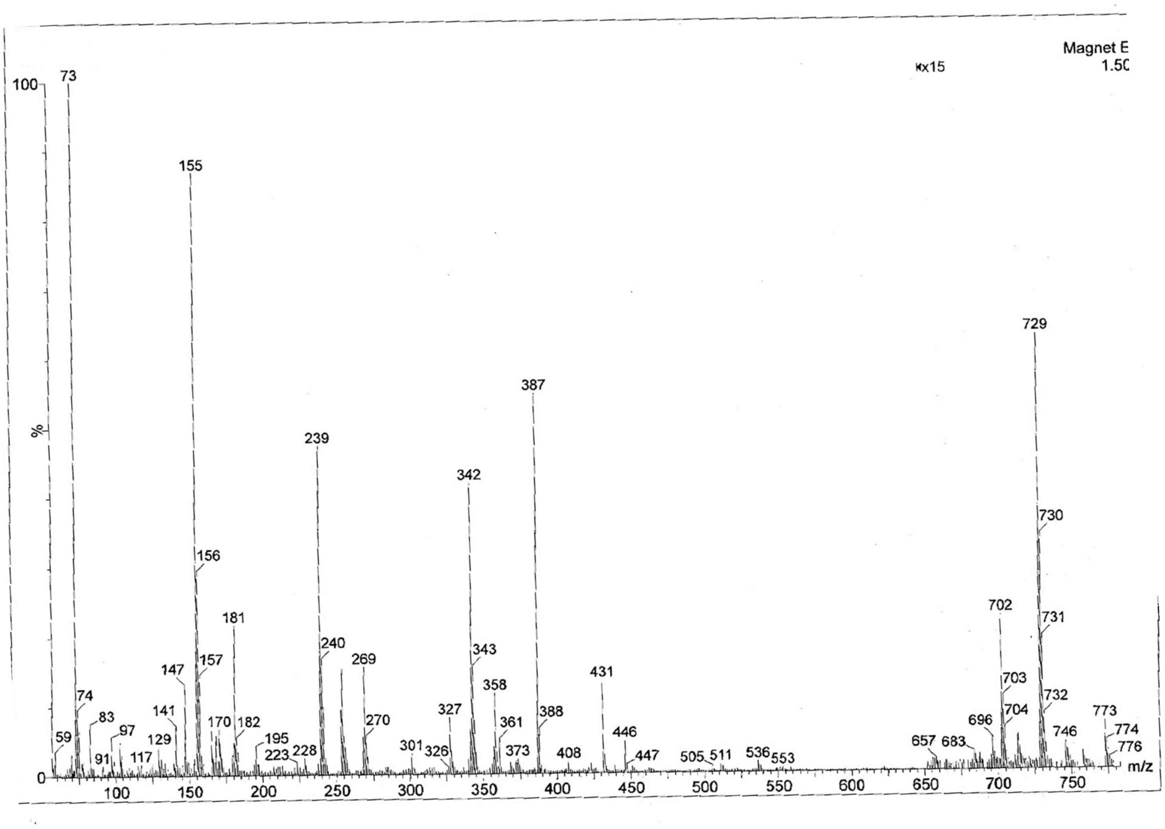

Figure S47. EI-MS spectrum of **5**.

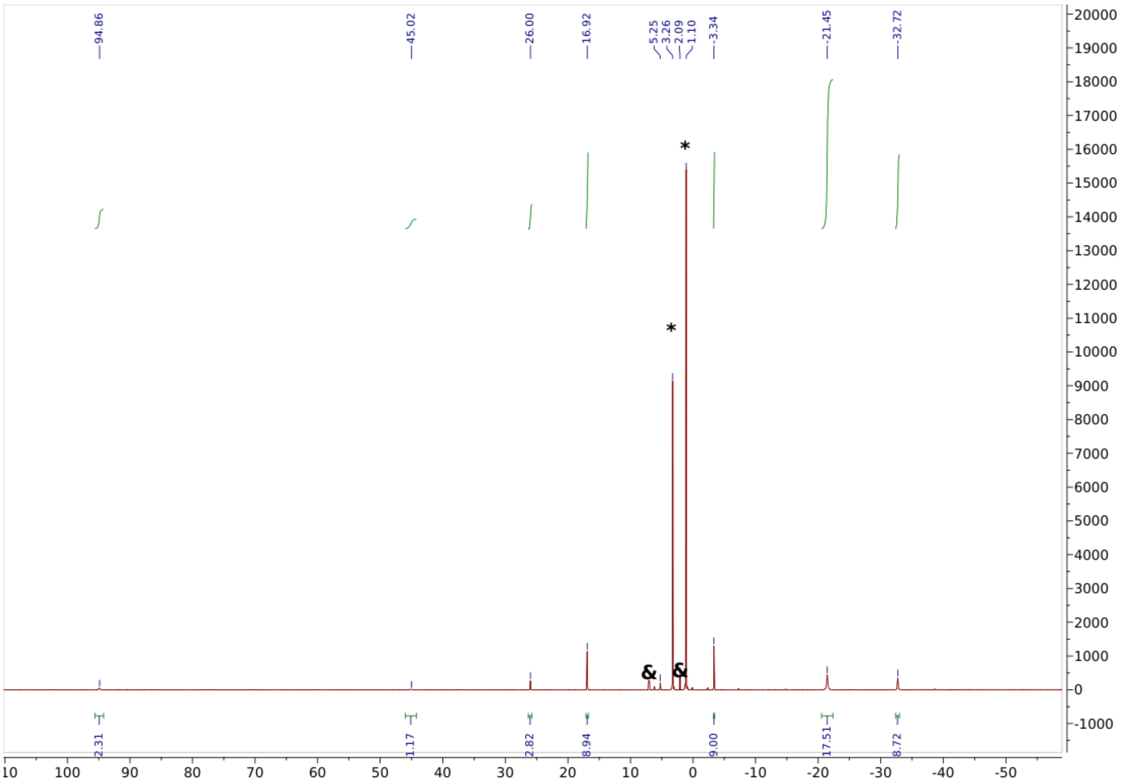

Figure S48. ^{1}H NMR spectrum of the reaction of **1** with Et_2O in toluene-D_8 (& = toluene protio signals, * = Et_2O).

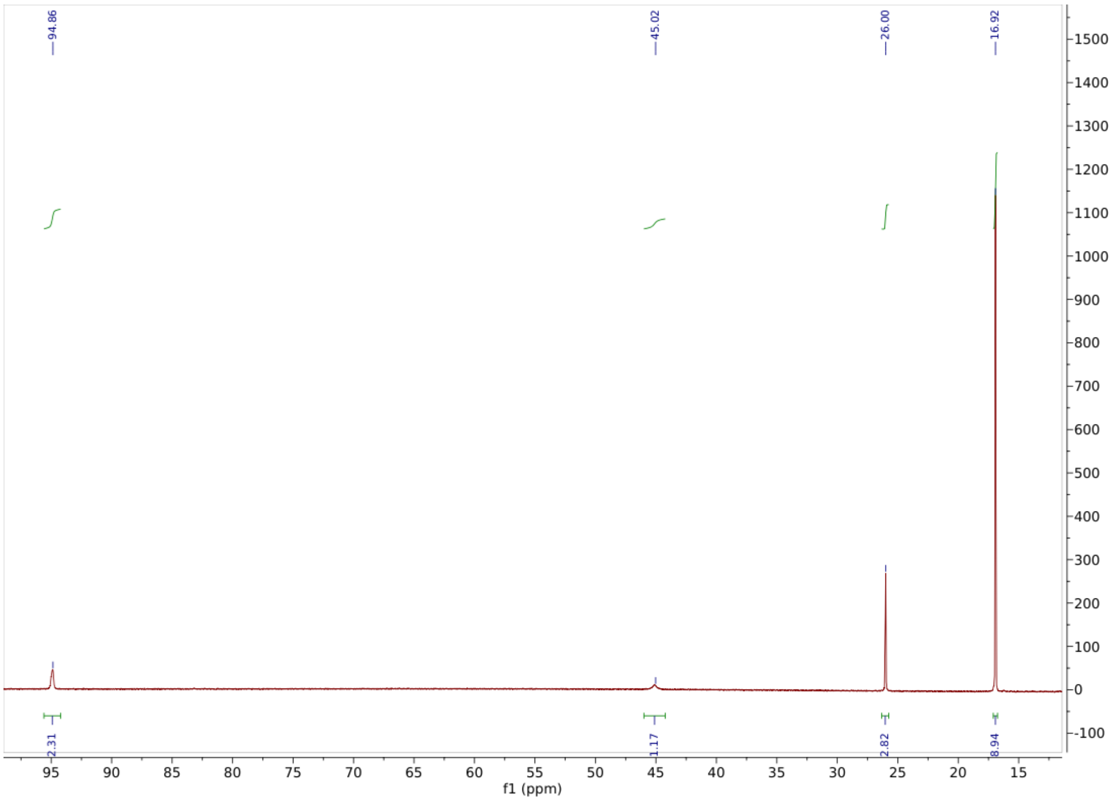

Figure S49. Expanded ^{1}H NMR spectrum of the reaction of **1** with Et_2O in toluene-D_8.

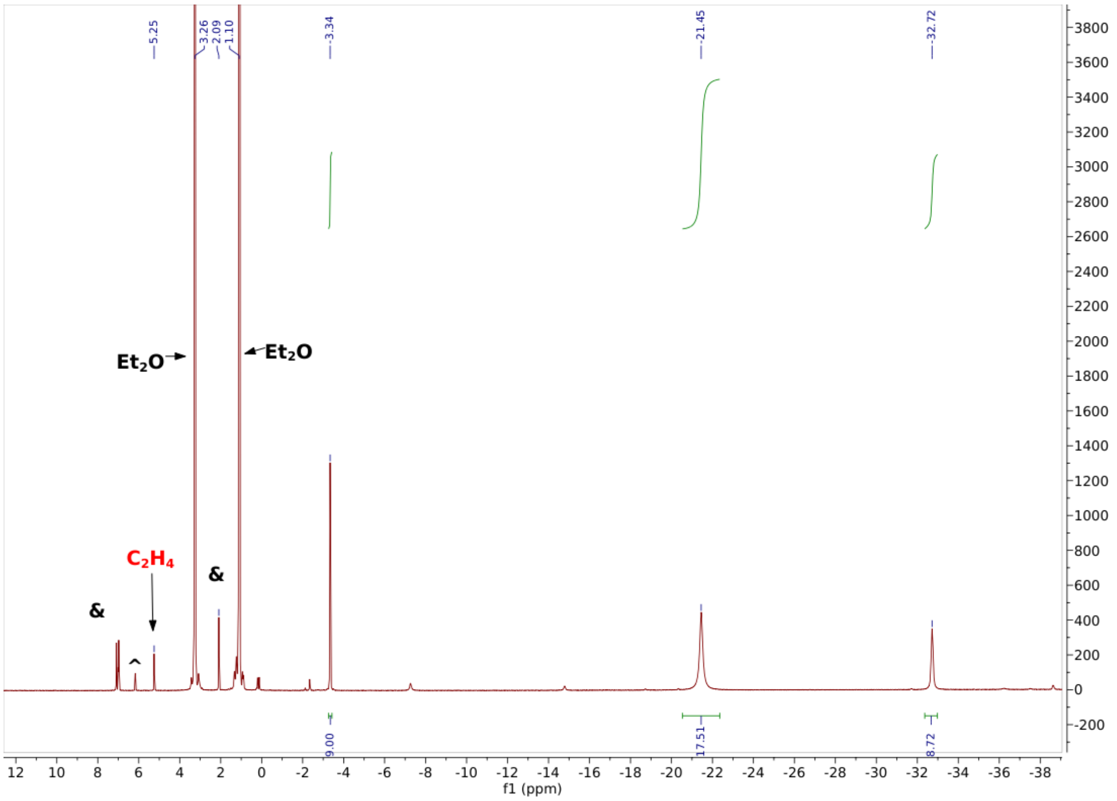

Figure S50. Expanded ^{1}H NMR spectrum of the reaction of **1** with Et_2O in toluene-D_8 (& = toluene protio signals, ^ = unknown impurity).

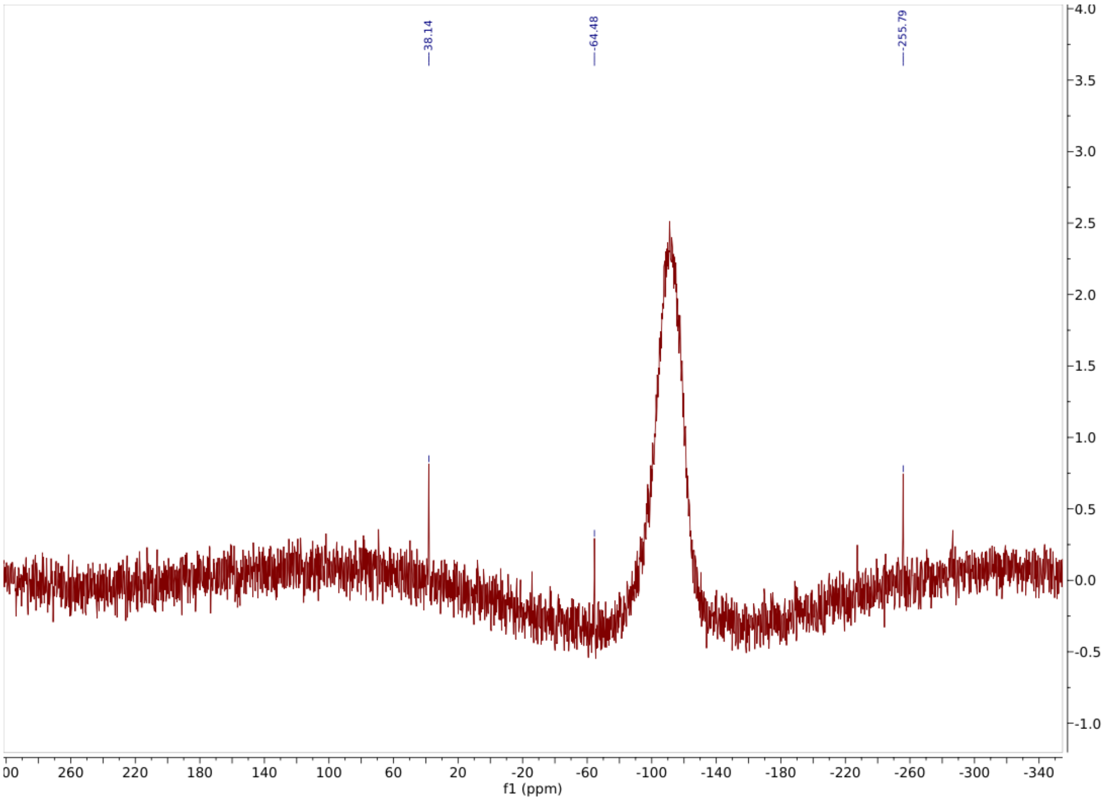

Figure S51. $^{29}Si\{^{1}H\}$ NMR spectrum of the reaction of **1** with Et_2O in toluene-D_8 showing **5** as the only detectable species.

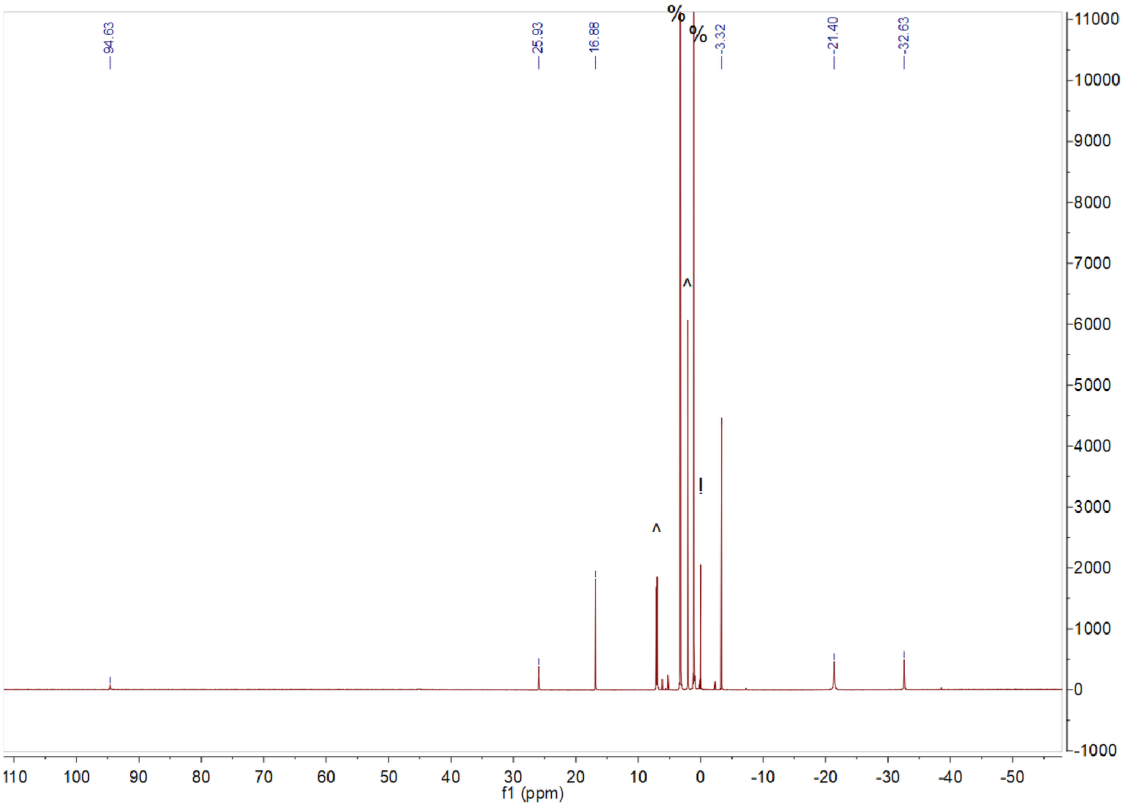

Figure S52. ^{1}H NMR spectrum of the reaction of **2/3** with Et_2O in toluene-D_8 (! = $SiMe_4$, ^ = toluene protio signals, % = Et_2O).

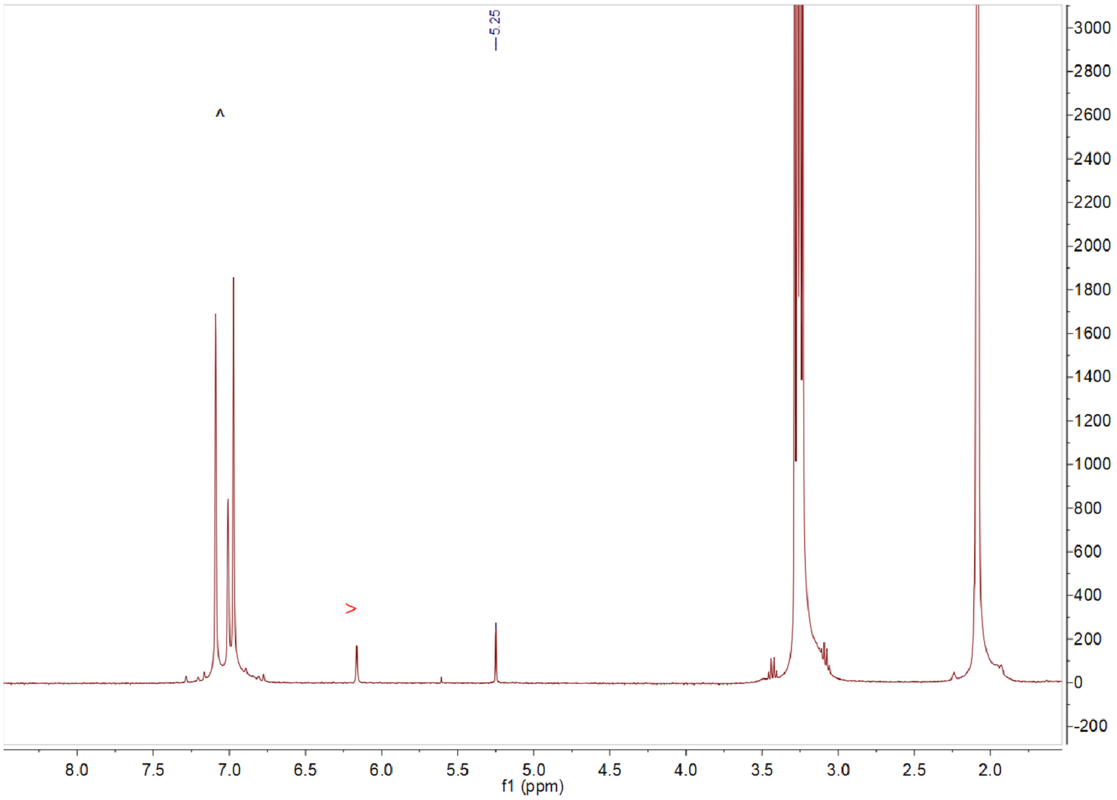

Figure S53. Expanded ^{1}H NMR spectrum of the reaction of **2/3** with Et_2O in toluene-D_8 showing the formation of C_2H_4 at 5.25 ppm (> = minor impurity).

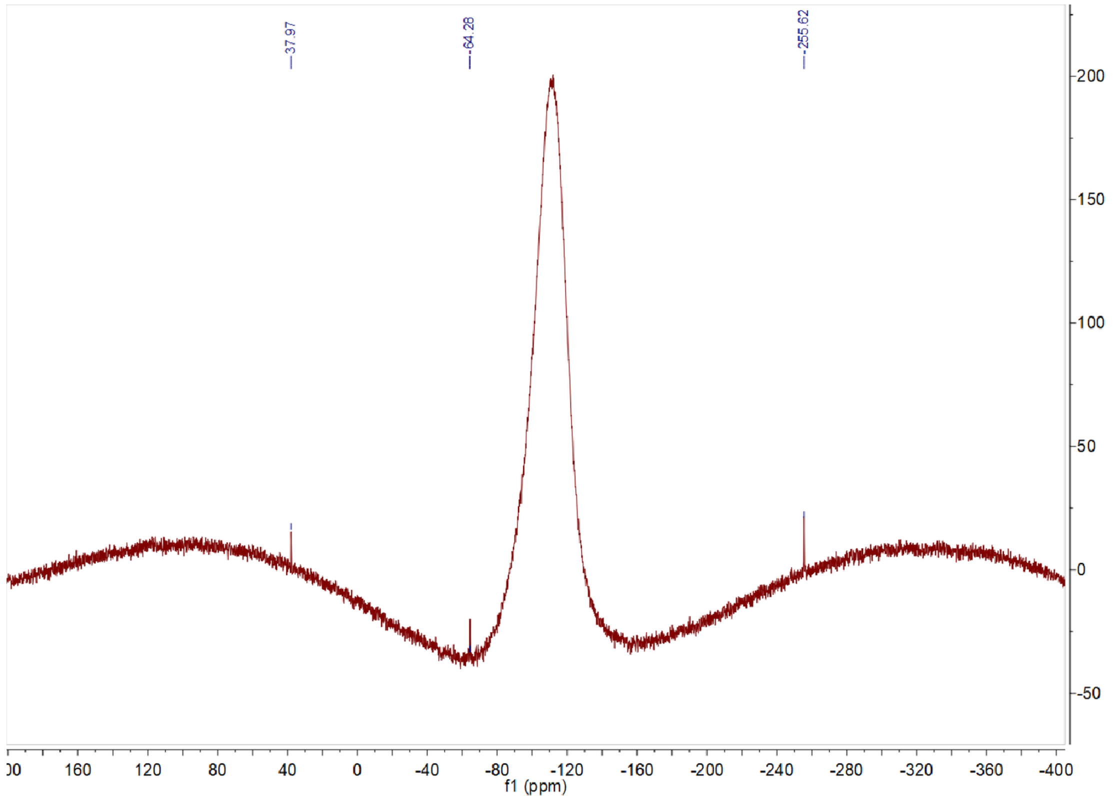

Figure S54. $^{29}Si\{^1H\}$ NMR spectrum of the reaction of **2/3** with Et_2O in toluene-D_8 showing **5** as the only detectable species.

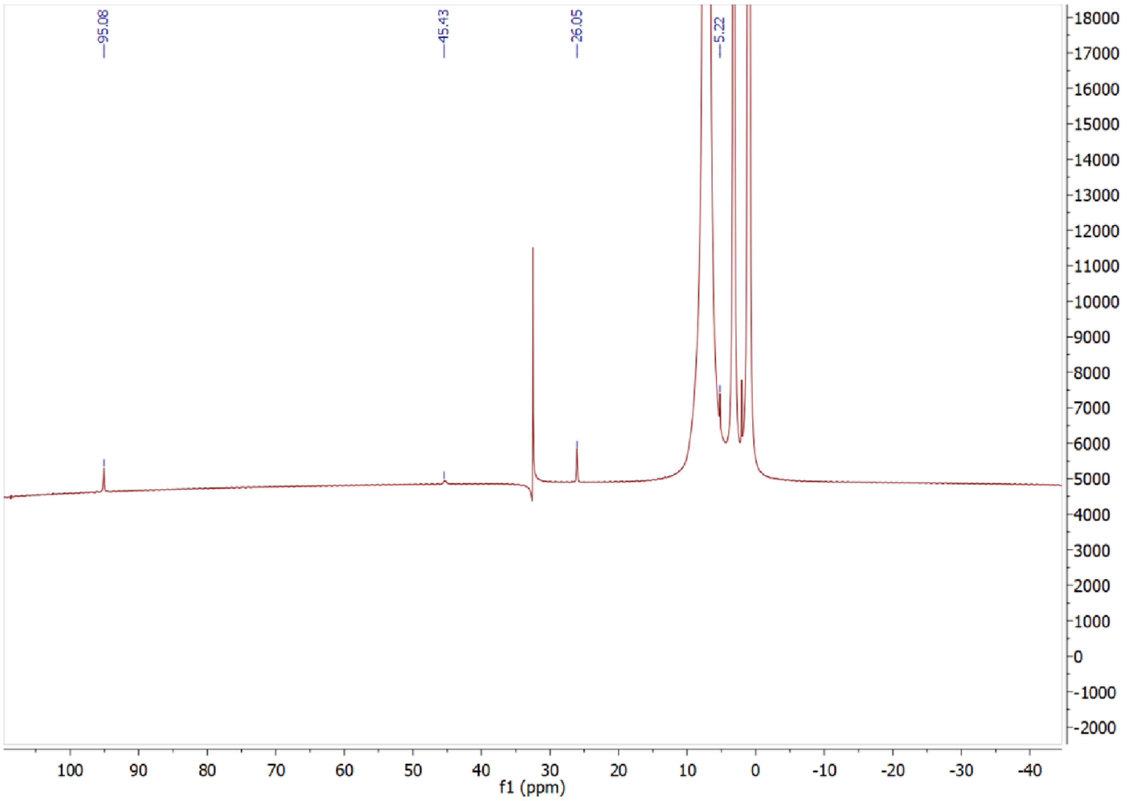

Figure S55. ^{2}H NMR spectrum in benzene-D_6 of the reaction of **2/3** with Et_2O-D_{10} after 10 hrs at room temperature. Signals are due to Cb''''D (45.43 ppm), OCD_2CD_3 (95.08 and 26.05 ppm, respectively) and C_2D_4 (5.22 ppm). The non-phasing peak at 32.5 ppm is an artefact in the middle of the spectrum width.

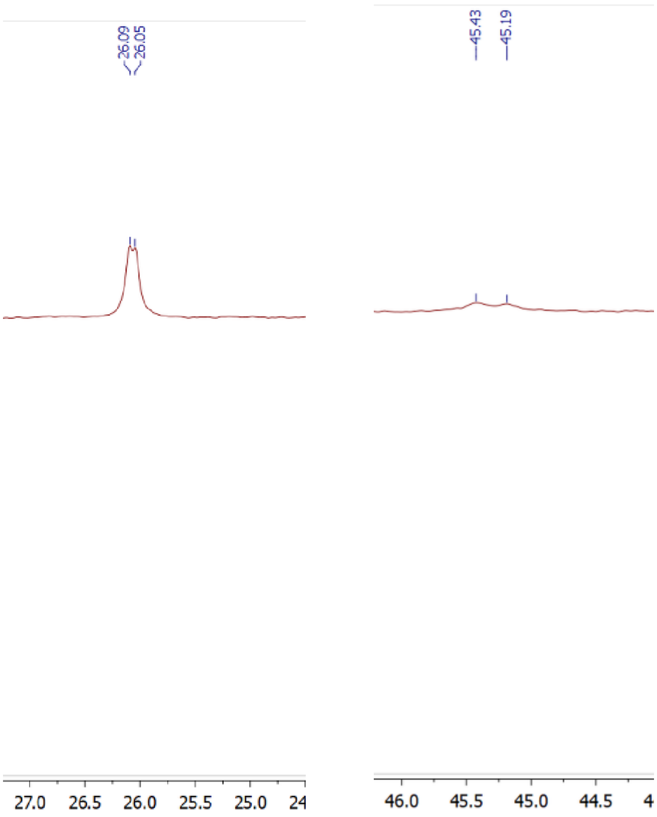

Figure S56. Expanded ^{2}H NMR spectra of **5** showing peaks corresponding to $OCD_2C\underline{D_3}$ (left) and Cb'''D (right) as virtual doublets.

Computational Details

All geometry optimizations were carried out using spin-unrestricted density functional theory as implemented in the Amsterdam Density Functional (ADF) 2019 software.[7,8] The pure generalized gradient approximation PBE[9,10] was used along with the empirical DFT-D3 dispersion correction[11] utilizing the Becke-Johnson damping function.[12] Scalar relativistic effects were introduced using the zeroth order regular approximation (ZORA).[13–15] All-electron Slater-type basis sets specifically designed for ZORA calculations were used in all geometry optimizations.[16] A valence triple-ζ quality basis with two sets of polarization functions (TZ2P) was used for the O, Si and U atoms and the C atoms of the COT and Cb rings; a valence triple-ζ quality basis with a single set of polarization functions (TZP) was used for the H atoms directly bonded to the COT and Cb rings; and polarized double-ζ quality basis sets (DZP) were used for all other atoms. The geometry convergence thresholds were increased to 10^{-4}, 10^{-4}, 10^{-3} and 10^{-1} atomic units for energy, energy gradient, bond length and bond angle, respectively. The "NumericalQuality" keyword in ADF was set to "Good" in all DFT calculations.

The orbital analysis was conducted using geometries extracted from the crystal structure with the positions of the hydrogen atoms optimized at the aforementioned level of theory. The orbitals were calculated as a single point energy evaluations using the PBE0 hybrid exchange-correlation functional.[9,10,17,18] The hybrid functional was chosen over the pure PBE functional to reduce excessive delocalization of the orbitals due to the delocalization error, and thus to reduce overestimation of covalency. Other computational details were kept the same as in the geometry optimizations. The reported orbital decomposition is based on non-orthogonal fragment orbitals associated with U, COT and Cb fragments used as a basis in the final calculation. The orbitals of the COT and Cb ligands were variationally optimized for the respective fragment. Due to limitations of the ADF code, the fragment orbitals of the U ion were calculated using restricted formalism and the occupations of the fragment orbitals were manually tweaked in the final calculation. This results into non-variational fragment orbitals. This level of theory is similar enough to our earlier work[3] to allow direct comparison of results.

The free energy for the coordination of the THF ligand was calculated using fully optimized geometries. Additional frequency calculations were carried out to ensure that the stationary geometries corresponded to true minima on the potential energy surface and to produce the vibrational spectra necessary for the evaluation of the free-energy correction to the internal energy. The geometry optimization was carried out at the aforementioned level and the free-energy correction was evaluated at the same level using a temperature of 298.15 K. The reported energies are Gibbs free energies calculated at the same level as the geometry optimization was performed. To ensure that the DFT results provide reasonably accurate energetics, the bonding energy of **2** was also calculated using the domain-localized pair-natural orbital coupled cluster singles and doubles theory with perturbative triples (DLPNO-CCSD(T))[19–23] using an unrestricted Hartree–Fock (UHF) reference in all calculations involved. The RI-JK approximation[24] was used in the UHF calculations. The DLPNO-CCSD(T) calculations were carried out using the *Orca* quantum chemistry software version 4.2.1.[25,26] Scalar relativistic effects were included using the standard second-order Douglas-Kroll-Heß (DKH) transformation.[27,28] The all-electron double-polarized valence triple-ζ basis SARC-DKH-TZVPP was used for the uranium ion.[29] Polarized valence triple-ζ quality DKH-def2-TZVP basis sets were used for O and Si atoms as well as the C atoms in the COT and Cb rings. Polarized valence double-ζ quality DKH-def2-SVP basis sets were used for other atoms. The DKH-def2-TZVP and DKH-def2-SVP use the same primitive functions as Ahlrichs' respective basis sets but have been re-contracted for DKH calculations.[30] The necessary auxiliary basis sets were generated using the "AutoAux" feature in *Orca*.[31] The calculated –69 kJ mol^{-1} and –82 kJ mol^{-1} at DFT and DLPNO-CCSD(T) levels, respectively, and are reasonably close. The distortion energies of the molecular fragments were calculated from single-point evaluations on the respective fragments at DFT level. The reported dispersion energy contributions to the free energies were calculated from the DFT-D3 corrections.

Table S2. Energies (E), enthalpies (H) and entropy corrections (TS) calculated for the various molecules and fragments at different levels of theory reported in Hartree atomic units

Geometry		E(PBE) [a]	H [a]	$-TS$ [a]	E(DFT-D3) [a]
**3**	Relaxed	−14.95060296	−14.43868688	−0.11919781	−0.12146949
**2** (**3**·THF)	Relaxed	−17.60052393	−16.99120154	−0.13366916	−0.14491330
**3**	Distorted	−14.94220932			−0.11989195
THF(**3**) [b]	Distorted	−2.63693423			−0.00927703
**4**	Relaxed	−26.69511539	−25.72744434	−0.18265383	−0.22706063
**4**·THF	Relaxed	−29.32795912	−28.26518649	−0.19587035	−0.25419660
**4**	Distorted	−26.66685643			−0.22419785
THF(**4**) [b]	Distorted	−2.63671182			−0.00925352
THF	Relaxed	−2.63785467	−2.52796004	−0.03437770	−0.00924519

[a] These quantities calculated by ADF correspond to the bonding energy between atomic fragments and not to the total energy of the system, which is not available in a standard ADF calculation.

[b] The compound number in the parenthesis refers to the adduct geometry the distorted geometry has been extracted from.

Table S3. Energies used in the DLPNO-CCSD(T) calculation of the energy of formation of **2** (**3**·THF) reported in Hartree atomic units

Geometry	*E*(UHF)	*E*(DLPNO-CCSD(T))
**3**	–30041.76411984	–5.29529939
**2** (**3**·THF)	–30272.94389761	–6.28925932
THF	–231.10688727	–1.03564625

Table S4. Percentage contribution from different fragment orbitals to the valence molecular orbitals of **3**.

Fragment orbitals		Molecular orbitals									
		α 164	α 165	α 166	α 167	α 168	α 169	β 164	β 165	β 166	β 167
U	$6d_{z2}$	3.5	1.5	0.0	0.1	2.9	3.8	4.0	0.6	1.1	4.4
U	$6d_{x2-y2}$	0.3	1.8	0.1	4.0	1.9	0.8	0.7	2.6	3.7	0.9
U	$6d_{xy}$	2.8	0.6	0.1	0.1	0.2	4.2	3.0	0.0	0.2	2.9
U	$6d_{xz}$	5.4	0.9	0.4	1.2	1.8	0.0	4.0	3.1	0.6	0.3
U	$6d_{yz}$	0.0	3.3	0.1	1.5	1.2	8.5	0.0	5.7	1.1	2.9
U	$5f_{z3}$	2.9	0.3	7.9	11.3	12.0	0.5	1.0	0.4	1.8	3.6
U	$5f_{z(x2-y2)}$	0.2	2.7	0.5	15.6	0.1	0.1	0.0	0.1	2.6	0.7
U	$5f_{xyz}$	3.5	7.6	54.5	1.7	6.3	4.4	0.0	0.1	0.1	1.1
U	$5f_{xz2}$	1.4	5.5	12.8	2.3	1.5	6.6	0.2	1.2	0.6	1.2
U	$5f_{yz2}$	0.0	0.0	1.4	1.9	0.9	0.1	0.1	0.3	1.7	0.0
U	$5f_{x(x2-3y2)}$	0.3	10.9	7.8	13.2	0.0	2.3	1.2	0.6	1.0	0.0
U	$5f_{y(3x2-y2)}$	2.6	5.5	4.9	0.5	0.0	4.1	0.3	0.9	0.2	0.3
COT	HOMO	59.4	10.4	0.7	0.5	2.0	0.0	71.9	1.0	5.2	0.2
COT	HOMO	3.1	33.8	2.7	30.3	0.0	0.1	0.4	71.2	2.3	2.1
Cb	HOMO	5.2	2.0	0.6	4.5	47.4	5.4	4.8	2.1	63.3	0.6
Cb	HOMO	0.0	2.9	0.5	0.6	8.3	46.7	0.3	1.9	0.9	62.9

Table S5. Percentage contribution from different fragment orbitals to the valence molecular orbitals of **4**.

**Fragment orbitals**		**Molecular orbitals**									
		α 252	α 253	α 254	α 255	α 256	α 257	β 252	β 253	β 254	β 255
**U**	$6d_{z2}$	1.1	0.0	1.4	0.4	0.2	0.1	0.9	0.0	1.6	0.3
**U**	$6d_{x2-y2}$	0.2	5.5	1.3	0.5	0.2	0.2	0.7	4.8	1.5	0.1
**U**	$6d_{xy}$	6.2	0.2	0.2	1.4	1.8	0.0	5.0	0.6	0.1	1.5
**U**	$6d_{xz}$	1.9	2.6	2.0	0.9	0.1	0.9	0.7	2.7	2.6	1.1
**U**	$6d_{yz}$	0.6	2.3	2.8	6.6	0.1	0.6	1.3	1.9	2.2	6.2
**U**	$5f_{z3}$	1.6	0.7	3.0	3.0	5.9	11.8	1.3	0.1	2.0	1.9
**U**	$5f_{z(x2-y2)}$	0.0	0.9	4.5	0.6	9.4	4.3	0.1	0.4	1.7	0.2
**U**	$5f_{xyz}$	1.2	0.0	0.2	3.3	15.0	26.2	0.5	0.0	0.0	1.4
**U**	$5f_{xz2}$	0.8	2.1	3.4	0.1	25.7	0.8	0.3	1.6	2.5	0.1
**U**	$5f_{yz2}$	1.3	1.1	7.6	3.6	12.2	4.9	0.6	0.5	3.0	3.1
**U**	$5f_{x(x2-3y2)}$	2.0	0.6	0.1	1.5	7.4	21.1	1.5	0.1	0.0	0.2
**U**	$5f_{y(3x2-y2)}$	0.5	2.6	0.2	4.4	11.8	14.8	0.2	1.5	0.1	1.4
**COT**	HOMO	66.0	1.0	1.2	0.0	0.4	0.1	72.8	0.3	0.4	0.1
**COT**	HOMO	0.8	64.5	1.9	0.1	0.1	0.2	0.3	69.9	1.6	1.3
**Cb**	HOMO	1.4	1.3	53.2	0.9	1.9	2.5	0.6	1.5	64.6	0.6
**Cb**	HOMO	0.0	1.0	1.2	54.6	0.0	1.8	0.2	1.5	0.5	63.5

References

1 A. Haaland, D. J. Shorokhov, A. V Tutukin, H. V. Volden, O. Swang, G. S. McGrady, N. Kaltsoyannis, A. J. Downs, C. Y. Tang and J. F. C. Turner, *Inorg. Chem.*, 2002, **41**, 6646–6655.

2 A. Chakraborty, B. M. Day, J. P. Durrant, M. He, J. Tang and R. A. Layfield, *Organometallics*, 2020, **39**, 8–12.

3 N. Tsoureas, A. Mansikkamäki and R. A. Layfield, *Chem. Commun.*, 2020, **56**, 944–947.

4 C. D. Stevenson and R. M. Fico, *J. Org. Chem.*, 1995, **60**, 5452–5455.

5 N. C. Burton, F. G. N. Cloke, S. C. P. Joseph, H. Karamallakis and A. A. Sameh, *J. Organomet. Chem.*, 1993, **462**, 39–43.

6 R. C. Clark and J. S. Reid, *Acta Crystallogr. Sect. A*, 1995, **51**, 887–897.

7 G. te Velde, F. M. Bickelhaupt, E. J. Baerends, C. Fonseca Guerra, S. J. A. van Gisbergen, J. G. Snijders and T. Ziegler, *J. Comput. Chem.*, 2001, **22**, 931–967.

8 C. Fonseca Guerra, J. G. Snijders, G. te Velde and E. J. Baerends, *Theor. Chem. Acc.*, 1998, **99**, 391–403.

9 J. P. Perdew, K. Burke and M. Ernzerhof, *Phys. Rev. Lett.*, 1996, **77**, 3865–3868.

10 J. P. Perdew, K. Burke and M. Ernzerhof, *Phys. Rev. Lett.*, 1997, **78**, 1396.

11 W. W. Schoeller, *J. Mol. Struct. THEOCHEM*, 2010, **957**, 66–71.

12 S. Grimme, S. Ehrlich and L. Goerigk, *J. Comput. Chem.*, 2011, **32**, 1456–1465.

13 E. van van Lenthe, E. J. Baerends and J. G. Snijders, *J. Chem. Phys.*, 1993, **99**, 4597–4610.

14 E. van Lenthe, E. J. Baerends and J. G. Snijders, *J. Chem. Phys.*, 1994, **101**, 9783–9792.

15 E. van Lenthe, R. van Leeuwen, E. J. Baerends and J. G. Snijders, *Int. J. Quantum Chem.*, 1996, **57**, 281–293.

16 E. van Lenthe and E. J. Baerends, *J. Comput. Chem.*, 2003, **24**, 1142–1156.

17 M. Ernzerhof and G. E. Scuseria, *J. Chem. Phys.*, 1999, **110**, 5029–5036.

18 C. Adamo and V. Barone, *J. Chem. Phys.*, 1999, **110**, 6158–6170.

19 C. Riplinger and F. Neese, *J. Chem. Phys.*, 2013, **138**, 34106.

20 C. Riplinger, B. Sandhoefer, A. Hansen and F. Neese, *J. Chem. Phys.*, 2013, **139**, 134101.

21 C. Riplinger, P. Pinski, U. Becker, E. F. Valeev and F. Neese, *J. Chem. Phys.*, 2016, **144**, 24109.

22 M. Saitow, U. Becker, C. Riplinger, E. F. Valeev and F. Neese, *J. Chem. Phys.*, 2017, **146**, 164105.

23 Y. Guo, C. Riplinger, U. Becker, D. G. Liakos, Y. Minenkov, L. Cavallo and F. Neese, *J. Chem. Phys.*, 2018, **148**, 11101.

24 F. Weigend, *Phys. Chem. Chem. Phys.*, 2002, **4**, 4285–4291.

25 F. Neese, *WIREs Comput. Mol. Sci.*, 2018, **8**, e1327.

26 F. Neese, F. Wennmohs, U. Becker and C. Riplinger, *J. Chem. Phys.*, 2020, **152**, 224108.

27 H. S. Tam and M. D. Harmony, *J. Phys. Chem.*, 1991, **95**, 9267–9272.

28 O. Christiansen, J. Gauss and B. Schimmelpfennig, *Phys. Chem. Chem. Phys.*, 2000, **2**, 965–971.

29 D. A. Pantazis and F. Neese, *J. Chem. Theory Comput.*, 2011, **7**, 677–684.

30 D. A. Pantazis, X.-Y. Chen, C. R. Landis and F. Neese, *J. Chem. Theory Comput.*, 2008, **4**, 908–919.

31 G. L. Stoychev, A. A. Auer and F. Neese, *J. Chem. Theory Comput.*, 2017, **13**, 554–562.
